# Ethynylene‐Linked 1,2‐Dihydro‐1,2‐Azaborinines With High Energy Densities for Efficient Molecular Solar Thermal Energy Storage

**DOI:** 10.1002/cssc.202600003

**Published:** 2026-04-22

**Authors:** Ralf Einholz, Alexandra Riegger, Virinder Bhagat, Holger F. Bettinger

**Affiliations:** ^1^ Institut für Organische Chemie Eberhard Karls Universität Tübingen Tübingen Germany

**Keywords:** 1,2‐dihydro‐1,2‐azaborinines, high energy density, molecular solar thermal  energy storage, photoizomerization, photoswitches, thermal analysis

## Abstract

Molecular solar thermal energy storage (MOST) systems offer an approach to harnessing solar energy by converting, storing, and releasing it within chemical bonds. This study introduces ethynylene‐linked 1,2‐dihydro‐1,2‐azaborinines as highly promising candidates for MOST systems, exhibiting exceptional energy densities and extended storage times. With an outstanding energy density of 1229 kJ/kg, a half‐life exceeding 36 days, and an absorption onset wavelength of 350 nm, these dihydroazaborinines satisfy the key criteria for optimal MOST performance. Their remarkable properties make them a highly promising basis for developing materials capable of efficiently converting and storing solar energy.

## 1 Introduction

The urgent need for sustainable energy solutions is a critical scientific and societal challenge, driven by economic and environmental concerns. The ever‐growing energy demands of modern society, coupled with dwindling resources and the effects of fossil fuel reliance on pollution and climate change, necessitate the development of innovative and resource‐efficient approaches for energy generation and storage. A particular focus must be placed on environmentally friendly and renewable energy technologies.

Solar energy, an inexhaustible source of energy, holds immense potential. While photovoltaic and solar thermal power generation systems are promising, their dependence on the availability of sunlight requires efficient energy storage solutions [1]. Molecular solar thermal (MOST) systems, which utilize photoisomerization to store solar energy in molecules, offer a promising way to address this challenge [2, 3, 4, 5, 6, 7]. MOST systems rely on the conversion of molecules into a higher‐energy isomer through light absorption, storing the energy until released by a heat trigger or catalyst. This approach has received significant interest due to its potential for high energy storage density and efficient energy conversion [8, 9].

**SCHEME 1:**
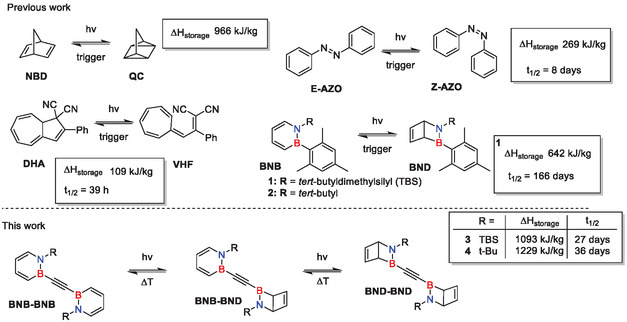
Examples of MOST systems described in the literature and in this work. The half‐lives t_1/2_ are given at 25°C.

However, implementing this concept faces challenges. The light harvesting molecule must efficiently absorb a broad range of the solar spectrum to generate the photoisomer while the photoisomer must be stable and photochemically relatively inactive. Furthermore, the photoconversion step should be associated with enthalpy changes ideally higher than 300 kJ/kg [10]. The thermal release step should be kinetically hindered and ideally be triggered by a suitable catalyst [10, 11]. Several organic photoswitchable couples have been studied extensively (see Scheme 1 for examples), like norbornadiene‐quadricyclane (**NBD/QC**) [12, 13, 14, 15, 16], the E and Z isomers of azobenzene (**E/Z‐azo**) [5, 6, 17, 18, 19], and dihydroazulene‐vinylheptafulvene (**DHA/VHF**) [20, 21, 22]. Further, phase change materials like azopyridines [23] and hydrazones [24] have also been studied in the context of MOST. Each system offers benefits, but no molecular couple fulfills all requirements for the ideal MOST system.

Among them, the parent **NBD/QC** system that does not absorb visible light exhibits a high energy storage potential close to 1 MJ/kg [12]. Energy release can be facilitated through controlled thermolysis, photolysis, or catalysis, yielding heat. For ethynylene linked NBD/QC systems, gravimetric energy densities of 600–800 kJ/kg were predicted [14, 15]. Recent research by Schulte et al. introduced a system of three NBDs coupled to a phenyl ring. This system absorbs in the UVA region and achieves a gravimetric energy storage density of 734 kJ/kg [13].

A new member of the MOST family was discovered to be 1,2‐disubstituted 1,2‐dihydro‐1,2‐azaborinines, BN‐benzenes (**BNB**), that switch to their Dewar isomers (2‐aza‐3‐borabicyclo [2.2.0]hex‐5‐enes, **BND**) upon irradiation with UV light. The **BNB/BND** couples demonstrated the potential to match or even surpass the performance of established MOST systems [25]. The **BNB/BND** couple **1** revealed a gravimetric energy density of 642 kJ/kg with long half‐live [25], while for the **BNB/BND** couple **2** an extensively long half‐live is known (Scheme 1) [26]. Unfortunately, both absorb light only in the UVB region. Recently, our group has reported *bis‐* and *tris‐*ethynylene‐phenylene‐coupled 1,2‐dihydro‐1,2‐azaborinines with gravimetric energy densities approaching 1 MJ/kg and offering storage times of several days [27].

This work introduces two 1,2‐dihydro‐1,2‐azaborinine dyads that are connected via an ethynylene bridge at their boron atoms (Scheme 1). Here, we demonstrate that this novel subclass of **BNB** systems shows very high gravimetric energy densities of up to 1229 kJ/kg with suitable half‐life of more than 1 month. Additionally, a red shift into the UVA region was achieved compared to previously reported systems.

## 2 Results and Discussion

### 2.1 Synthesis

**SCHEME 2:**
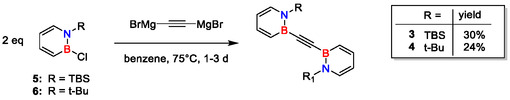
Synthetic route for ethynylene‐linked dihydroazaborinines.

The synthesis of the ethynylene‐linked dihydroazaborinines **3** and **4** was achieved by treating their corresponding B‐chloro‐1,2‐dihydroazaborinines [26, 28, 29, 30, 31] **5** and **6** with 1,2‐ethynediyl‐‘double Grignard’ [32] in moderate yields (see Scheme 2). The compounds were fully characterized by NMR spectroscopy, high resolution mass spectrometry, and UV–Vis spectroscopy.

Satisfyingly, compounds **3** and **4** can be kept under ambient conditions for several weeks. This contrasts with the reported N‐*tert*‐butyl‐B‐phenylethynyl substituted 1,2‐dihydro‐1,2‐azaborinine that degrades already upon contact with water by 3% and with oxygen by 8% within 4 h [30].

### 2.2 Photoisomerization of Dihydroazaborinine Dyads

**FIGURE 1:**
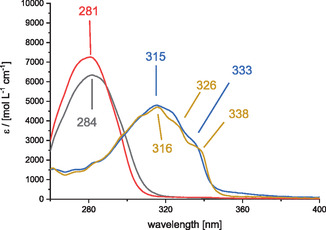
UV absorption spectra of **1** (black), **2** (red), **3** (blue), and **4** (light brown) in cyclohexane.

The UV–Vis absorption spectra of all diyhdroazaborinines presented in this study were recorded in cyclohexane (Figure 1). This solvent was chosen for its ability to provide a wide spectral window for UV/vis analysis.

The introduction of the ethynylene linker in dihydroazaborinines **3** and **4** resulted in a bathochromic shift of their UV–Vis absorption spectrum compared to B‐mesityl substituted **1** and **2**. This shift is attributed to the extended π‐conjugation introduced by the ethynylene bridge. Notably, both compounds **3** and **4** exhibit similar absorption onsets around 350 nm. Compound **3** shows a maximum at 315 nm with a shoulder at 333 nm, while compound **4** displays a maximum at 316 nm with shoulders at 326 and 338 nm.

**FIGURE 2:**
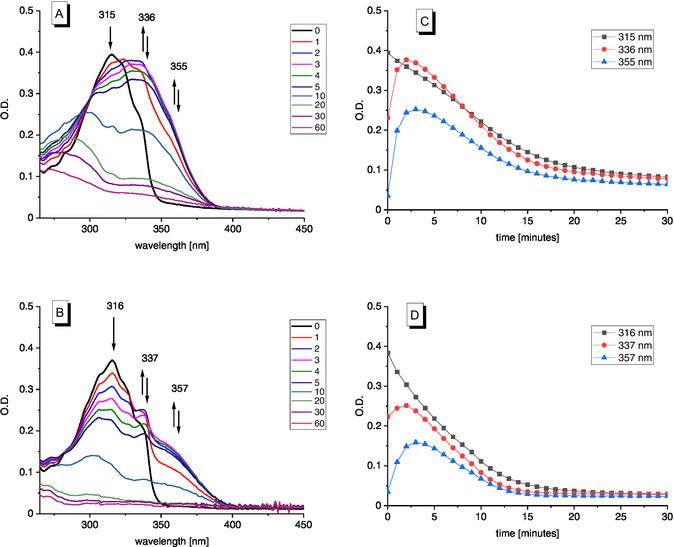
UV–Vis spectra of **3** (A) and **4** (B) during irradiation with λ = 325 nm and plot of prominent absorption bands versus irradiation time in minutes of **3** (C) and **4** (D).

The photoisomerization of the dihydroazaborinines **3** and **4** was monitored using UV–Vis spectroscopy in cyclohexane. Samples were continuously irradiated using a fiber‐coupled 325 nm LED and spectra were recorded in defined intervals (Figure 2).

The UV–Vis absorption spectra of both dyads undergo significant changes. Within the first 3 min, both compounds show the rapid formation of new absorption bands in the 330–375 nm region. Concurrently, the absorption bands at 315 nm (for **3**) and 316 nm (for **4**) decreased gradually. The newly formed bands partially overlapped with the absorption features of the starting dihydroazaborinine materials (**BNB‐BNB**). Continued irradiation resulted in the gradual decline of both the original and intermediate bands, resulting in complete conversion (Figure 2).

**FIGURE 3:**
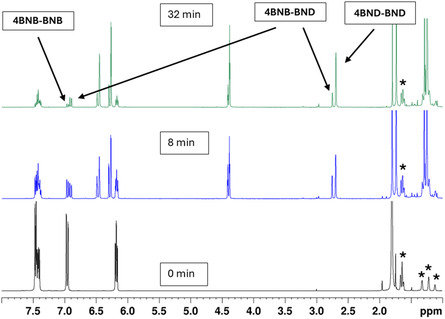
^1^H NMR spectra of compound **4** in methyl cyclohexane‐d_12_. Shown are spectra recorded before irradiation (black), after 8 min of irradiation with 280–400 nm light (blue), and after a total irradiation time of 32 min (green). The arrows indicate the signals corresponding to protons adjacent to the boron atom in each isomer. The asterisks mark residual solvent signals.

As NMR spectra of the photoisomerization processes do not show formation of any other products than **BNB**‐**BND** intermediates (see Figure 3), we assign the initially formed absorption bands to these intermediates. The boron atom in the **BND** form is more electrophilic than it is in the heteroaromatic **BNB** isomer, thus exerting a bathochromic shift on the absorption maximum of the **BNB** unit of the **BNB**‐**BND** intermediate. Once both units are switched to **BND**‐**BND**, the large conjugated system disappeared resulting in hypsochromic shift of the absorption compared to **BNB**‐**BNB**.

Photoisomerization reactions were also performed in quartz J. Young NMR tubes by irradiating 25–50 mM solutions of the compounds in cyclohexane with light in the 280–400 nm range, emitted by a high‐pressure mercury lamp combined with a dichroic mirror. The progress of the reaction was monitored by both ^1^H and ^11^B NMR spectroscopy and irradiation was continued until no further spectral changes were observed. Upon irradiation, two distinguishable NMR signals appeared for each Dewar bridgehead proton. Additionally, the signals corresponding to the protons of the dihydroazaborinine subunits also split into two. Compound **3** was completely converted to its double‐Dewar (**BND‐BND**) isomer within 12 min. For compounds **1** and **2** the photoreaction also proceeded to completion under similar concentration conditions. In contrast, irradiation of compound **4** consistently resulted in a mixture of unswitched (**BNB‐BNB**), singly switched (**BNB‐BND**), and doubly switched (**BND‐BND**) isomers, even after prolonged irradiation (see Figure 3).

The **BNB‐BND** and doubly switched **BND‐BND** isomers of both dyads **3** and **4** were characterized by NMR spectroscopy. All isomerization ratios discussed are also based on NMR data and are detailed in Supporting Information (SI) Section 5. The time‐dependent initial growth and subsequent decline of the signals assigned to the **BNB‐BND** intermediate support the hypothesis – as also observed in UV/Vis spectroscopy – that photoisomerization of each BNB subunit occurs one after another.

While all **BNB** compounds **1–4** are solids at room temperature, the irradiated samples usually remain as noncrystalizing oils after removal of solvent – except **2BND** which solidifies when stored in freezer for a couple of days. Further, all Dewar isomers of **3** and **4** show partial decomposition upon removal of the solvent at room temperature, even under mild conditions without any vacuum. They remain as slightly orange to brownish oils. Attempts to separate the individual isomers from these mixtures failed due to the high reactivity of the **BND** subunits.

### 2.3 Storage Stability and Self‐Discharge Rate

One of the key advantages of molecular solar thermal energy storage (MOST) over thermophysical systems is its insensitivity to thermal insulation. MOST stores free energy as the chemical energy of a higher‐energy metastable molecule. Consequently, the shelf‐life of a molecular solar thermal battery is mainly determined by the kinetic stability of the metastable isomer at the storage temperature. Of relevance is therefore, the activation energy E_A_ of the exothermic reaction back from **BND**‐**BND** to **BNB**‐**BNB**.

**FIGURE 4:**
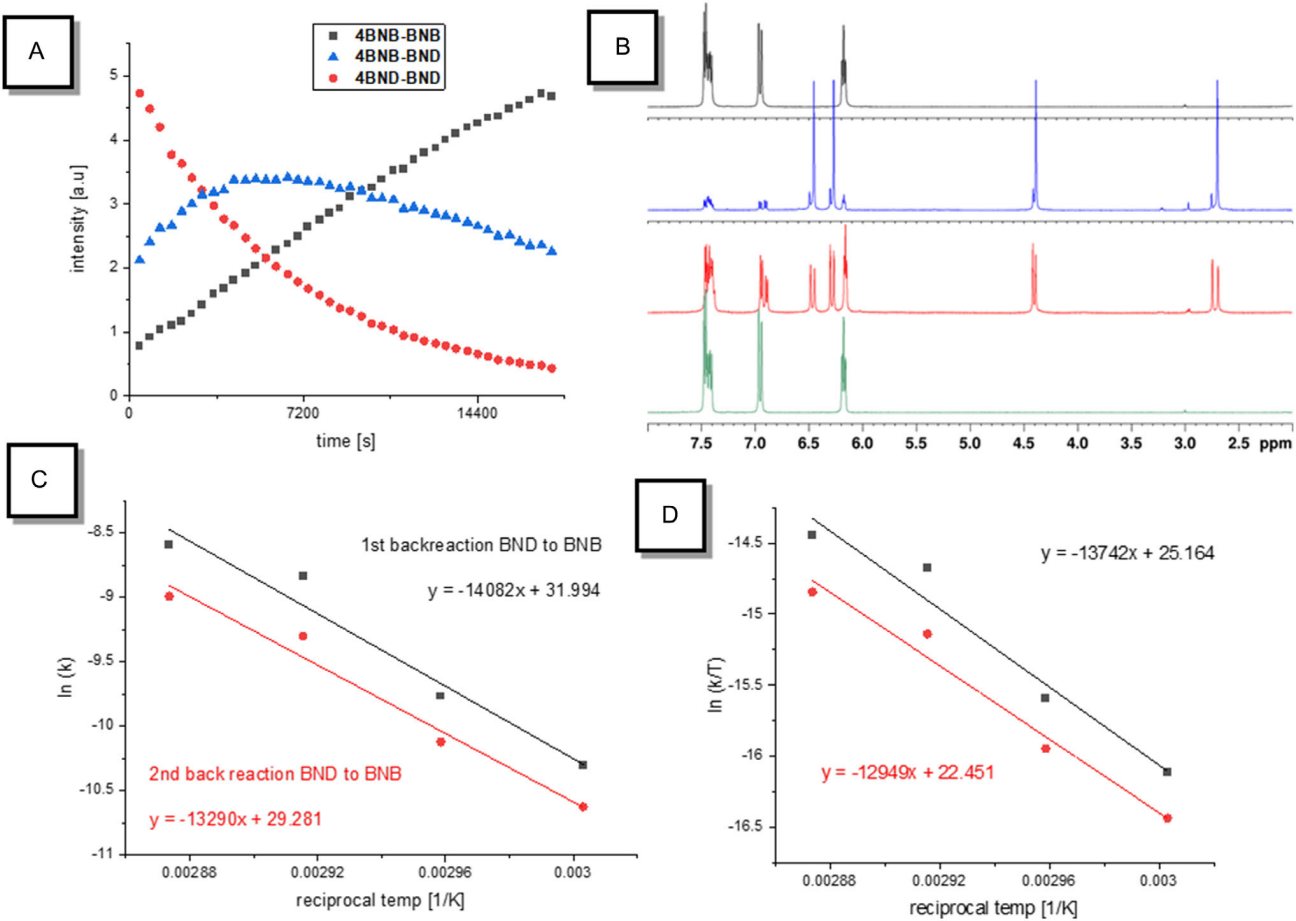
(A) Concentration profile of **4BND‐BND** and its corresponding singly and unswitched isomers upon constant heating at 343 K for 5 h. (B) Partial ^1^H NMR spectra of **4** in cyclohexane‐d_12_. Black: before irradiation; blue: after irradiation with 280–400 nm for 16 min; red: during kinetic measurement of back conversion ‐ after 3 h at 343 K; green: after heating to 343 K overnight. (C) Arrhenius plot for **4** at different temperatures: 348, 343, 338 and 333 K. (D) Eyring plot for **4** at different temperatures: 348 K, 343 K, 338 K, and 333 K.

To determine this barrier, rates of the thermally induced back reaction from **BND**‐**BND** to **BNB**‐**BNB** were measured at four distinct temperatures, monitoring the reaction progress by ^1^H NMR spectroscopy using samples obtained by photolysis as described above. The **BND**‐**BND** isomers transform into the **BNB**‐**BND** isomers following first‐order kinetics. The **BNB**‐**BND** isomer is an intermediate in variable concentration and forms **BNB**‐**BNB** also in a first‐order reaction (see Figure 4A). Arrhenius and Eyring analyses were employed (see Figure 4C,D) to determine activation parameters (E_A_ and log A; Δ*G* ^‡^, Δ*H* ^‡^, Δ*S* ^‡^) and half‐lives at 25°C (t_½_) for all **BND**‐**BND** and **BNB**‐**BND** isomers (see Table 1). After prolonged heating all of the **BND** units have switched back to their **BNB** isomer (Figure 4B).

**TABLE 1:** Kinetic parameters of the thermal ring opening of **BND** isomers of **1**‐**4**. The Arrhenius activation energy E_A_, preexponential factor A, activation enthalpy Δ*H* ^‡^, and free energy of activation Δ*G* ^‡^ at 298 K are given in kJ mol^−1^ while the activation entropy Δ*S* ^‡^ is given in J mol^−1^ K^−1^. The half‐life t_1/2_ in days was determined from the parameters A and E_A_ and is given at 25°C.

	**1** a	**2**	**3**		**4**	
	**BND**	**BND**	**BND**‐**BND**	**BNB**‐**BND**	**BND**‐**BND**	**BNB**‐**BND**
E_A_	113 ± 5	119 ± 1	120 ± 10	120 ± 1	115 ± 16	109 ± 12
log A	28.80	29.39	34.60	34.13	31.99	29.28
Δ*H* ^‡^	110 ± 1	118 ± 9	119 ± 10	119 ± 1	111 ± 17	108 ± 12
Δ*S* ^‡^	−15 ± 0.3	−10.9 ± 1.3	33.5 ± 4.2	29.6 ± 0.5	11.7 ± 2.7	−10.9 ± 2.1
Δ*G* ^‡^	115 ± 1	122 ± 8	109 ± 11	110 ± 2	109 ± 18	111 ± 12
t_1/2_	166	2579^b^	18	27	34	36

a Taken from Edel et al. [25].

b Calculated from the data given by Richter et al. [26].

Interestingly, significantly different activation parameters were found for **3** and **4**. While **3** shows positive entropy of activation for both steps of similar magnitude, **4** reveals that the entropy of activation for the first step is positive and for the second is negative. The conversion of **BNB**‐**BND** to **BNB**‐**BNB** is in both cases the slower step. All half‐lives for **3** and **4** are significantly shorter than those for **1** and **2**. Yet, the compounds allow for the storage of energy over several days at room temperature (see Table 1) being sufficiently long for MOST applications.

### 2.4 Energy Density

The gravimetric energy densities are crucial for storage effectiveness. In MOST, the amount of energy stored per unit mass depends on the change in energy when the molecule isomerizes. Measuring the heat release through differential scanning calorimetry (DSC) is a standard method for characterizing established MOST systems like NBD/QC or azobenzene [4, 7, 13, 14, 15, 16].

To obtain the energy densities of the all‐**BND** systems **1–4**, the dihydroazaborinines were dissolved in 300 µL of high‐boiling solvents such as decalin (for compounds **1**, **3**, and **4**) or dodecane (for compound **2**) and irradiated in a quartz J. Young NMR tube with a high‐pressure mercury lamp emitting wavelengths between 280–400 nm selected by a dichroic mirror. The conversion progress was monitored by ^11^B NMR spectroscopy. Once no further changes were observed, half of the solution was directly used for DSC measurements without removing the solvent. To the remaining solution, deuterated dichloromethane was added for NMR locking, and a ^1^H NMR spectrum was recorded. Since the spectral region of interest is not affected by the excess of nondeuterated solvent, the conversion ratio could be determined from the integrals of the proton signals adjacent to the boron or nitrogen atoms in **BNB** and **BND** subunits. The values obtained from DSC measurements were corrected based on these ratios to ensure that the measured heat release corresponds only to the converted fraction of the sample (for details, see Supporting Information section 7).

We favored DSC measurements in solution over those conducted in the solid state because solution measurements are free from phase transitions or crystallization effects. Performing the measurements in solution minimizes these effects and allows a reproducible thermochemical characterization of the system. The use of a solution ensures a uniform distribution of molecules, resulting in clearer and easier interpretable thermal transitions. Since all of the transformations investigated in this work also occur in solutions, conducting the measurements under these conditions better reflects the actual conversion processes. Experimental details and thermograms of **1–4** without solvent are described in the Supporting Information section 7.

To exclude any contribution from the solvent, a DSC measurement of the solvent only versus empty crucible was performed. This control experiment showed no exothermic or endothermic events in the relevant temperature range, confirming that the observed heat release can be attributed solely to the **BND‐BND** to **BNB‐BNB** conversion (SI, Figure S28).

**FIGURE 5:**
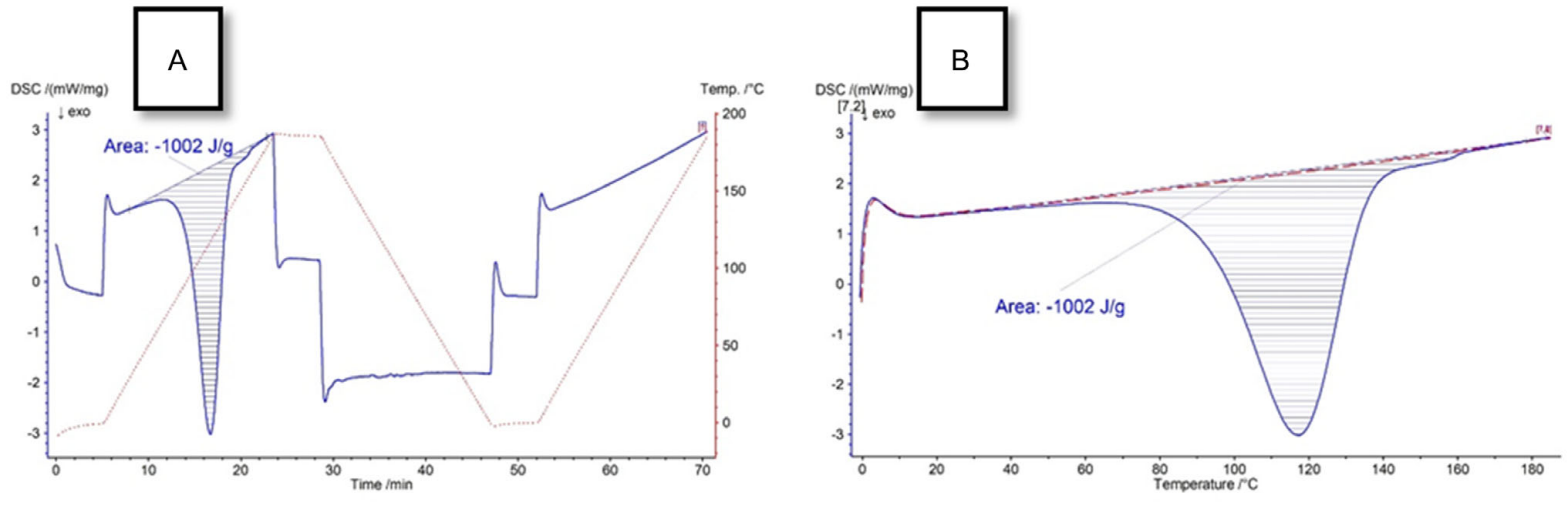
(A) DSC thermogram (blue) of **3** in decalin including heating/cooling profile (red) and (B) detailed comparison of the first (blue) and the second heating cycle (red).

During the first heating cycle, all photoproducts exhibited the characteristic DSC signal pattern of an exothermic process, which was assigned to the thermal back‐conversion of **BND‐BND** to their **BNB‐BNB** isomers. To ensure completeness of the energy release, the maximum temperature 185°C for **1**, **3** and **4**, and 210°C for **2** was held for 5 min in each experiment. No further exothermic events were observed during the isothermal hold. Additionally, the samples were subsequently cooled in the device to 20°C for **1** and **2** and to 0°C for **3** and **4**. Then the samples were reheated with identical conditions as before. In all cases, no additional thermal release was detected during the second heating cycle, supporting the conclusion that the ring‐opening process proceeds quantitatively and irreversibly under the applied conditions. A representative thermogram of **3** along with its heating profile is presented in Figure 5. The thermal analysis data for **1–4** can be found in Supporting Information section 7.

After the DSC measurements, all crucibles were opened and extracted with deuterated dichloromethane. The extracts were analyzed by ^1^H and ^11^B NMR spectroscopy to verify that the compounds have completely switched back to their dihydroazaborinine forms and remained intact and undamaged.

We also reinvestigated the thermal behavior of **1** to prove that the results from DSC are comparable with those from reaction calorimetry reported earlier [26]. It is reassuring that the values obtained from DSC measurements in solution show good agreement with data computed at the DLPNO‐CCSD(T)/cc‐pVTZ//M06‐2X/6‐311+G(d,p) level of theory (Table 2, for computational details see SI).

**TABLE 2:** Thermodynamic data derived from DSC and computed Δ*H*_theor_ at the DLPNO‐CCSD(T)/cc‐pVTZ//M06‐2X/6‐311+G(d,p) level of theory.

**Molecule**	**M, g/mol**	**Δ*H*, kJ/mol**	**Δ*H*, kJ/kg**	**Δ*H*** **_theor_** **, kJ/mol**	**Δ*H*** **_theor_** **, kJ/kg**
**1**	311.4	209 ± 4^a^	671	217	697
**2**	253.2	184 ± 4	727	208	822
**3**	408.3	446 ± 19	1093	224 + 235	1124
**4**	292.0	359 ± 9	1229	214 + 215	1469

a The value obtained earlier by reaction calorimetry is 200 ± 4 kJ/mol [26].

## 3 Conclusion

We have demonstrated that ethynylene‐linked 1,2‐dihydro‐1,2‐azaborinines (**BNB‐BNB**) can be photochemically converted into their metastable, high‐energy Dewar isomers (**BND‐BND**). These isomers switch back thermally to their original **BNB‐BNB** form with half‐lives of 18‐36 days at 25°C, which is attractive for MOST storage applications.

Our studies show that the photoisomerization of **BNB** subunits to their **BND** counterparts occurs successively. The thermal back reaction of the individual dihydroazaborinine units from **BND** to the **BNB** proceeds sequentially, indicating a low degree of electronic interaction between the two linked dihydroazaborinine units.

The energy storage densities of the ethynylene‐linked dihydroazaborinines of 1093 kJ/kg and 1229 kJ/kg are exceptionally high. Notably, these values are amongst the highest reported for a molecular solar thermal photoswitch to date.

Recent theoretical studies have shown that substitution at the carbon positions *para* to the heteroatoms significantly influences the absorption wavelength [33]. Our current efforts focus on applying these strategies to red shift the absorption spectrum, scaling up synthetic methodologies, and enhancing the stability of the Dewar isomers.

Our research demonstrates a significant red shift in the absorption spectrum of dihydroazaborinines, combined with outstanding energy storage properties and reasonable storage lifetimes, can be achieved by insightful design of the molecular scaffold. These findings suggest promising potential for this class of photoswitches in solar thermal energy storage applications.

## Note Added in Proof

While this manuscript was under consideration for publication, the Dewar isomer of a pyrimidone was reported to have an energy density of 1.6 MJ/kg [34].

## Author Contributions

R.E. performed all experiments and measurements, including synthesis, spectroscopy, and analysis, wrote the manuscript and acquired some funding. Computations were carried out by A.R., V. B., and H.F.B. H. F. B. developed the research idea, edited the manuscript, and performed funding acquisition.

## Supporting Information

Additional supporting information can be found online in the Supporting Information section.

## Conflicts of Interest

The authors declare no conflicts of interest.

## Supporting information

Supplementary Material

## Data Availability

The Supporting Information contains the data underlying this manuscript.
