# Supplementary material for: Ethynylene‐Linked 1,2‐Dihydro‐1,2‐Azaborinines With High Energy Densities for Efficient Molecular Solar Thermal Energy Storage
Source: ChemSusChem. 2026 Apr 22;19(8):e202600003. doi: 10.1002/cssc.202600003 (PMC13102551; doi:10.1002/cssc.202600003)
Supplement: Supplementary file 1 — Supplementary Material [file CSSC-19-e202600003-s001.pdf]

Supplementary Information

Ethynylene-Linked 1,2-Azaborinines with High Energy Densities for Efficient Molecular Solar Thermal Energy Storage

Ralf Einholz, Alexandra Riegger, Virinder Bhagat, Holger F. Bettinger

1.	Experimental Details	S2
2.	Synthesis	S3
3.	Mass Spectra	S5
4.	NMR Spectra	S6
5.	Characterization of the Dihydroazaborinine Photo-isomers	S13
6.	Kinetic Data	S20
7.	Heat Release (DSC)	S25
8.	Computational Details	S47
9.	Computational Results	S49
10.	Cartesian coordinates	S51
11.	References	S75

1. Experimental Details

All chemical experiments were conducted under an inert atmosphere of nitrogen or argon using standard Schlenk techniques or in a glovebox (mBraun UNIlab LMF and mBraun UNIlab Plus). Solvents for synthesis were dried using a solvent purification system (mBraun MB SPS-800) or purchased from Thermo Fischer scientific (in ExtraDry quality with AcroSeal™). Solvents for chromatography were of HPLC grade (Thermo Fischer).

Chemicals for starting materials were purchased from abcr (*tert*-butyl allylamine), Apollo Scientific (TBSCl), BLDpharm (Grubbs I), Merck, Sigma-Aldrich (Pd/C, BF_3•Et_2O), Thermo Fischer (former Acros organics) (BCl_3, allyl bromide, ethyl magnesium bromide, ethynyl magnesium bromide, cyclohexene) and were used without further purification.

Solvents for NMR spectroscopy were purchased from deutero (cyclohexane-d_{12}, methylcyclohexane-d_{14}, p-xylene-d_{10}) or Merck (CD_2Cl_2, benzene-d_6, toluene-d_{10}).

All commercially available chemicals and solvents were used without further purification.

Flash chromatography was performed on an Interchim Puriflash 430 system equipped with prepacked PF30SIHP columns.

NMR spectra were recorded on a variety of Bruker spectrometers: Avance III HDX 700, Avance III HDX 600, AVIII400HDx, Avance 400, and Avance III HD 300 NanoBay. ^{11}B NMR and ^{13}C NMR experiments were proton-decoupled (unless otherwise noted). Chemical shifts are reported in parts per million (ppm) relative to tetramethylsilane, using the residual NMR solvent signals.

Mass spectrometry analyses were performed by the mass spectrometry department of the the Institut für Organische Chemie, Universität Tübingen. High-resolution mass spectrometry (HR-MS) in atmospheric pressure chemical ionization (APCI) mode was measured using a Bruker maXis 4G instrument. Low-resolution electron ionization mass spectrometry (EI-MS) was recorded using an Agilent MSD 5977 instrument with direct inlet probe (DIP) in selected ion monitoring (SIM) mode. The molecular ion [M+H]+ is reported in m/z units.

Differential scanning calorimetry (DSC) measurements were performed on a NETZSCH DSC 300 Calaris Select instrument using concavus® aluminum crucibles.

UV/vis spectra were recorded on an Avantes AvaSpec ULS2048 EVO-R5 spectrometer with an AvaLight DH-S-BAL light source. Temperature stability was controlled with the sample holder qpod2 from quantum northwest that is also equipped with an integrated stirrer.

The conversion from BNB-BNB to BNB-BND to BND-BND on the NMR scale were conducted using a 500 W high-pressure mercury lamp housed in a *QuantumDesign* system equipped with quartz optics and a dichroic mirror to choose the appropriate wavelengths from 280 nm to 400 nm. This wavelength range was chosen as it matches with the longest absorption band of the investigated 1,2-dihydroazaborinine dyads. The light power was determined to be 1.42 W at 280 nm using a *Thorlabs* power meter *PM 101* with a *S415C* thermal sensor.

Irradiation experiments at UV-Vis scale (<1mM) the conversion from BNB-BNB to BNB-BND to BND-BND, were done with fiber coupled prizmatix Mic-LED-325D (325 nm for **3** and **4**), Mic-LED-280L (280 nm for **1** and **2**) that were connected directly to the sample holder (*Quantum Northwest*

- *qpod2*) with integrated stirrer and thermal control. The light power was determined to be 380 mW at 280 nm using a *Thorlabs* power meter *PM 101* with a *S401C* thermal sensor

.

2. Synthesis

The synthetic procedure for the compounds **1**, **2**, **5** and **6** was already described in an earlier publication.[1] The 1,2-bis(1-(tert-butyldimethylsilyl)-1,2-azaborinin-2(1H)-yl)ethyne (**3**) was synthesized following a procedure of Marwitz et al. with minor modifications.[2]

1,2-bis(1-(tert-butyl)-1,2-azaborinin-2(1H)-yl)ethyne (**4**)

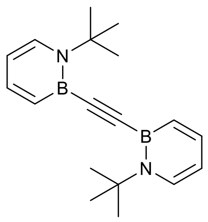

5.5 mL Ethyl magnesium bromide (0.9 M in THF, 5 mmol) and 10 mL ethynyl magnesium bromide (0.5 M in THF, 5 mmol) were combined in a dry Schlenk flask and stirred at rt overnight. All volatiles were removed at reduced pressure and 10 mL benzene were added. To this Grignard mixture was then added a solution of 845 mg (5 mmol) of 1-(tert-butyl)-2-chloro-1,2-dihydro-1,2-azaborinine. The mixture was heated to 75 °C for 3 days. At the conclusion of the reaction, 10 mL water was added, the organic phase was separated, washed with brine and dried with $MgSO_4$. The solvent was removed under reduced pressure. The resulting crude material was subjected to column chromatography (gradient starting from hexane 100 % to hexane/CH_2Cl_2 95:5 %) to yield 1,2-bis(1-(tert-butyl)-1,2-azaborinin-2(1H)-yl)ethyne as white solid (170 mg, 0.58 mmol, 22%).

^{1}H-NMR: 400 MHz, CD_2Cl_2: 7.62 (m, 1H), 7.54 (m, 1H), 6.90 (m, 1H), 6.29 (m, 1H), 1.78(s, 9H).
^{13}C{^{1}H}-NMR: 101 MHz, CD_2Cl_2: 142.2, 135.5, 134.0 (determined by 2D NMR), 110.6, 60.7, 31.5. The signal for the boron-bound alkyne carbon was not detected.
^{11}B{^{1}H}-NMR: 128 MHz, CD_2Cl_2: 27.0.

^{1}H-NMR: 600 MHz, 263 K. Methylcyclohexan-d14: 7.49-7.42 (m, 2H), 6.98 (m, 1H), 6.20 (m, 1H), 1.81 (s, .9H)
^{13}C{^{1}H}-NMR UDEFT: 151 MHz, 263 K. Methylcyclohexan-d14: 142.2, 135.7, 134.6, 121.3, 111.3, 60.8, 32.3.
^{11}B{^{1}H}-NMR: 151 MHz, 263 K. Methylcyclohexan-d14: 27.3.

EI-MS, 70eV: m/z (Int.%): 292.3 (58) [M]+, 236.2 (7) [M-(CH3)2C=CH2)]+, 180.1 (100) [M-(CH3)2C=CH2)-(CH3)2C=CH2)]+.
HRMS APCI m/z: 293.2358 $[M+H]^+$, (calc for $C_{18}H_{27}B_2N_2^+$: 293.2361).

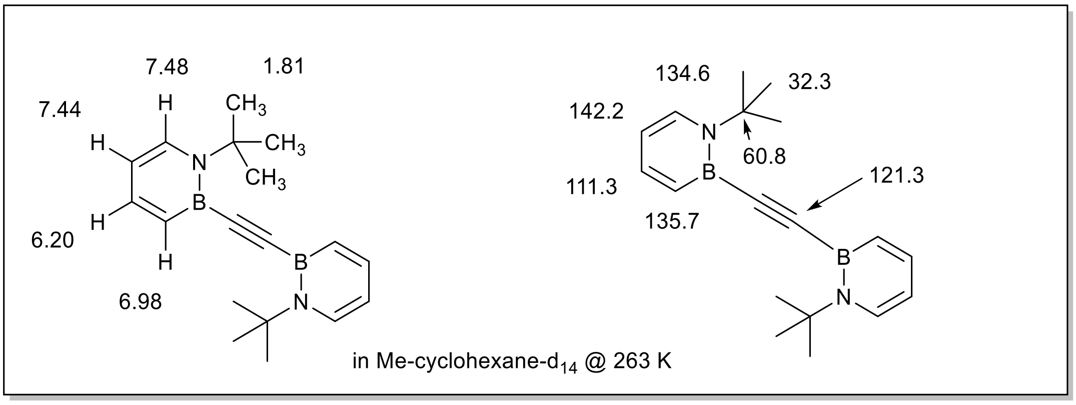

Scheme S1: Assignment of NMR chemical shifts for **4** in methylcyclohexane-d_{14}**.**

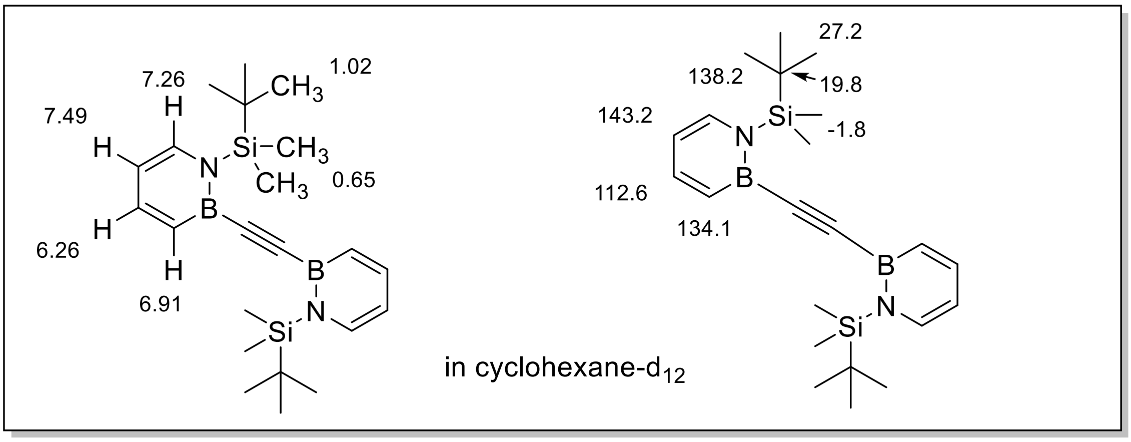

Scheme S2. Assignment of NMR chemical shifts for **3** in cyclohexane-d_{12}.

3. Mass Spectra

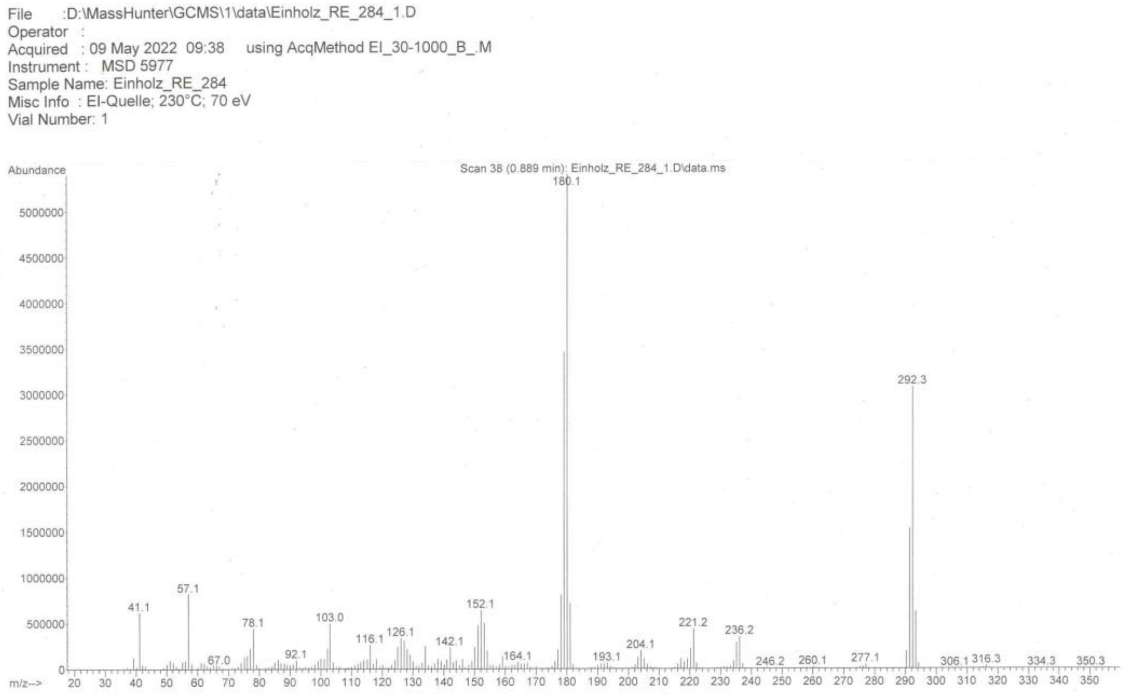

Figure S1. EI-MS spectra of **4**.

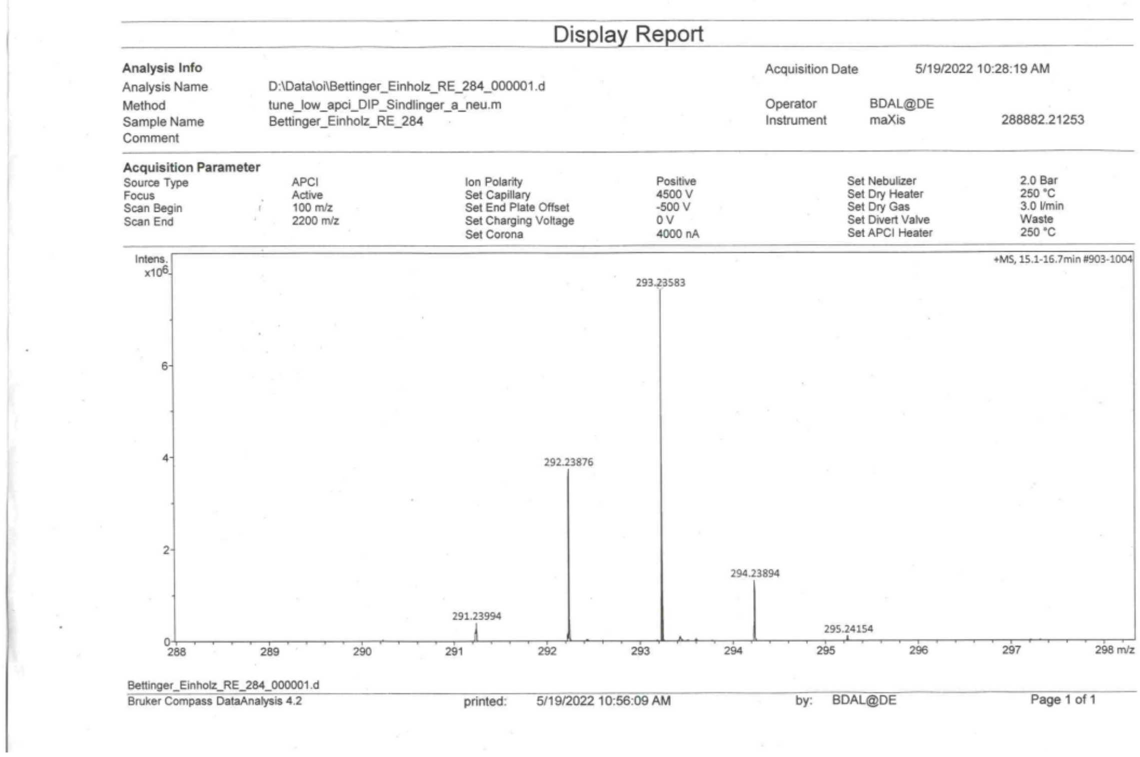

Figure S2. APCI-HRMS spectrum of **4**.

4. NMR-Spectra

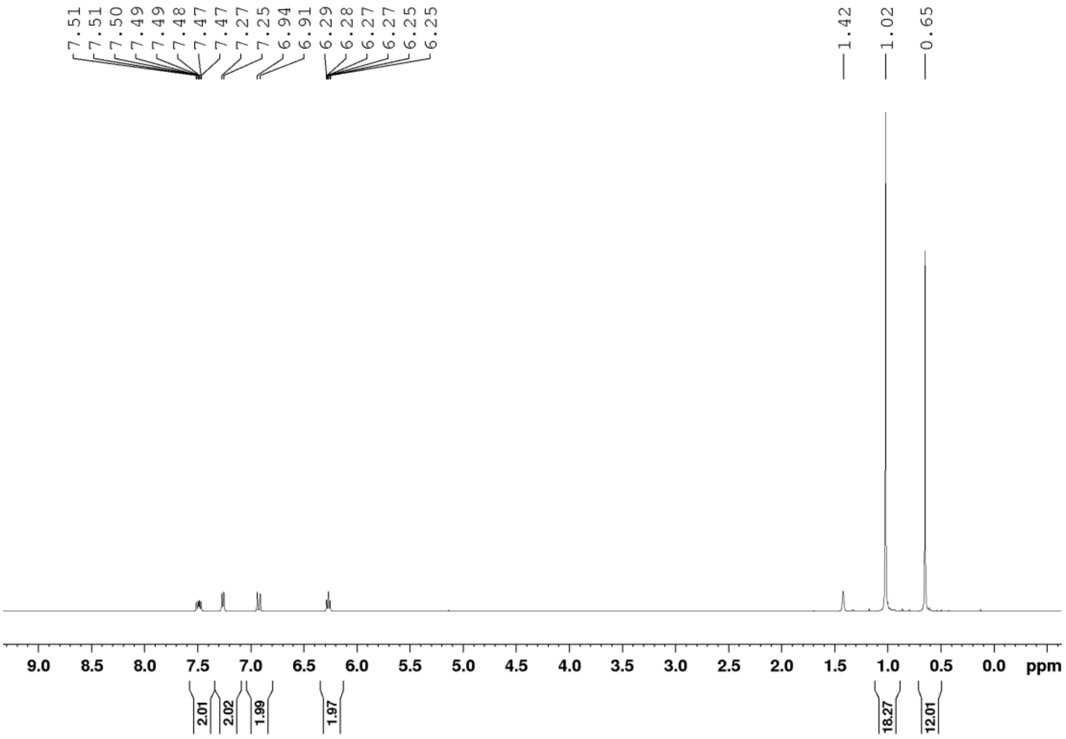

Figure S3. ^{1}H-NMR of **3** in cyclohexane-d_{12}.

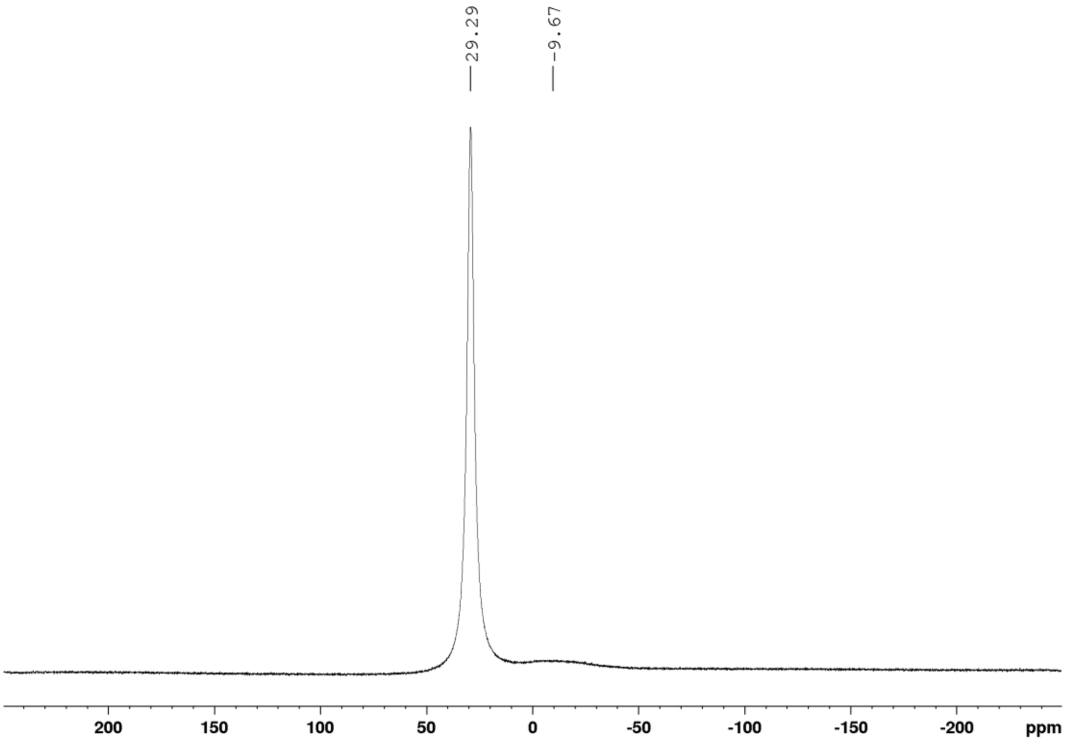

Figure S4. ^{11}B{^{1}H}-NMR of **3** in cyclohexane-d_{12}.

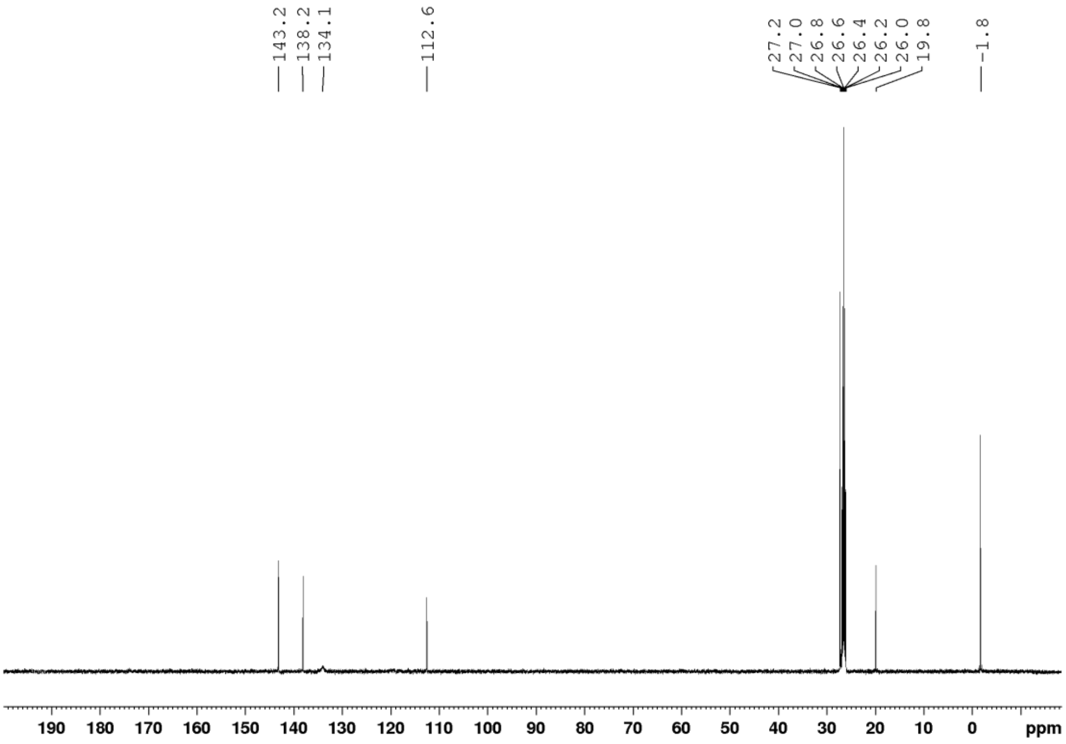

Figure S5. $^{13}C\{^{1}H\}$-NMR of **3** in cyclohexane-d_{12}.

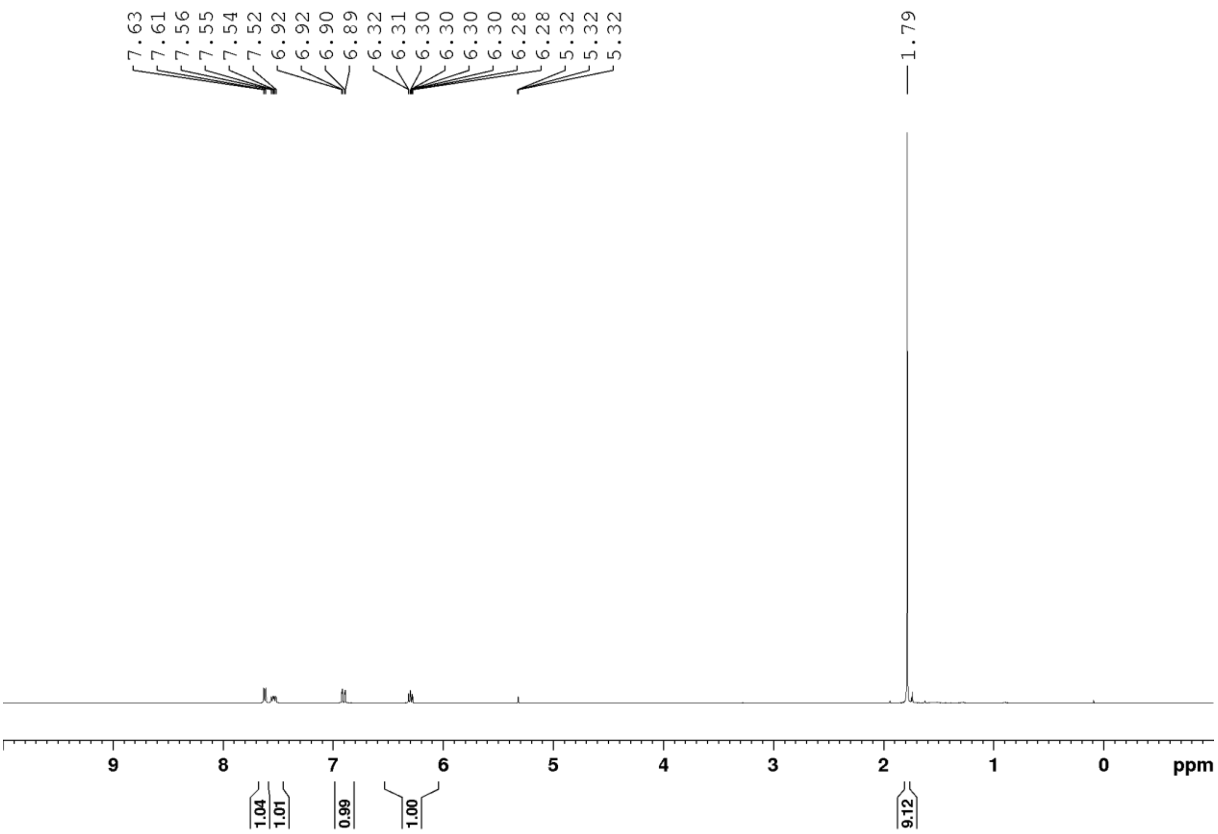

Figure S6. ^{1}H-NMR spectrum of **4** in dichloromethane-d_2.

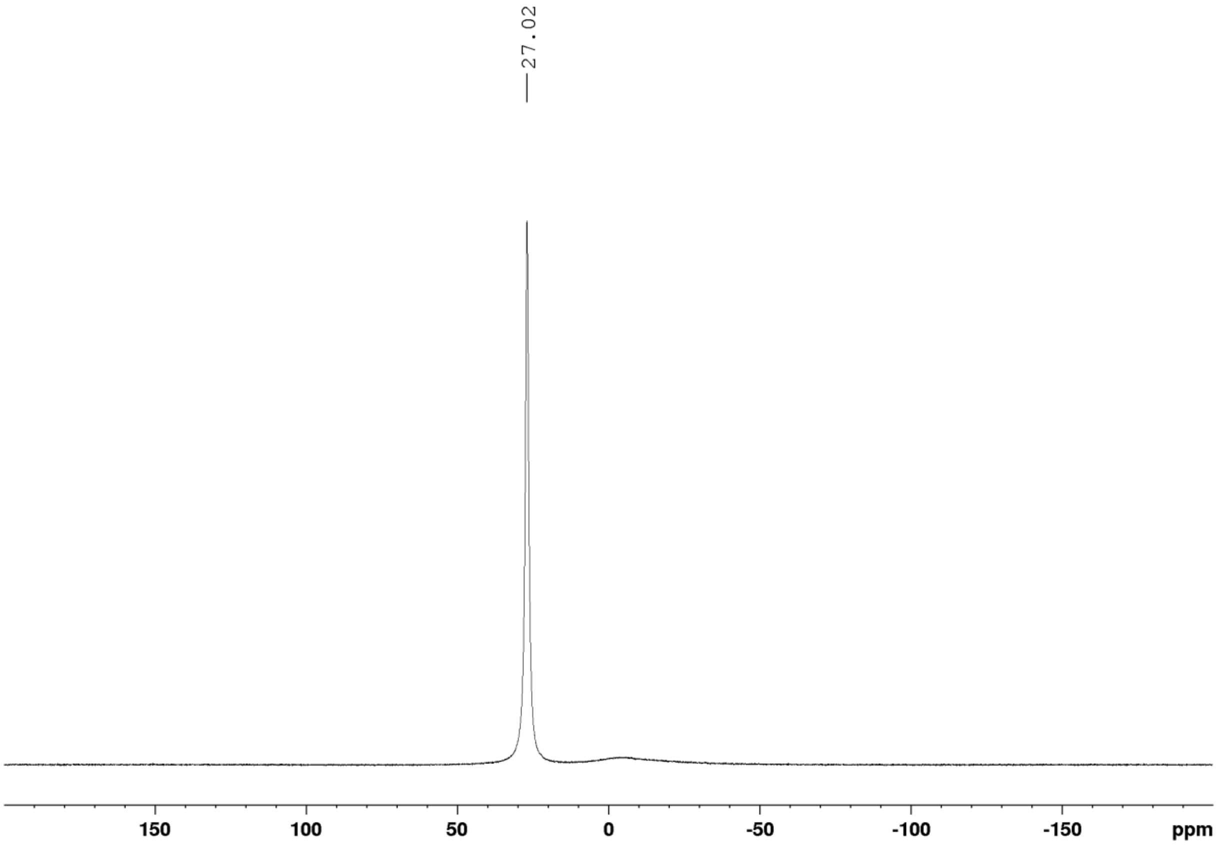

Figure S7. $^{11}B\{^{1}H\}$-NMR spectrum of **4** in dichloromethane-d_2.

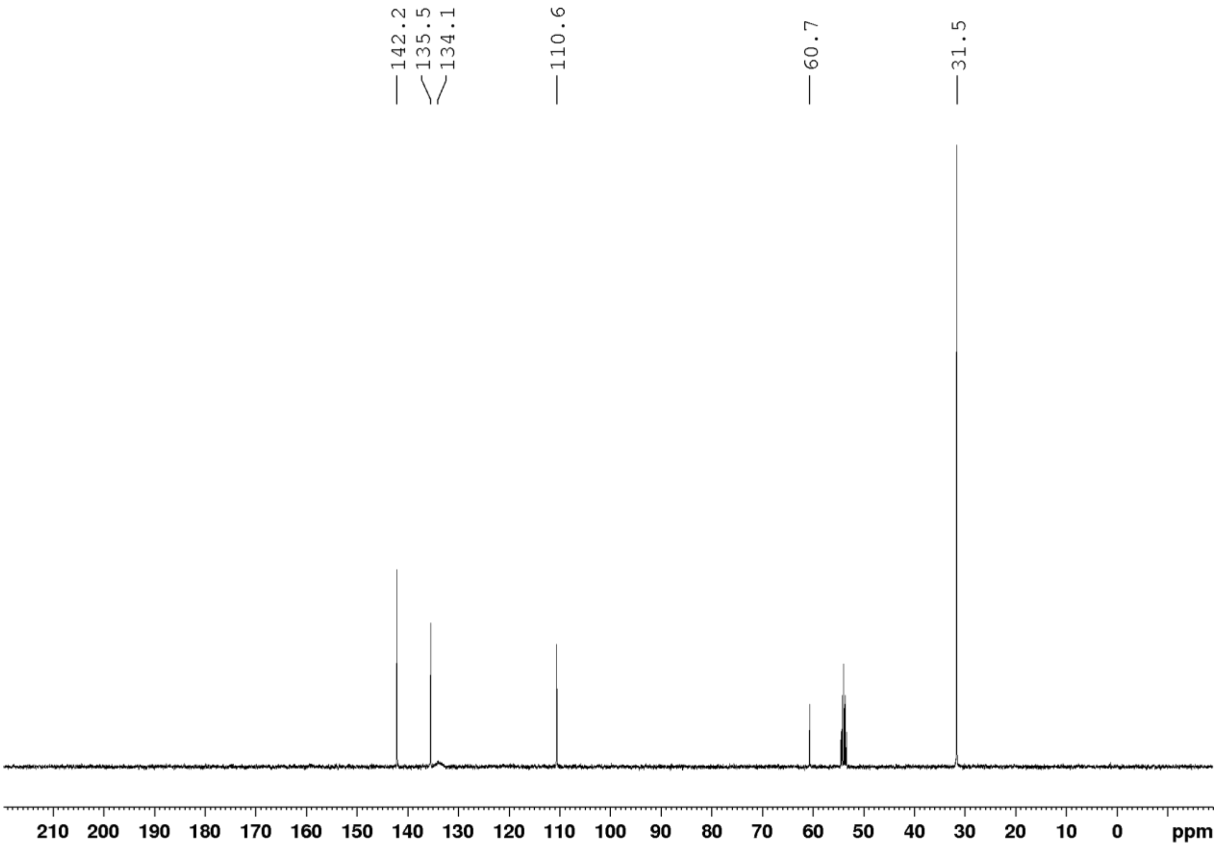

Figure S8. $^{13}C\{^{1}H\}$-UDEFT NMR spectrum of **4** in dichloromethane-d_2.

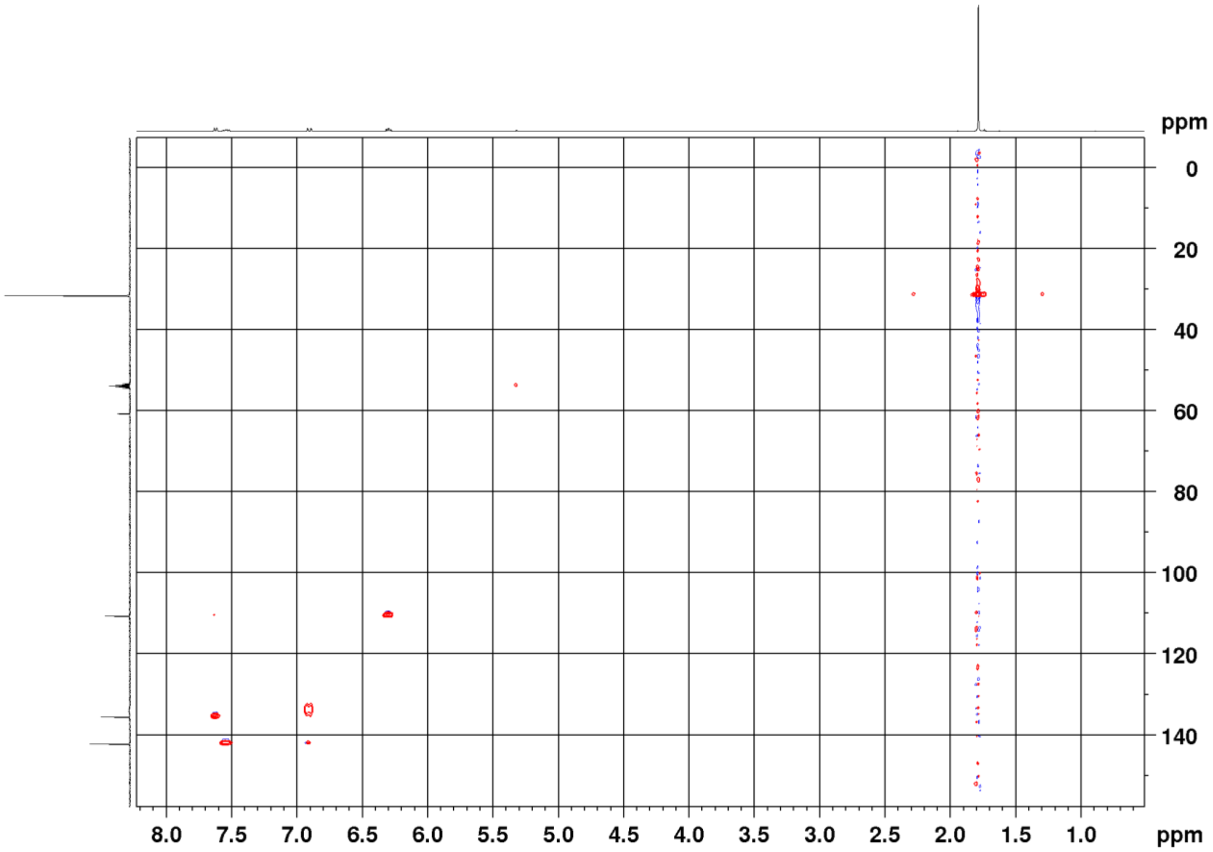

Figure S9. HSQC spectrum of **4** in DCM-d_2

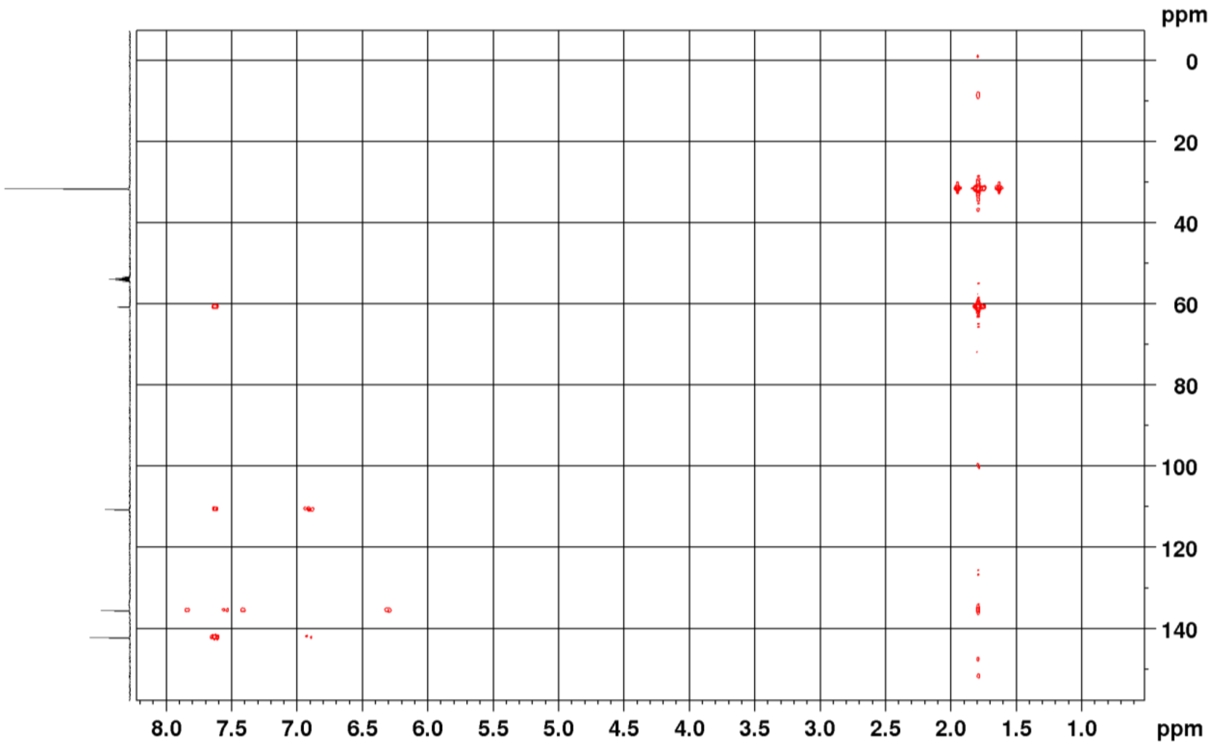

Figure S10. HMBC spectrum of 4 in DCM-d_2.

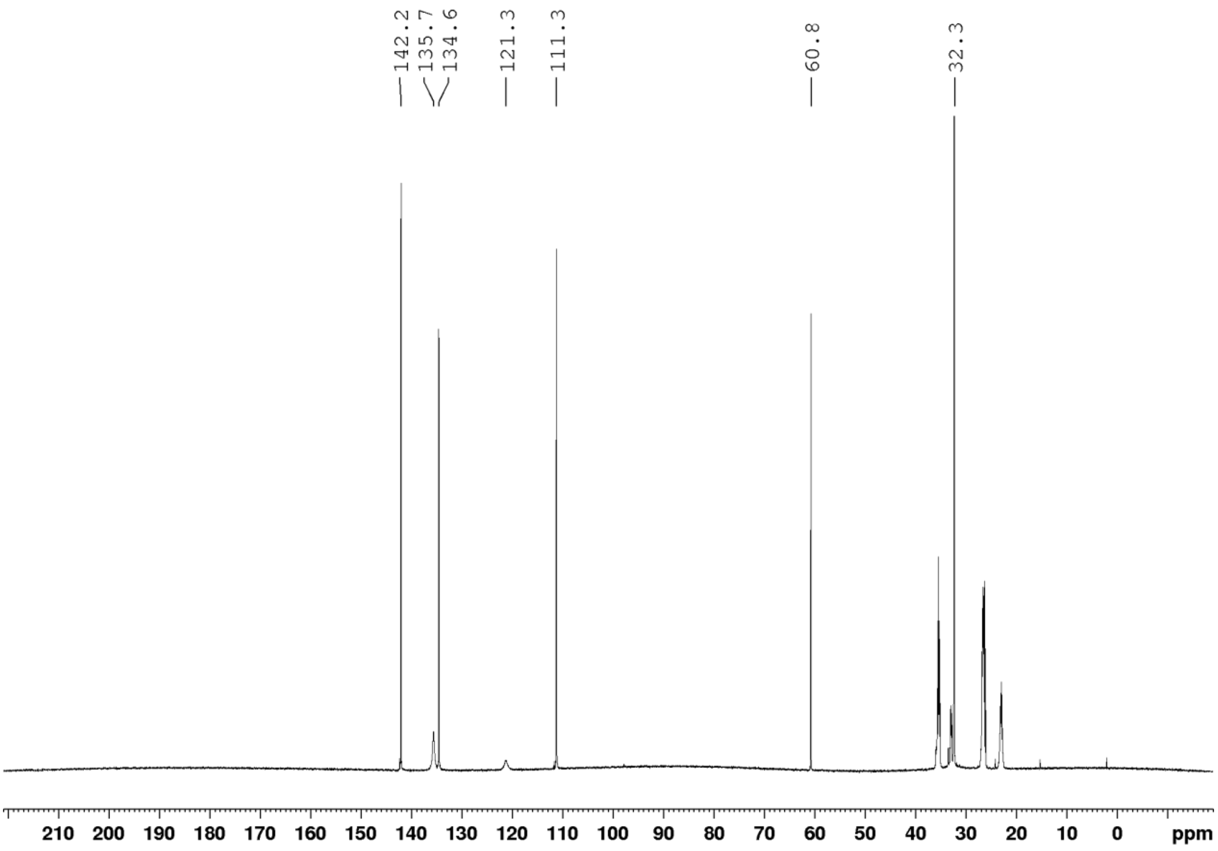

Figure S11. $^{13}C\{^{1}H\}$-UDEFT NMR spectrum of **4** in methylcyclohexane-d_{14} at 263 K.

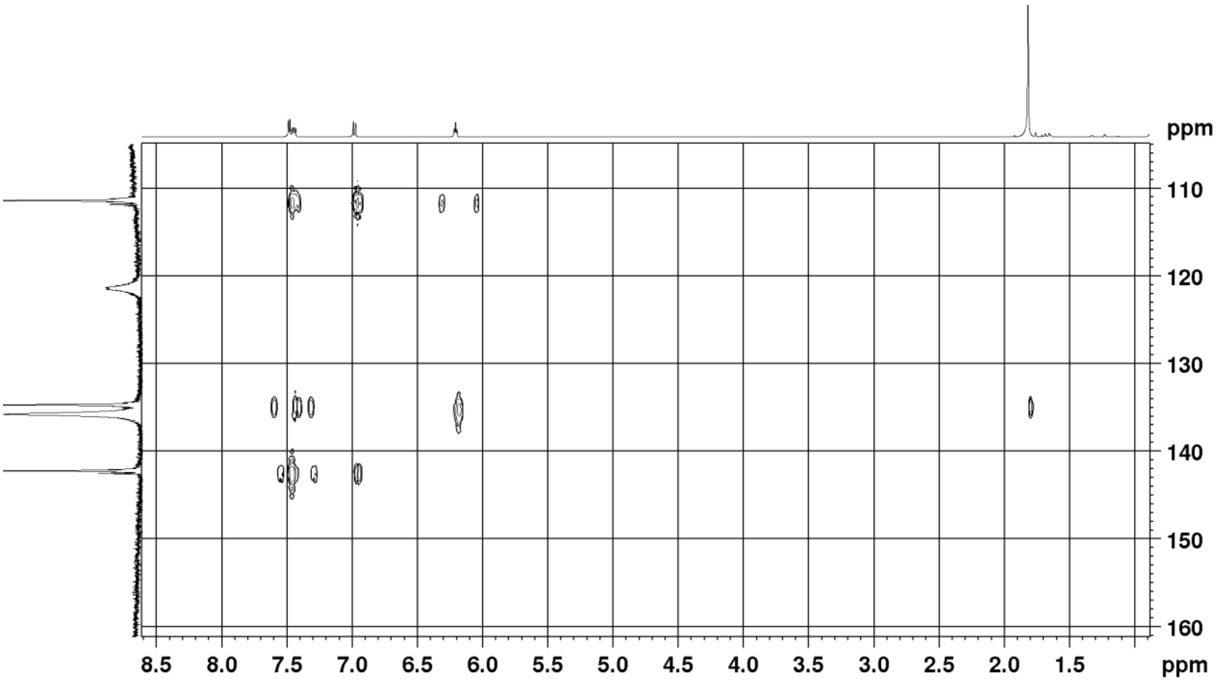

Figure S12. HMBC of **4** in methylcyclohexane-d_{14} at 263 K zoom on aromatic region.

4.1 Stability Check for 3 and 4

We left a small sample of solid **3** and **4** each in an open vial for 3 weeks in the back of the hood. Then the powder was dissolved in deuterated cyclohexane and a ^{1}H NMR spectrum was measured.

To ensure that small signals do not result for decomposition we measured also the ^{1}H NMR spectrum of the cyclohexane-d_{12} that showed some impurities which were found throughout the entire batch (Lot No 27471).

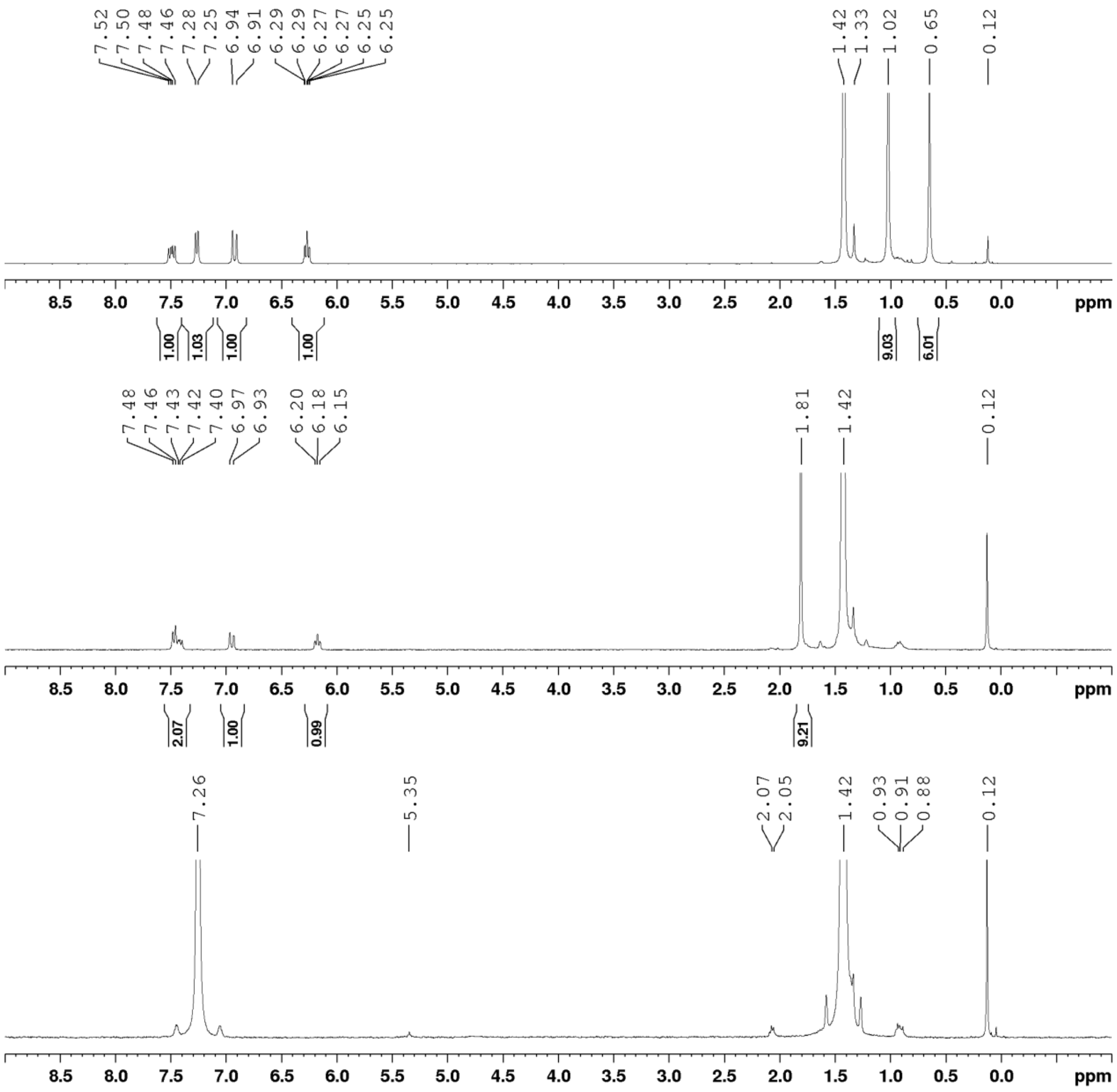

Figure S13. Top: ^{1}H NMR spectrum of **3** measured after keeping it for 3 weeks under ambient conditions. Middle: ^{1}H NMR spectrum of **4** measured after keeping it for 3 weeks under ambient conditions. Bottom: ^{1}H NMR spectrum of the used cyclohexene-d_{12} (Lot No 27471).

5. Characterization of the Dihydroazaborinine Photoisomers

The photoisomers **1BND** and **2BND** were already described and characterized by Richter et al.[1]

To characterize the photoisomers of **3** and **4**, solutions of the respective dihydroazaborinines in cyclohexane-d_{12} (for compound **3**) or methylcyclohexane-d14 (for compound **4**) were irradiated with 280-400 nm light in a quartz J. Young tube for 12-32 minutes until no further changs in the spectra were observed.

The conversion ratio was determined based on the integration of the proton signals adjacent to boron or nitrogen atoms.

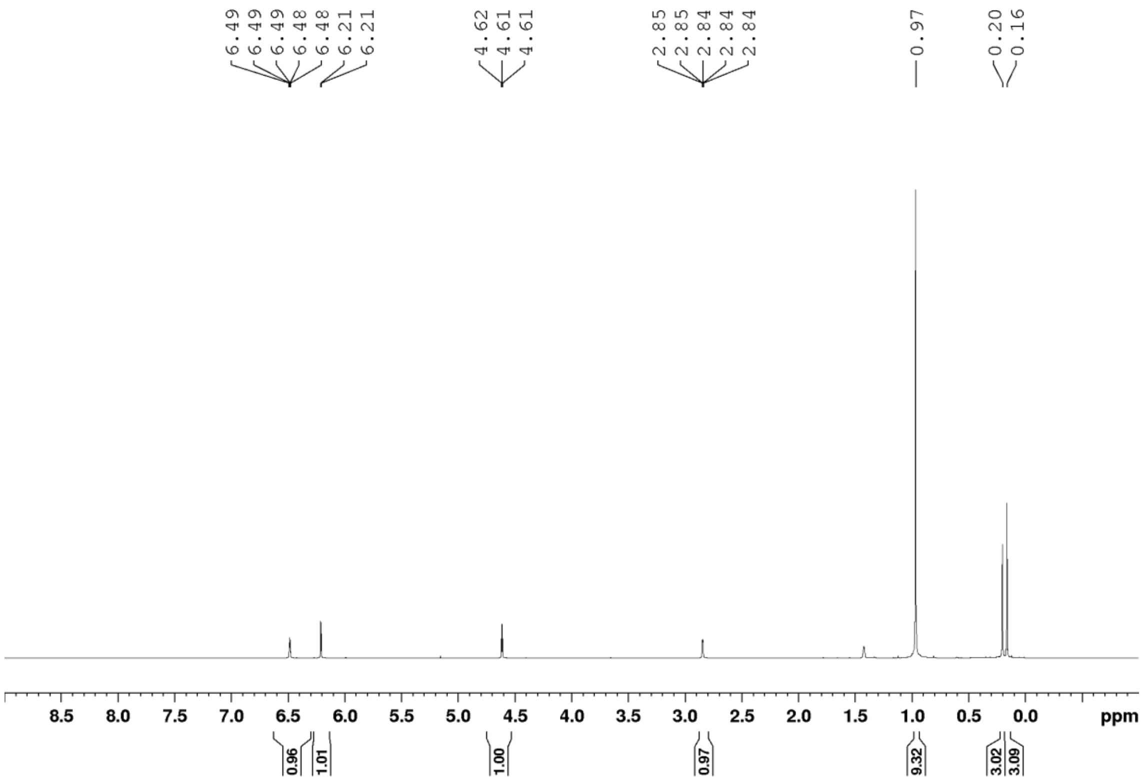

Figure S14. ^{1}H NMR of irradiated **3** in cyclohexane-d_{12}.

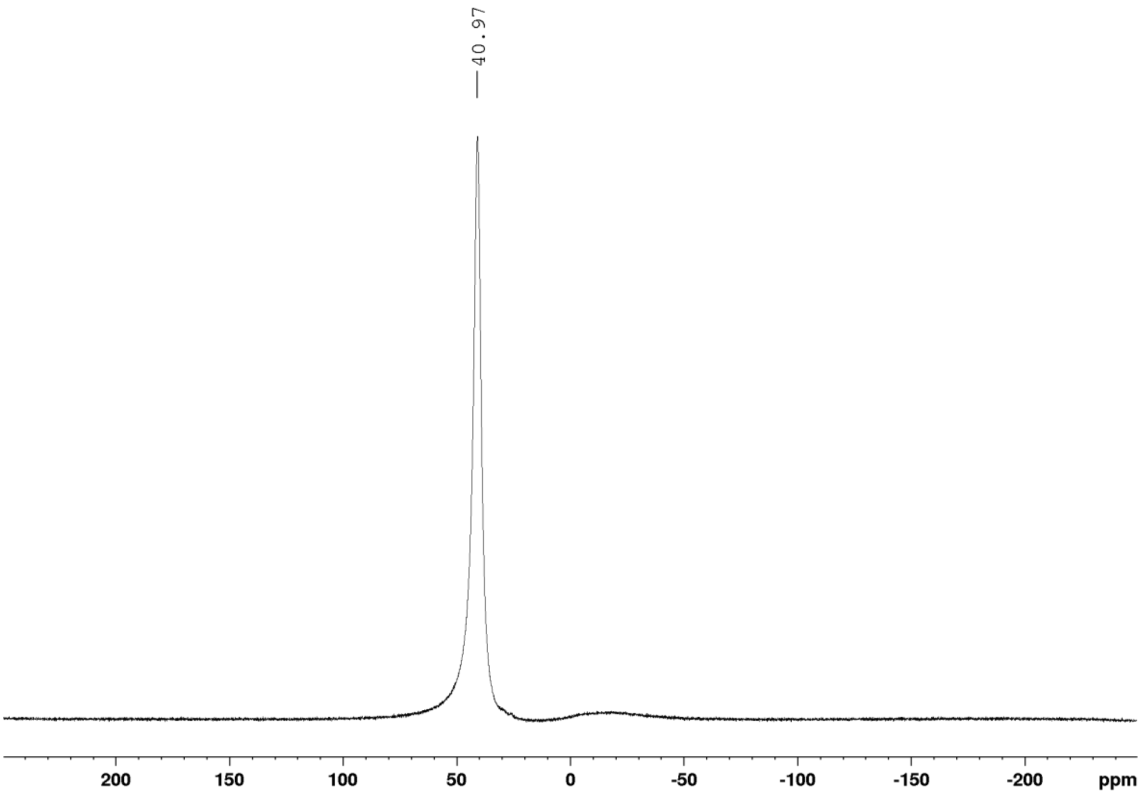

Figure S15. $^{11}B\{^1H\}$ NMR of irradiated **3** in cyclohexane-d_{12}.

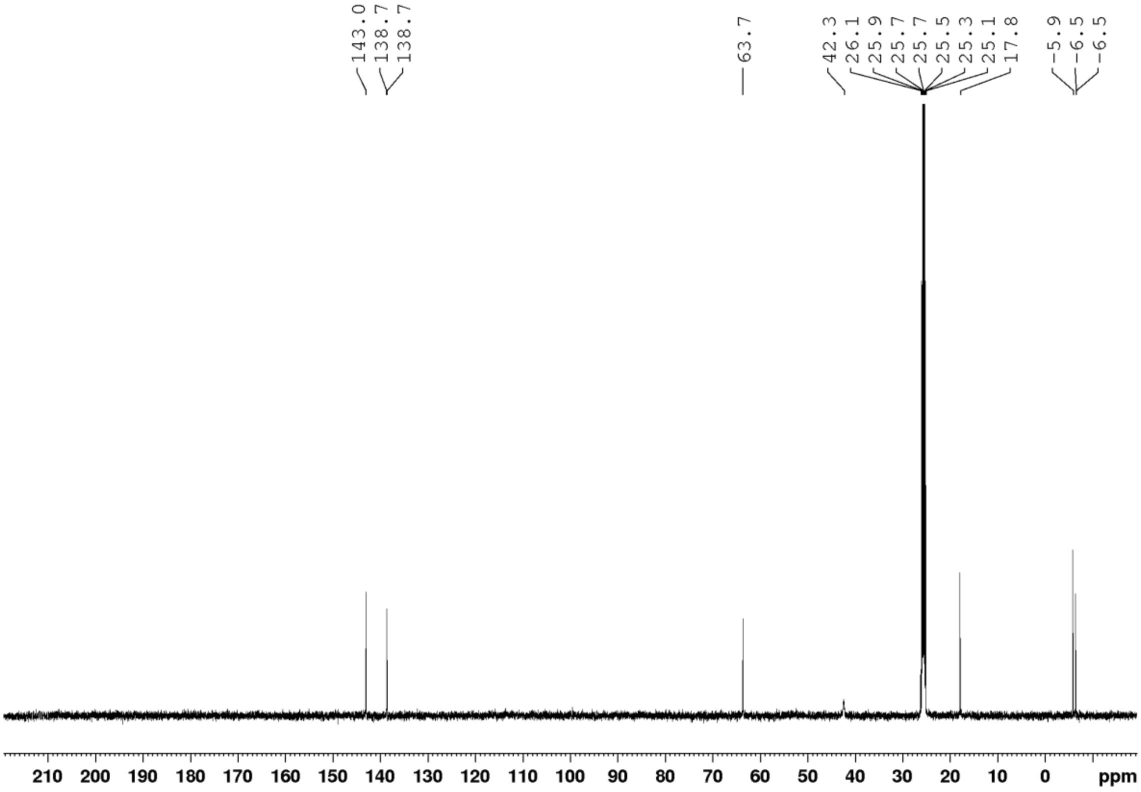

Figure S16. $^{13}C\{^1H\}$ NMR of irradiated **3** in cyclohexane-d_{12}.

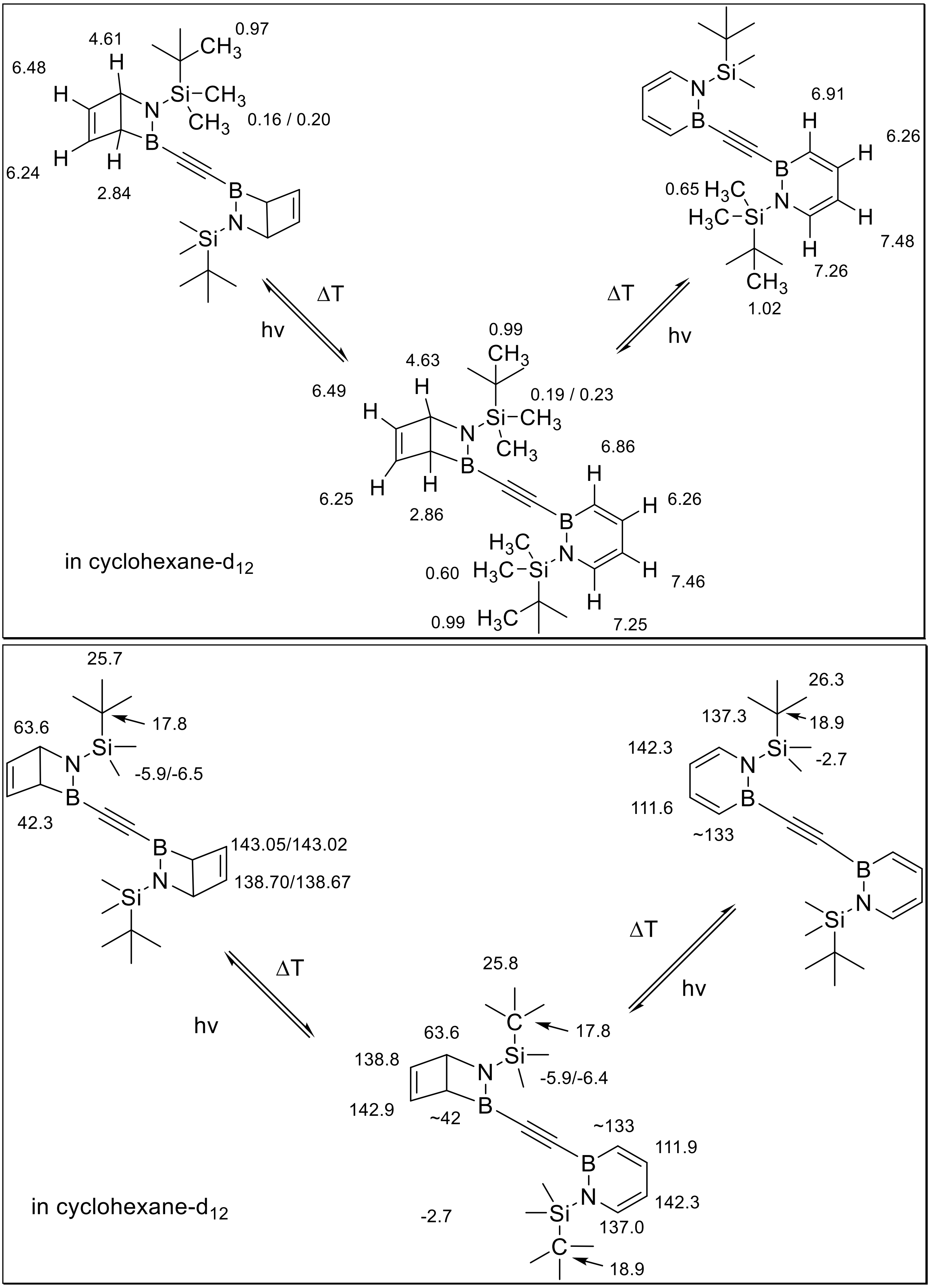

Scheme S3. Assignment of ^{1}H (top) and ^{13}C (bottom) chemical shifts for each photoisomer of **3** in cyclohexane-d_{12}.

The sample of the irradiated compound **4** was measured at 263 K in methylcyclohexane-d_{14}, since the NMR signals corresponding to the ethynylene carbon atoms were not observable at room temperature due to the quadrupole moment of the boron atoms.

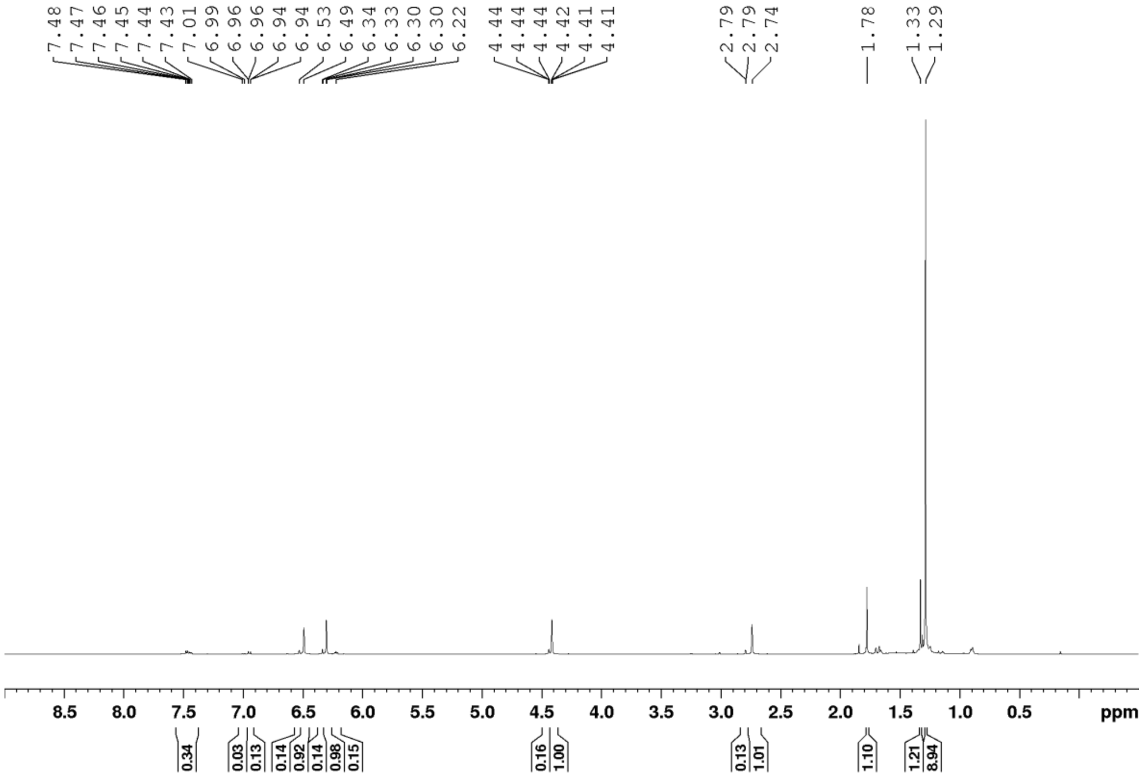

Figure S17. ^{1}H NMR spectrum of irradiated compound **4** in methylcyclohexane-d_{14} at 263 K, showing a mixture of 20 % singly switched (BNB-BND), 78 % doubly switched (BND-BND), and 2 % unswitched (BNB-BNB) isomers of **4**. The ratio was determined based on the integration of the proton signals adjacent to boron: 2.74 ppm corresponds to the doubly switched isomer, 2.79 ppm and 6.94 ppm to the singly switched isomer, and 6.98 ppm to the unswitched isomer.

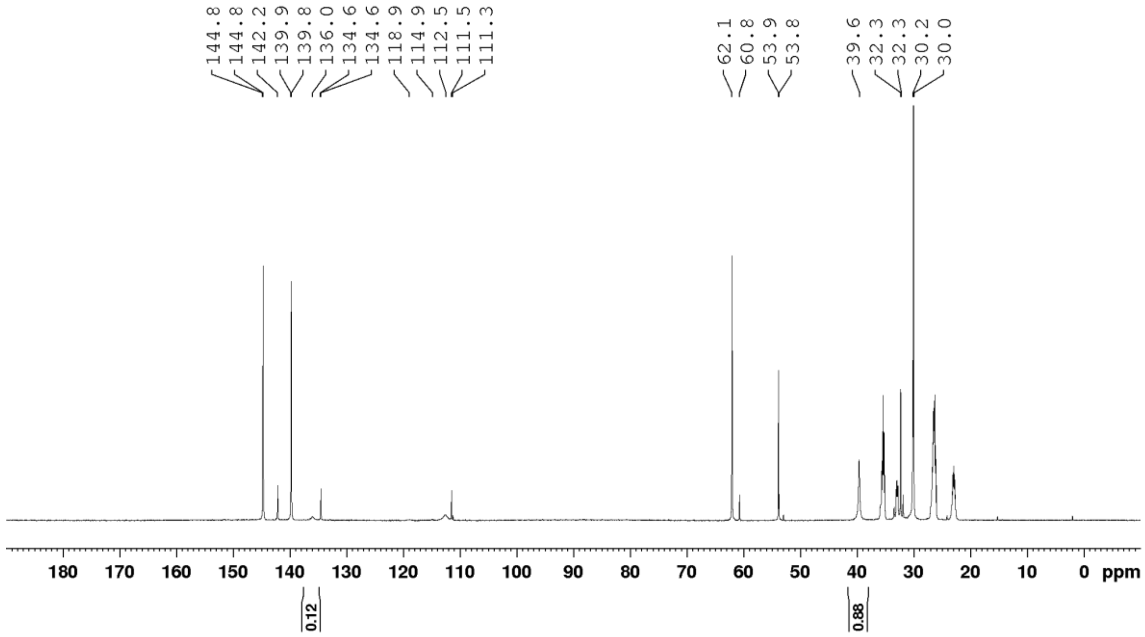

Figure S18. $^{13}C\{^{1}H\}$ NMR of irradiated **4** in methylcyclohexane-d_{14} at 263 K.

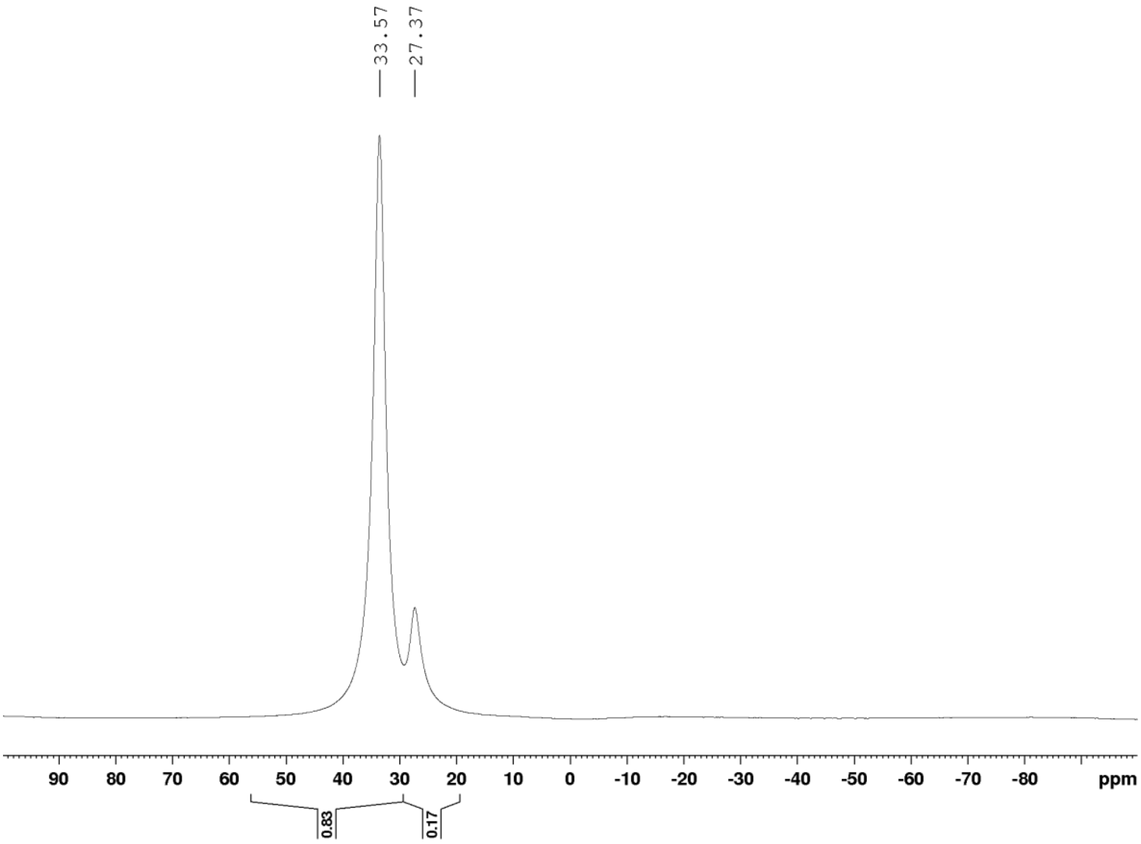

Figure S19. $^{11}B\{^{1}H\}$ of irradiated **4** in methylcyclohexane-d_{14} at 263 K.

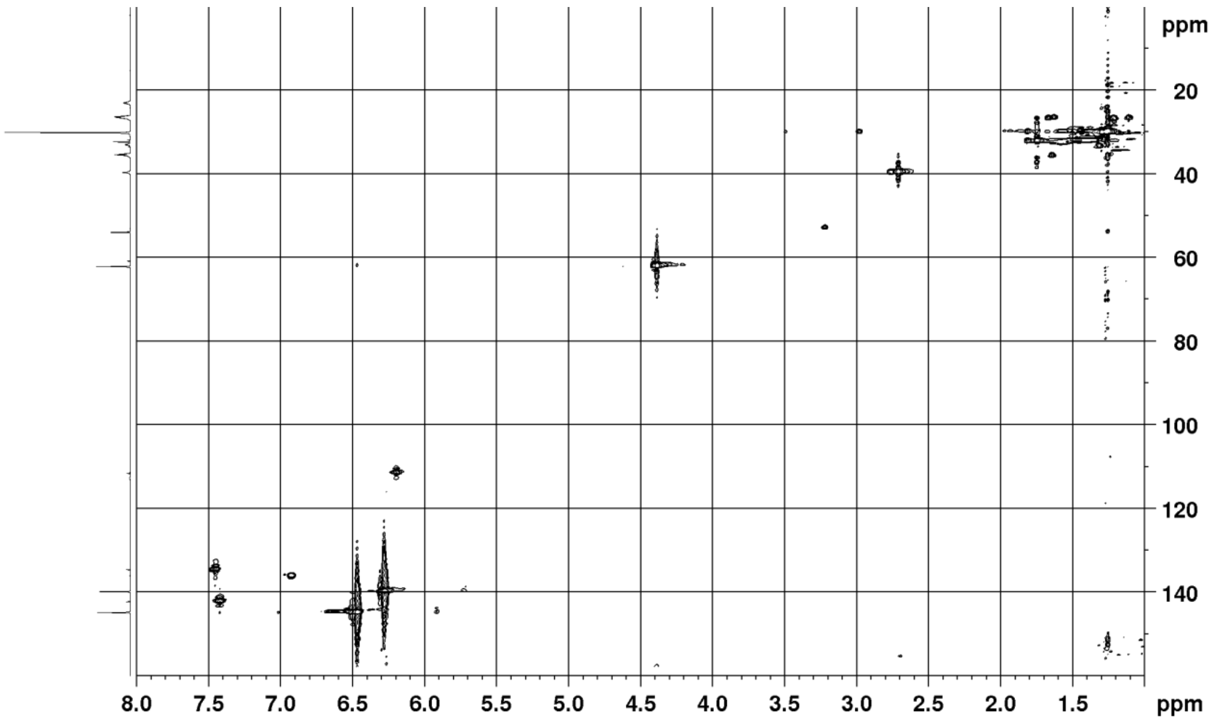

Figure S20. HSQC of irradiated **4** in methylcyclohexane-d_{14}.

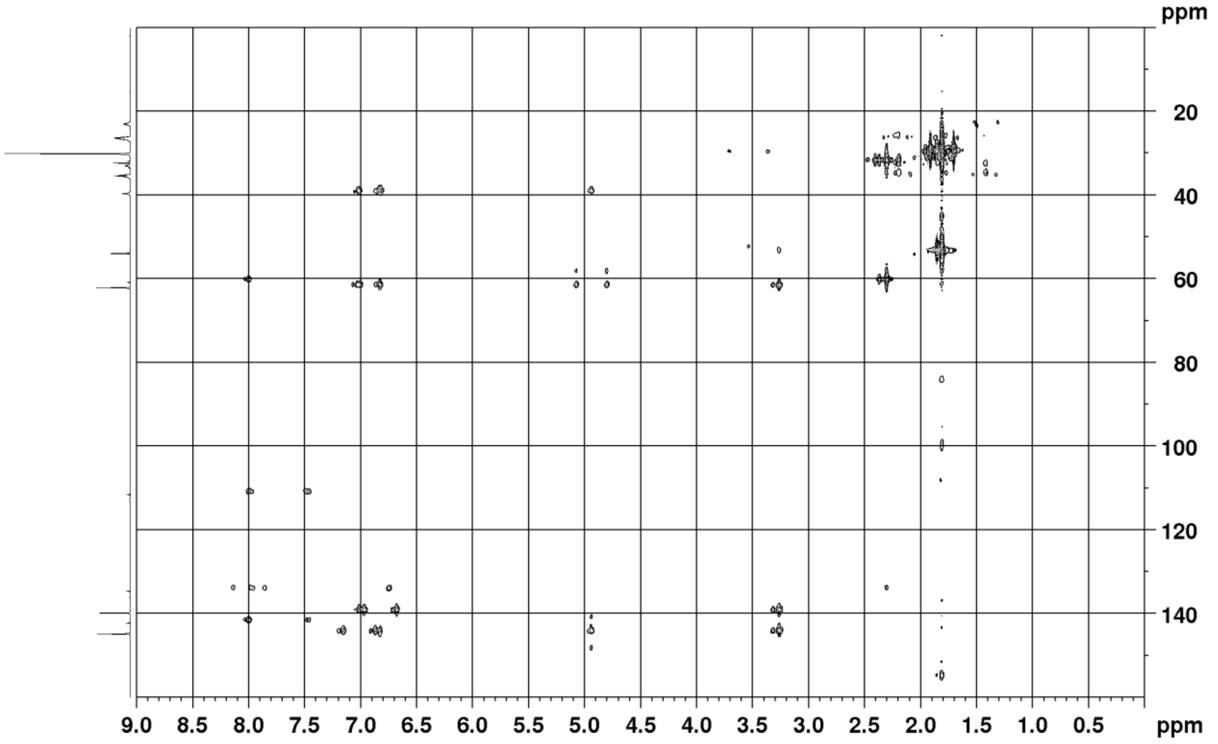

Figure S21. HMBC of irradiated **4** in methylcyclohexane-d_{14}.

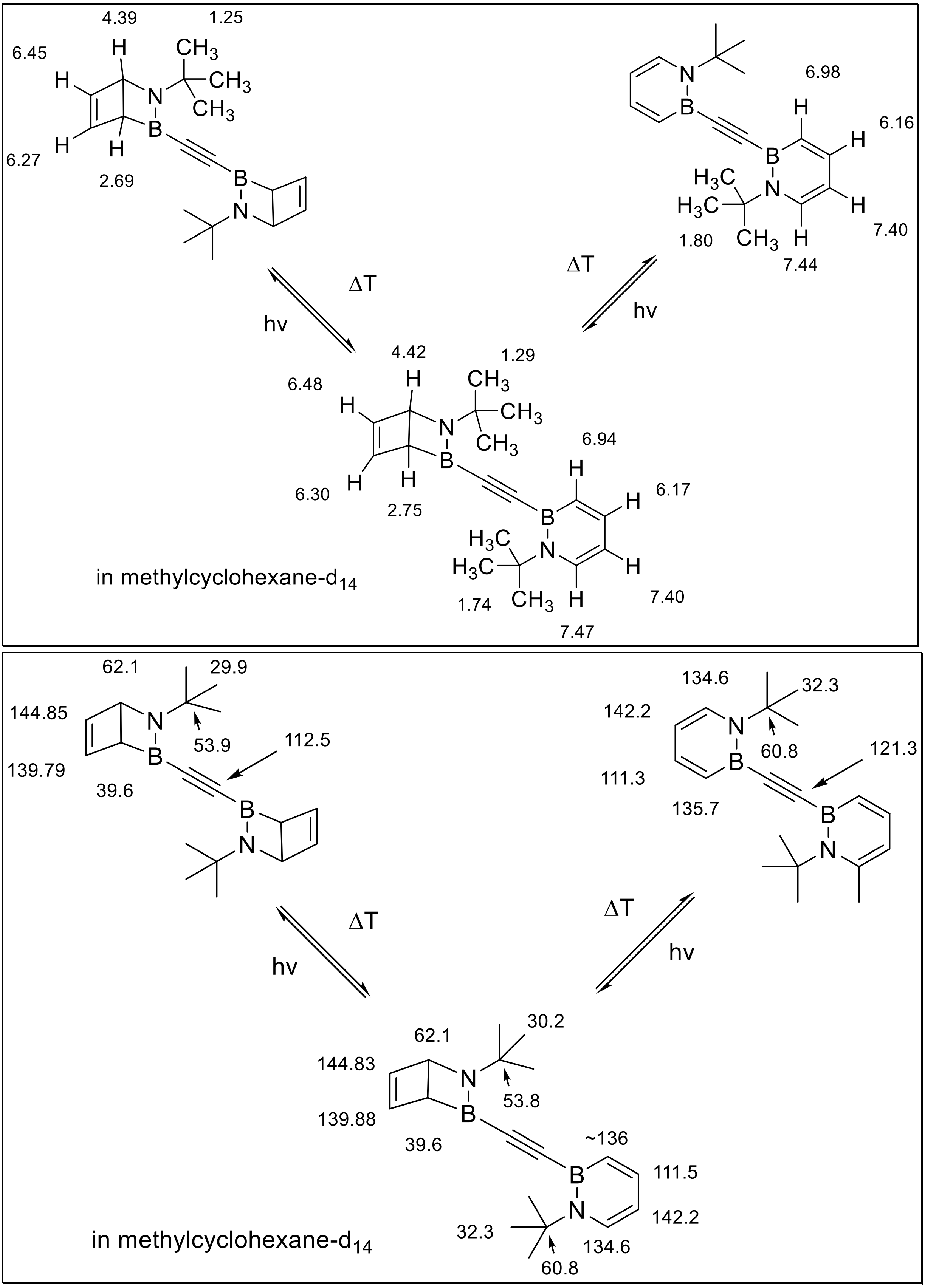

Scheme S4: Assignment of ^{1}H (top) and ^{13}C (bottom) chemical shifts for each photoisomer of **4** in methylcyclohexane-d_{14}.

6. Kinetic Data

The reaction rates were determined using ^{1}H NMR spectroscopy at four different temperatures. A 25 mM solution of dihydroazaborinine (resulting in 50 mM photoswitchable units) in deuterated cyclohexane was prepared in a quartz J. Young tube in the glovebox. The solution was irradiated for 16 minutes with wavelengths 280-400 nm from a mercury arc lamp, selected using a dichroic mirror. Rate constants were determined by analyzing the intensity of the chemical shift of the proton located on the carbon (C3) adjacent to the boron atom. The residual signal of the solvent served as a reference.

We assumed that both back reaction steps were unimolecular, with the second step occurring only after the first. Therefore, the second step depends on the concentration of the intermediate BNB-BND. These back reactions were characterized by two independent rate constants, k_1 and k_2.

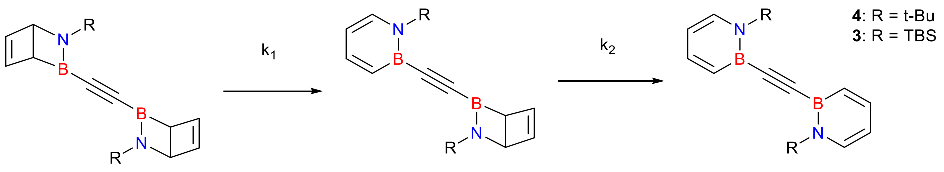

BND-BND **BNB-BND** **BNB-BNB**

The rate constants k_1 was obtained by an exponential fit:

$$a = a_0 \cdot e^{-k_1 t}$$

The rate constant k_2 was obtained by fitting the experimental data with:

$$b = k_1 \cdot a_0 \cdot \frac{e^{-k_1 t} - e^{-k_2 t}}{k_2 - k_1}$$

To determine the activation parameters, the rate constants k1 for the first reaction step **BND-BND → BNB-BND** and k_2 for the reaction **BND-BNB → BNB-BNB**, were plotted according to the Arrhenius equation (1/T vs. ln(k)), yielding the Arrhenius activation energy E_A. Subsequently, the constants were plotted according to the Eyring equation (1/T vs. ln(k/T)), which allowed us to determine the enthalpy of activation $\Delta H^‡$, entropy of activation $\Delta S^‡$, and Gibbs free energy of activation $\Delta G^‡$. All data were fitted using ORIGIN Pro 2020b Ver. 9.7.5.184.

.

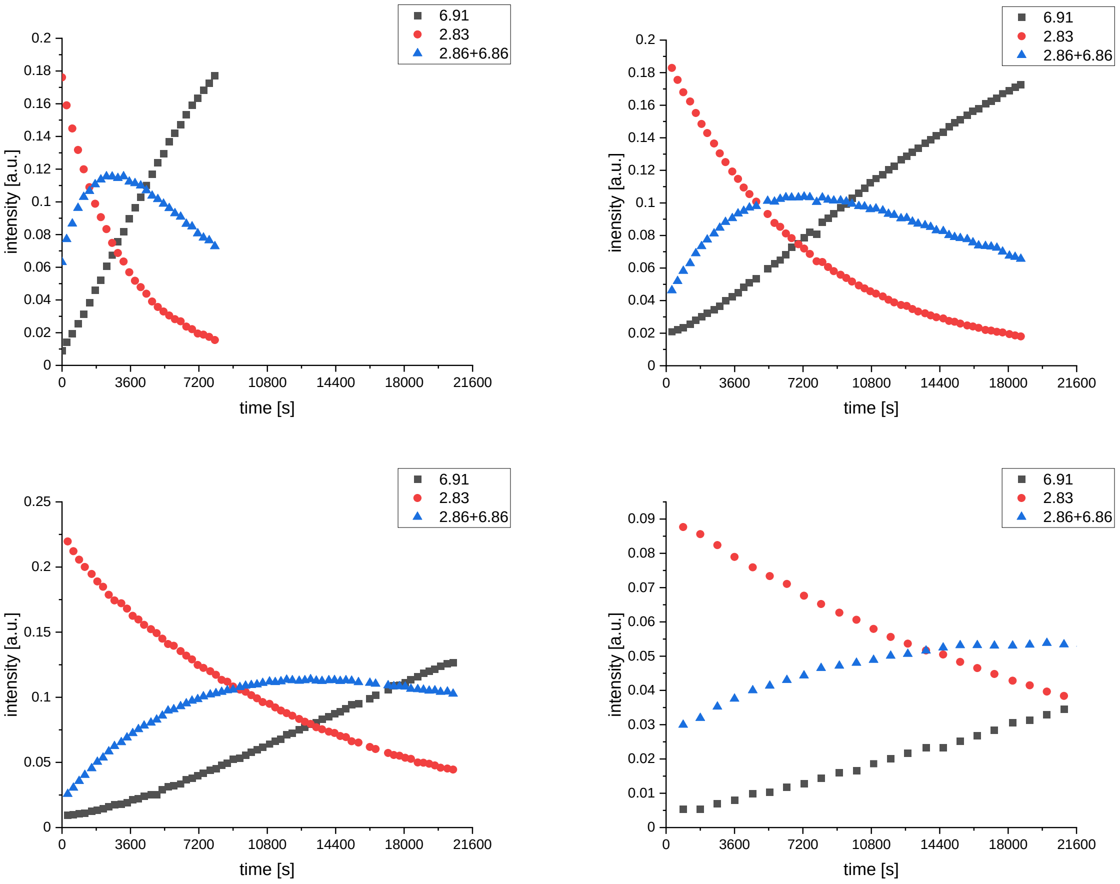

Figure S22. Kinetics of **3 BND-BND** (red), **BNB-BND** (blue) and **BNB-BNB** (black) at different Temperatures: 343 K(top left), 338 K (top right), 333 K (bottom left) 328 K (bottom right). The intensity was determined by the respective NMR chemical shift of the proton on carbon (C3) next to the boron atom.

Table S1. Rate constants for the back reactions of **3.**

Temperature	k_1 [1/s] **BND-BND** $\rightarrow$ **BNB-BND**	k_2 [1/s] **BNB-BND** $\rightarrow$ **BNB-BNB**
343 K	3.093 10^{-4}	1.927 10^{-4}
338 K	1.316 10^{-4}	9.971 10^{-5}
333 K	7.809 10^{-5}	5.357 10^{-5}
328 K	4.177 10^{-5}	2.708 10^{-5}

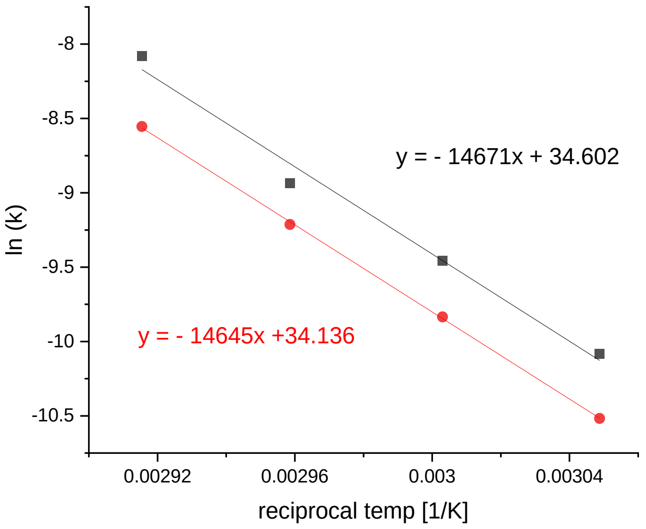

Figure S23. Arrhenius plot of **3** for k_1 (black) and k_2 (red) giving the values E_{A1} = 120 ± 10 kJ/mol and E_{A2} = 120 ± 1 kJ/mol.

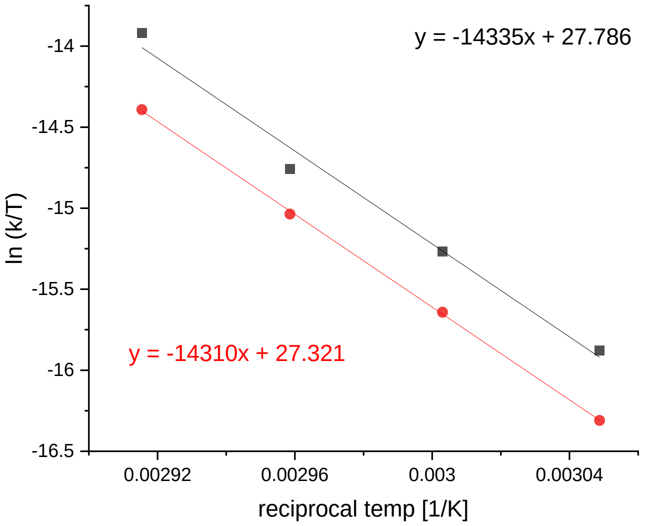

Figure S24. Eyring plot of **3** for k_1 (black) and k_2 (red) giving the values

	$\Delta H^{\ddagger}$ [kJ/mol]	$\Delta S^{\ddagger}$ [kJ/(J*K)]	$\Delta G^{\ddagger}$ [kJ/mol]
BND-BND → BNB-BND	119 ± 10	33.5 ± 4.2	109 ± 11
BNB-BND → BNB-BNB	119 ± 1	29.6 ± 0.5	110 ± 2

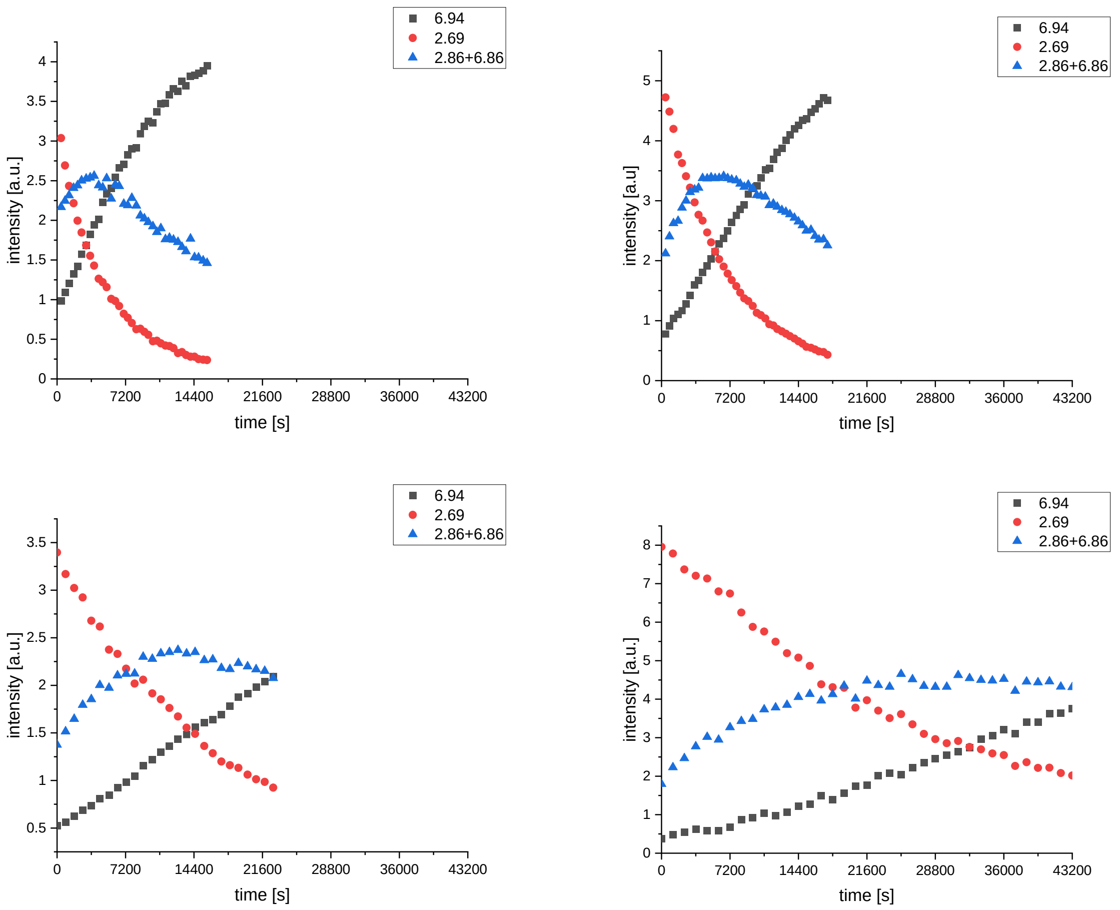

Figure S25. Kinetics of **4 BND-BND** (red), **BNB-BND** (blue) and **BNB-BNB** (black)) at different Temperatures: 348 K(top left), 343 K (top right), 338 K (bottom left) 333 K (bottom right). The intensity was determined by the respective NMR chemical shift of the proton on carbon (C3) next to the boron atom.

Table S2. Rate constants for the back reactions of **4**.

Temperature	k_1 [1/s] **BND-BND → BNB-BND**	k_2 [1/s] **BNB-BND → BNB-BNB**
348 K	1.856 10^{-4}	1.243 10^{-4}
343 K	1.451 10^{-4}	9.136 10^{-5}
338 K	5.744 10^{-5}	4.015 10^{-5}
333 K	3.349 10^{-5}	2.422 10^{-5}

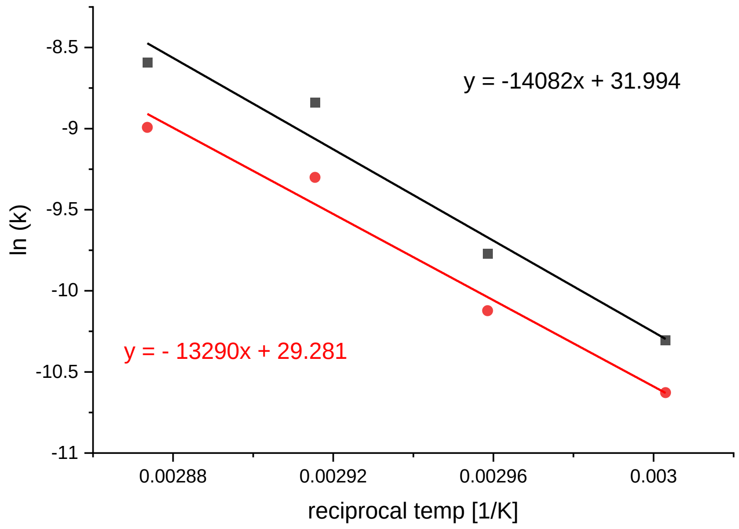

Figure S26. Arrhenius plot for **4** k_1 (black) and k_2 (red) giving the values E_{A1} = 115 ± 16 kJ/mol and E_{A2} = 109 ± 12 kJ/mol.

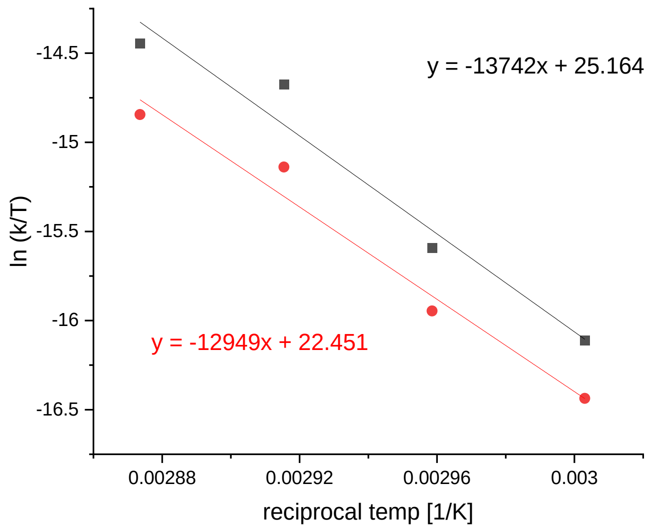

Figure S27. Eyring plot for **4** for k_1 (black) and k_2 (red) giving the values

	$\Delta H^‡$ [kJ/mol]	$\Delta S^‡$ [kJ/(J*K)]	$\Delta G^‡$ [kJ/mol]
BND-BND → BNB-BND	111 ± 17	11.7 ± 2.7	109 ± 18
BNB-BND → BNB-BNB	108 ± 12	-10.9 ± 2.1	111 ± 12

7. **Heat Release (DSC)**

To exclude any contribution from the solvent, a DSC measurement of the solvent only vs. empty crucible was performed. This control experiment showed no exothermic or endothermic events in the relevant temperature range, confirming that the observed heat release can be attributed solely to the **BND-BND** to **BNB-BNB** conversion (Figure S28).

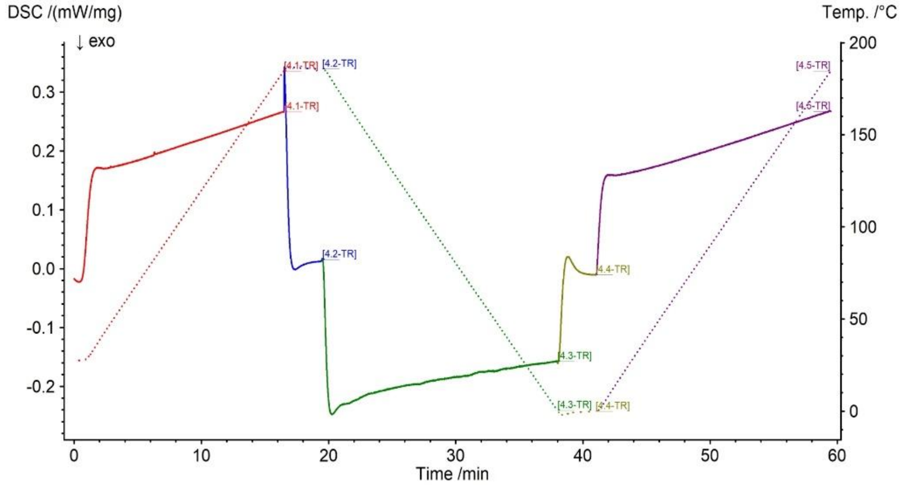

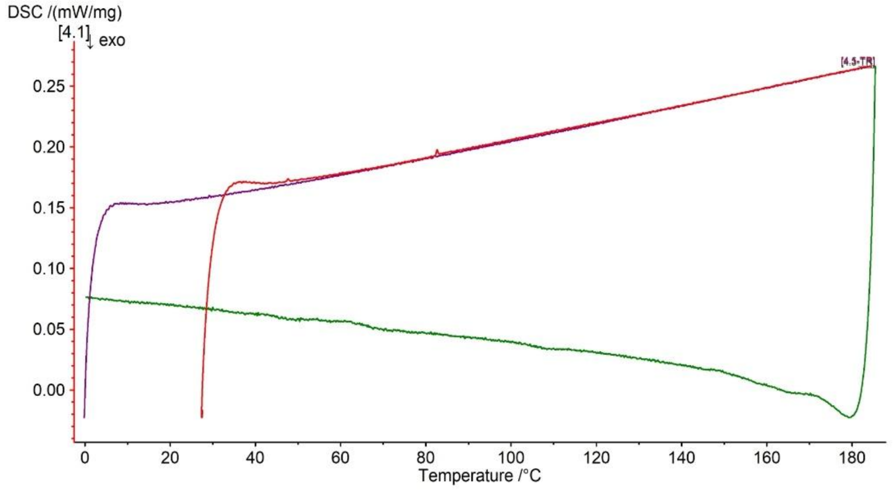

Figure S28. [top] DSC thermogram of 30 μL decalin including heating/cooling profile and [bottom] detailed comparison of the first (red), cooling (green) and the second heating cycle (purple).

Additionally, to further minimize thermal artifacts caused by the solvent, all DSC samples were measured against a reference crucible containing 30 μL of the same solvent. This ensured that any thermal effects observed are due to the sample itself and not due to solvent evaporation or baseline shifts.

As the temperature range of decalin was not sufficiently wide for the investigation of the **2BND to 2BNB** back reaction, dodecane was used as solvent instead. To exclude any contribution from the solvent, a DSC measurement of the solvent only sample vs. empty crucible was performed (Figure S29).

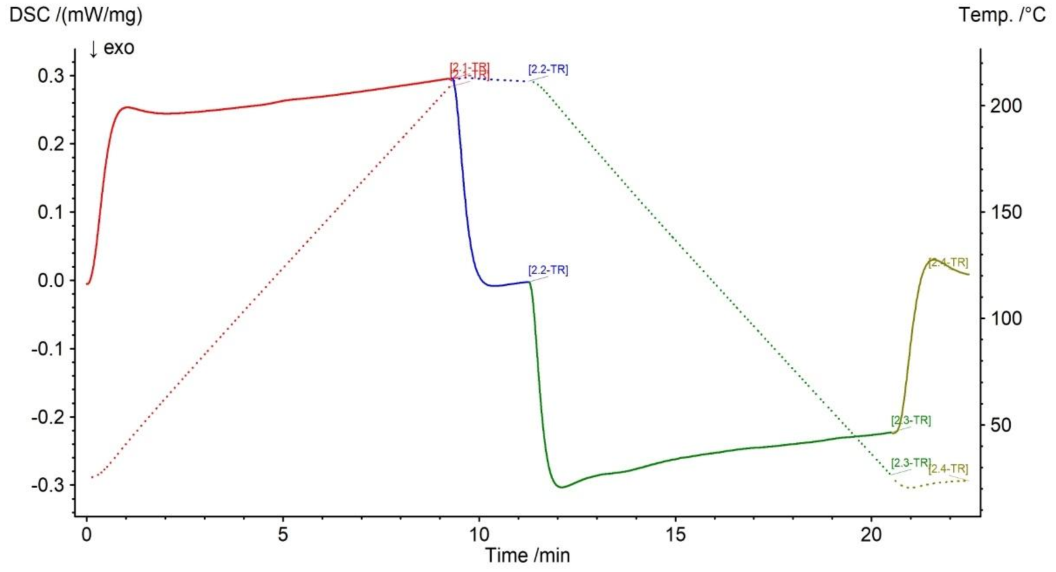

Figure S29. DSC thermogram of 30µL dodecane including heating/cooling profile.

7.1 Sample preparation for DSC

All samples for DSC were prepared in a glovebox. Weight of the samples and crucibles was determined by using a fine scale, with an accuracy of 0.01 mg.

Samples of compounds **1** (18.3 mg), **3** (7.0 mg), and **4** (12.2 mg) were each dissolved in 300 µL of non-deuterated, degassed decalin using an Eppendorf pipette. Compound **2** (15.7 mg) was dissolved in the same way in 300 µL of non-deuterated dodecane. The resulting solutions were transferred to quartz J. Young NMR tubes.

A ^{11}B NMR spectrum was recorded before irradiation as a reference, without *"solvent lock"*. The samples were then irradiated with 280-400 nm light from a mercury arc lamp, using a dichroic mirror to select the desired wavelength range. The conversion rate was monitored by ^{11}B NMR until no further change in the spectra was observed.

The tubes were returned to the glovebox. Each solution was divided in two portions. For **DSC measurements**, 30 µL of each solution was transferred to pre-weighed aluminum crucibles, which were sealed with lids inside the glovebox and weighed again. Post-DSC, the crucibles were reweighed to confirm they remained sealed.

The remaining part of the solutions was used for a more precise determination of the conversion ratio. To each remaining portion, 400 µL of deuterated dichloromethane was added, and a $^{\mathbf{1}}$H NMR spectrum was recorded. Since the spectral region of interest is not affected by the excess of decalin oder dodecane, the conversion ratio was determined by comparing the signals of the protons adjacent to boron or nitrogen in the switched and unswitched **BNB** isomers (Illustration of this workflow in Figure S30).

7.2 Temperature Program for DSC

For every compund **1-4** an individual temperature programme was set up. All of them follow the same pattern. In a first step the samples were heated up to a temperature where no recordable backconversion occurred yet. This temperature was extrapolated from the kinetic data obtained and described in SI section 6. After holding this temperature for five minutes the samples were

heated up (solid lines in the thermogramms) close to the boiling point of the utilzed solvent. The final temperature was held for additional five minutes to ensure that the backreaction is completed and no further heat release occured. The samples were cooled in the calorimeter again to the starting temperature and the procedure was repeated without any manipulation of the sampes (dashed lines in the thermogramms) to demonstrate that the conversion to the BNB was completed and no furter reactions occur.

7.3 Backconversion Check by NMR

To confirm complete backconversion and ensure the dihydroazaborinines remained intact after DSC measurements, all crucibles of a given compound were opened and extracted with deuterated dichloromethane. The extracts were combined, transferred to a quartz NMR tube, and analyzed by both ^{1}H and ^{11}B NMR spectroscopy.

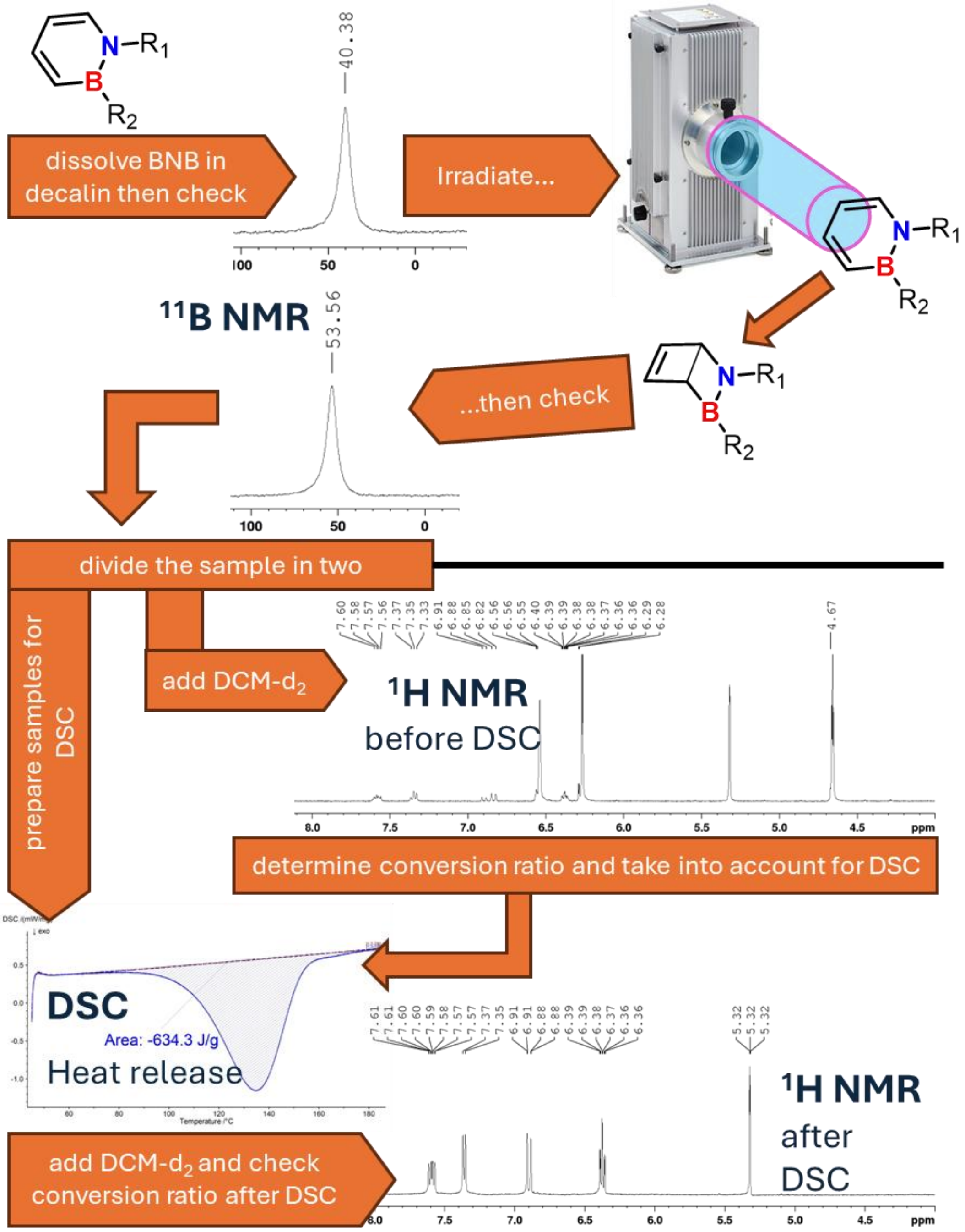

Figure S30. Illustration of the heat release determination workflow.

7.4 Thermal analysis for 1BNB (no solvent):

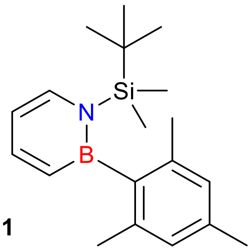

1

Sample

**Sample Name**	**1BNB**
**Sample Mass:**	4.21 mg

Graphic

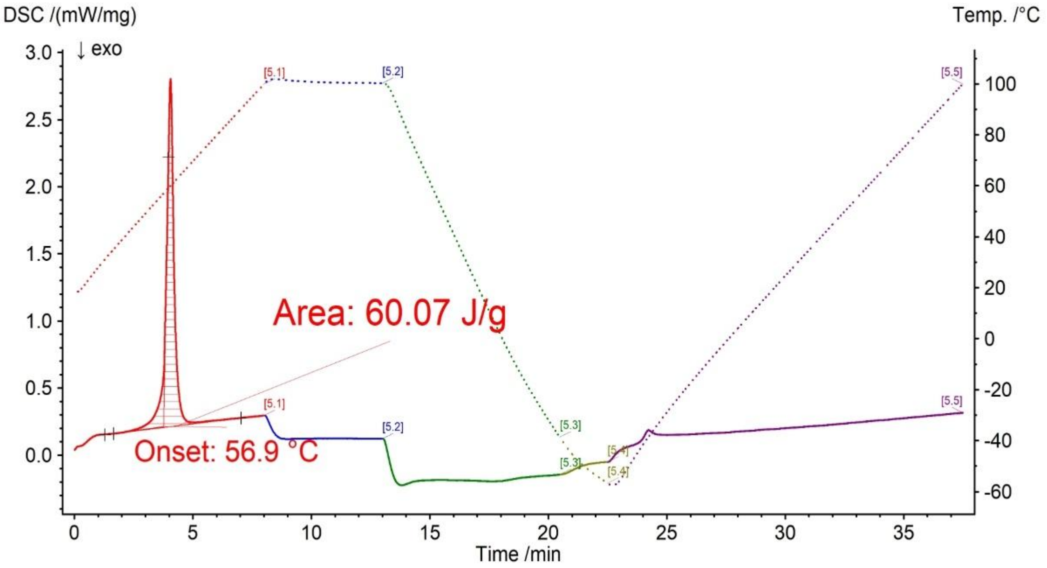

Results

Parameters	Result	Range (min)	Range (max)
**Onset (DSC)**	56.9 °C	1.280 min	6.718 min
**Area (DSC),o**	60.07 J/g	1.650 min	7.040 min

Figure S31. Thermogram of **1BNB** without solvent.

7.5 Thermal analysis for “oily” 1BND (no solvent):

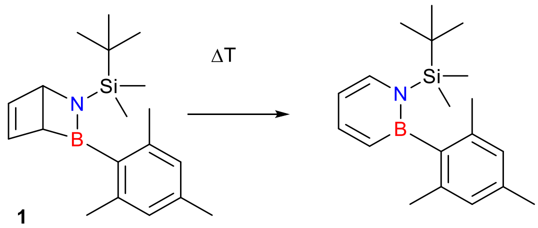

Sample

**Sample name:**	**1BND**
**Sample Mass:**	8.35 mg

Graphic

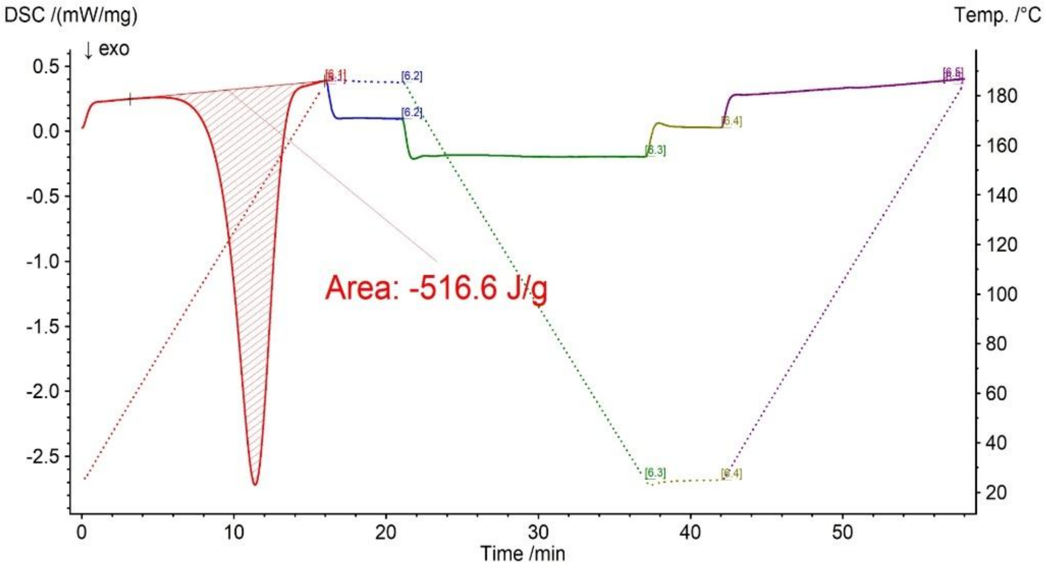

Results

Parameters	Result	Range (min)	Range (max)
**Area (DSC),o**	-516.6 J/g	3.190 min	15.960 min

Figure S32. Thermogram of **1BND** without solvent.

7.6 Thermal analysis of irradiated 1 in solution:

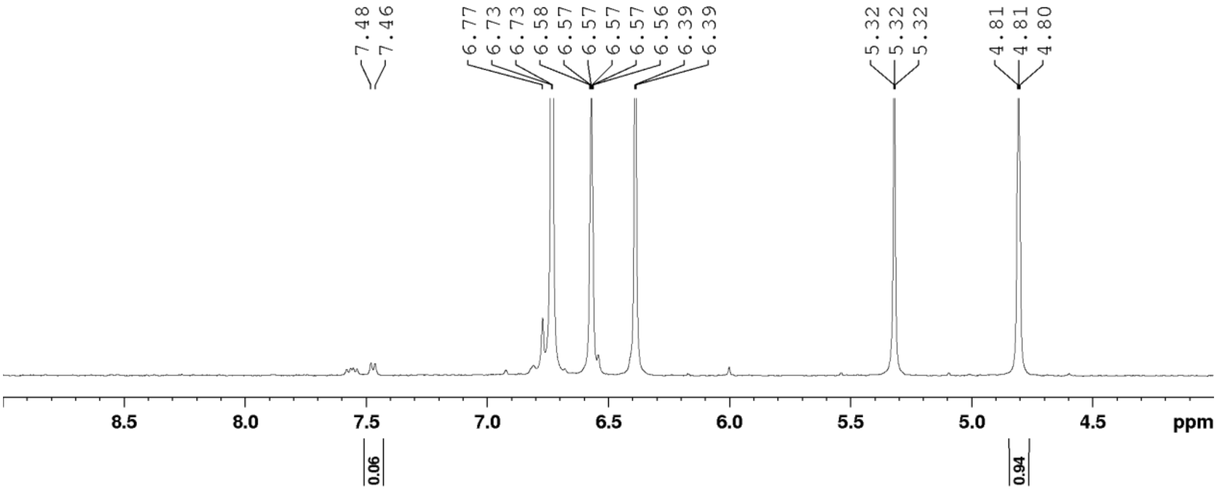

Figure S33. ^{1}H NMR spectra of **1BNB** and **1BND** in decalin/dichloromethane-d_2 after photoconversion for determination of conversion ratio and before DSC heat release The conversion ratio of **BNB** into **BND** units 94%.

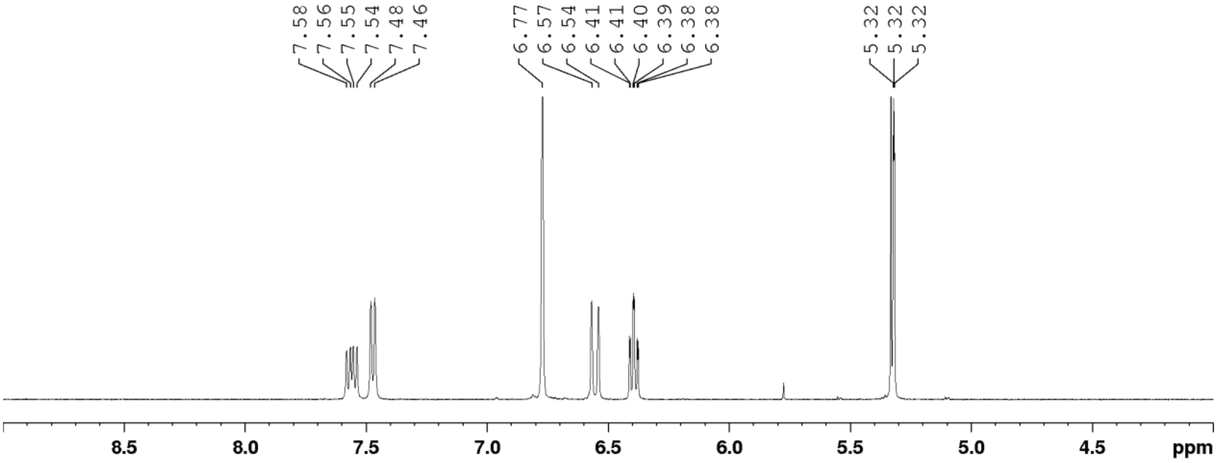

Figure S34. ^{1}H NMR spectra of all the combined DSC samples in decalin/dichloromethane-d_2 after heat release showing only **1**.

Temperature program and thermograms for **1BND** in solution

A

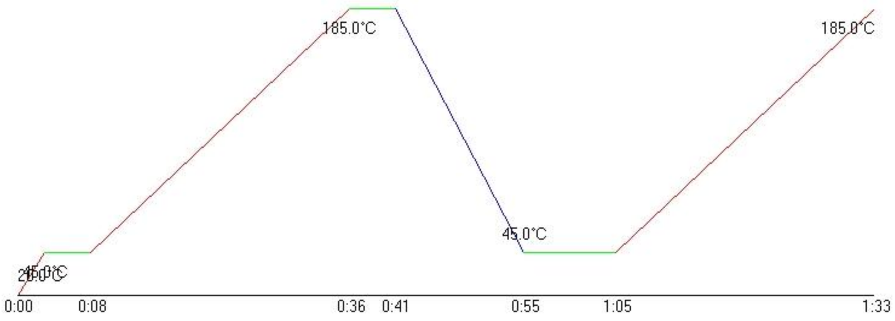

Temperature Steps

Num	Mode	Temp. °C	HR K/min	Acq.Rate pts/min	Duration hh:mm
---	Start	20.0			
1	Dynamic	45.0	10.000	300.00	00:03
2	Isothermal	45.0		25.00	00:05
3	Dynamic	185.0	5.000	300.00	00:28
4	Isothermal	185.0		50.00	00:05
5	Dynamic	45.0	10.000	600.00	00:14
6	Isothermal	45.0		25.00	00:10
7	Dynamic	185.0	5.000	300.00	00:28
---	Emergency	195.0			

Sample1

Sample Mass: 1.641 mg

B

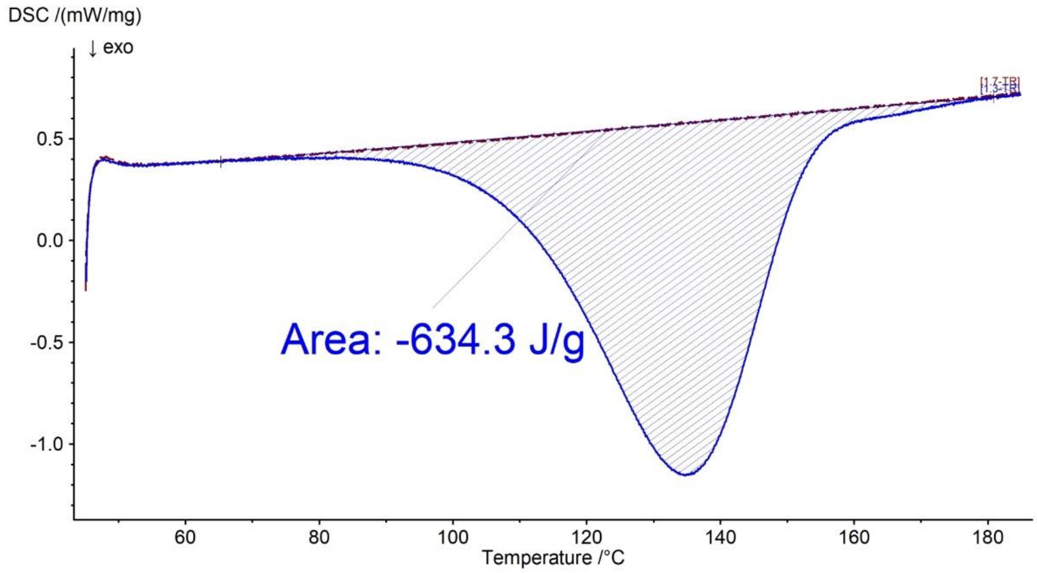

Parameters	Result	Range (min)	Range (max)
**Area (DSC),o**	-634.3 J/g	65.3 °C	180.9 °C
**Corrected heat release**	**-674,8 J/g**		

Sample2

Sample Mass: 1.699 mg

C

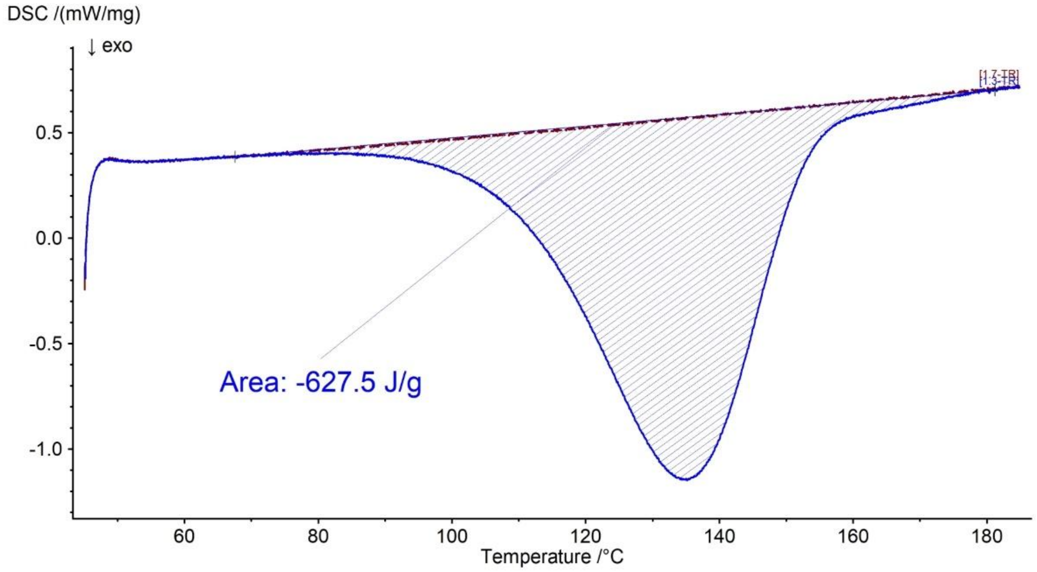

Parameters	Result	Range (min)	Range (max)
**Area (DSC),o**	-627.5 J/g	67.6 °C	181.2 °C
**Corrected heat release**	**-667,6 J/g**		

Sample3

Sample Mass: 1.667 mg

D

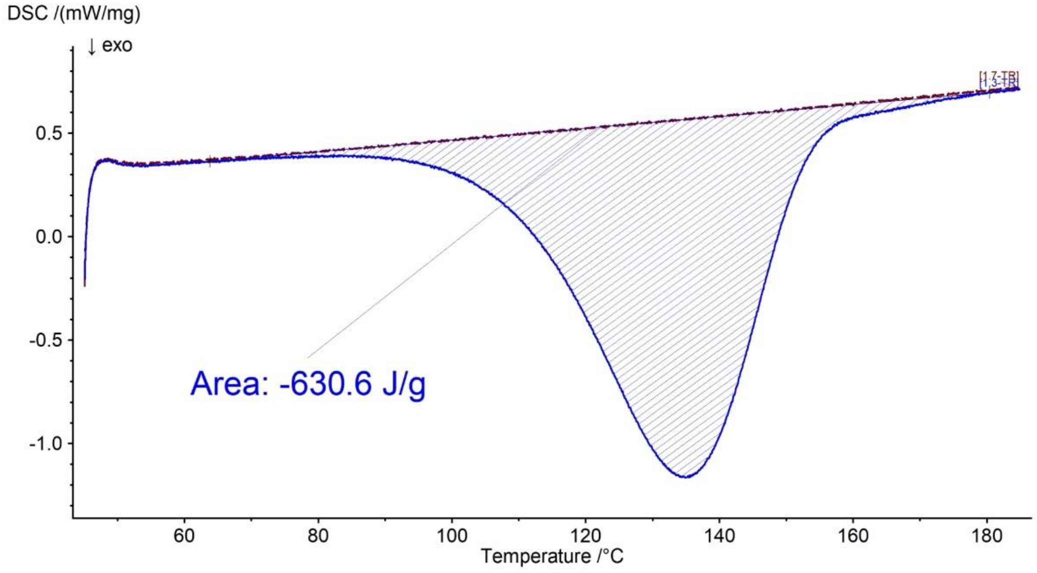

Parameters	Result	Range (min)	Range (max)
**Area (DSC),o**	-630.6 J/g	63.8 °C	180.4 °C
**Corrected heat release**	**-670,9 J/g**		

Figure S35. Temperature program [A] and thermograms [B-D] of **1BND** in decalin.

7.7 Thermal analysis for 2BND (no solvent):

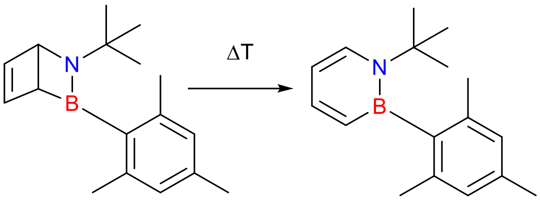

**Sample name:**	**2BND**
**Sample Mass:**	2.9 mg

Graphic

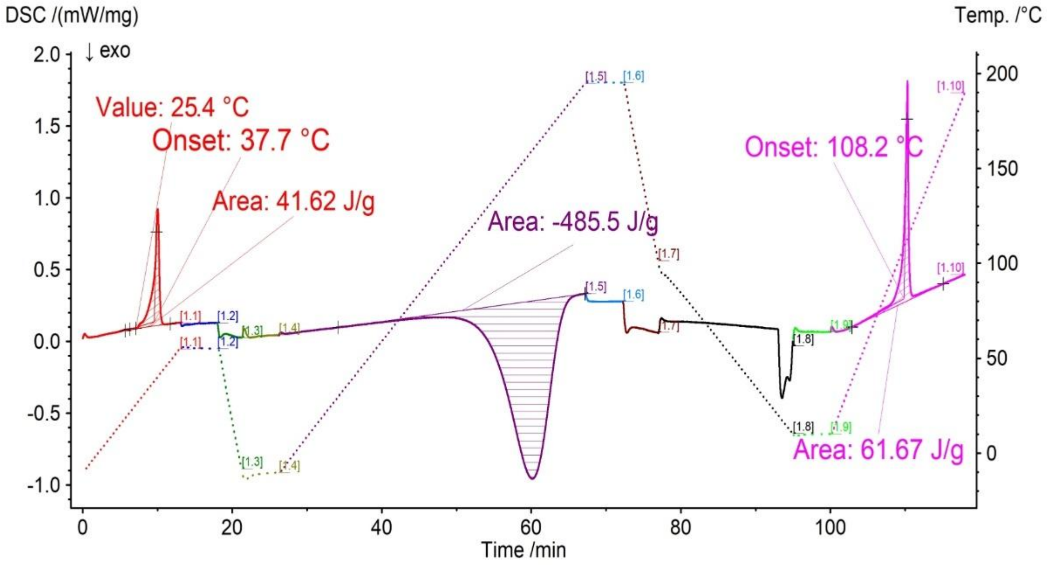

Results

Parameters	Result	Range (min)	Range (max)
**Onset (DSC)**	37.7 °C	5.700 min	12.100 min
**Area (DSC),o**	41.62 J/g	6.300 min	11.700 min
**Value (DSC)**	0.08902 mW/mg	7.100 min	
**Area (DSC),o**	-485.5 J/g	34.200 min	67.200 min
**Area (DSC),o**	61.67 J/g	102.900 min	115.100 min
**Onset (DSC)**	108.2 °C	102.900 min	115.118 min

Figure S36. Thermogram of **2BND** without solvent.

7.8 Thermal analysis for solid 2BNB:

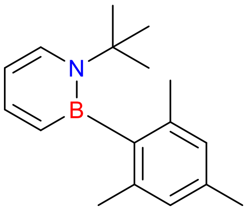

Sample

**Sample name:**	**2BNB**
**Sample Mass:**	6.15 mg

Graphic

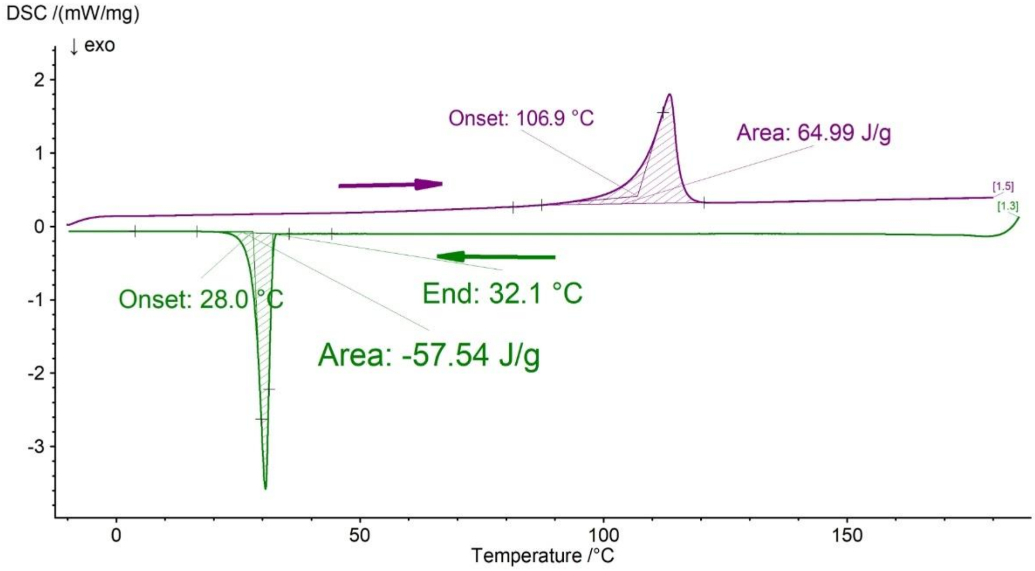

Results

Parameters	Result	Range (min)	Range (max)
**Onset (DSC)**	28.0 °C	3.8 °C	50.0 °C
**Area (DSC),o**	-57.54 J/g	16.5 °C	35.5 °C
**End (DSC)**	32.1 °C	21.4 °C	44.2 °C
**Onset (DSC)**	106.9 °C	81.5 °C	146.1 °C
**Area (DSC),o**	64.99 J/g	87.3 °C	120.6 °C

Figure S37. Thermogram of **2BNB** without solvent.

7.9 Thermal analysis of irradiated 2 in solution:

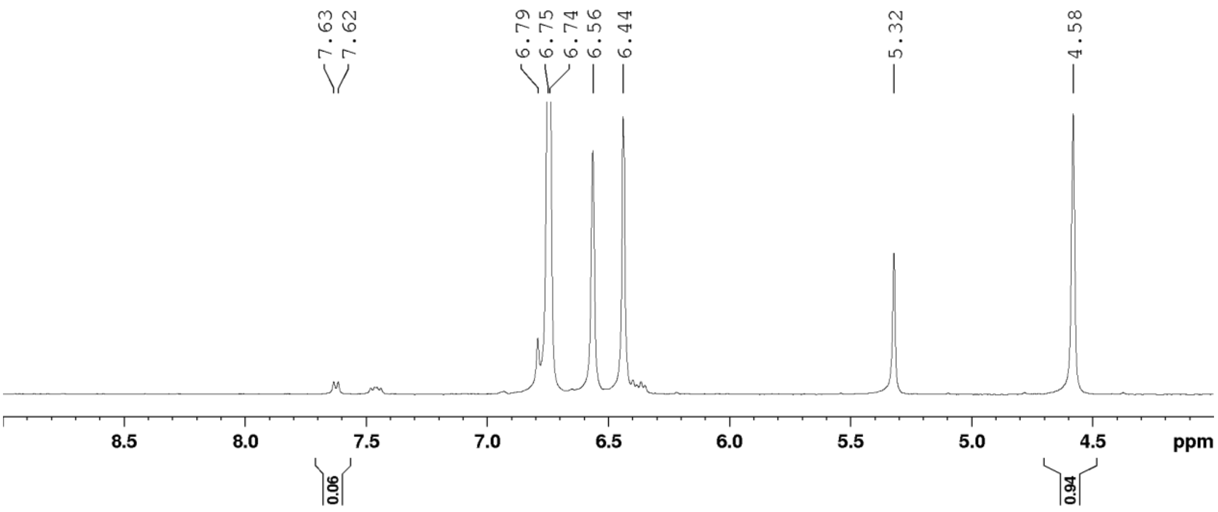

Figure S38. ^{1}H NMR spectra of **2BNB** and **2BND** in dodecane/dichloromethane-d_2 after photoconversion for determination of conversion ratio and before DSC heat release. The conversion ratio of **BNB** into **BND** units is 94%.

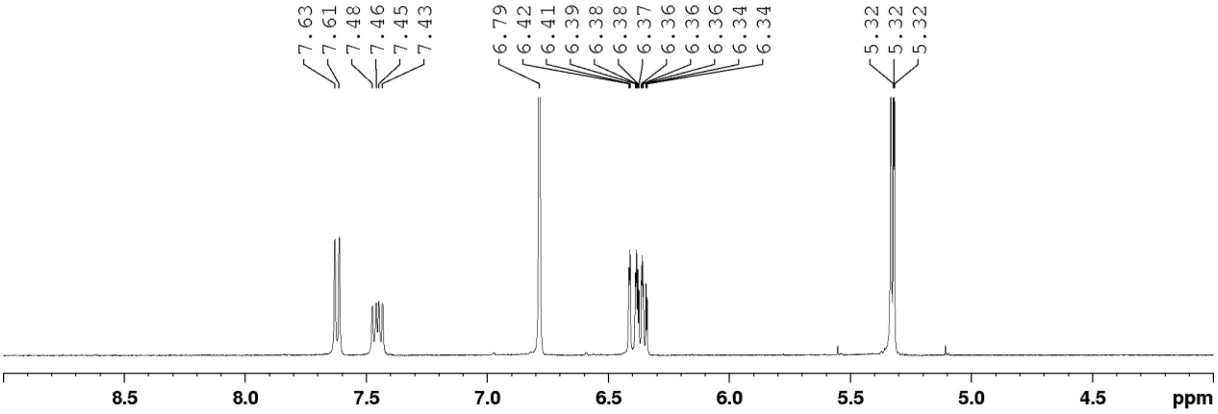

Figure S39. ^{1}H NMR spectra of all the combined DSC samples in dodecane/dichloromethane-d_2 after heat release showing only **2**.

Temperature program and thermograms for **2BND** in solution

A

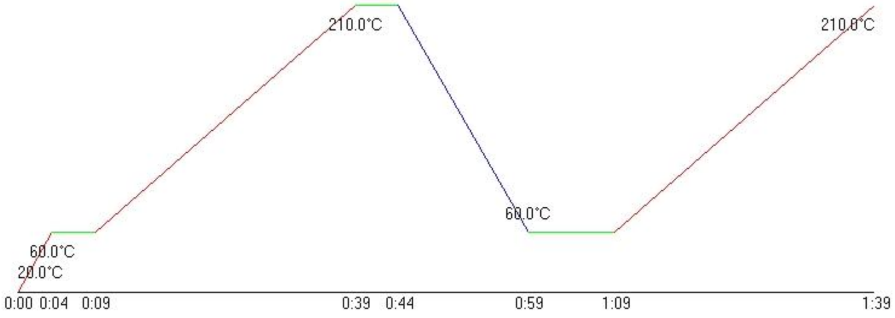

Temperature Steps

Num	Mode	Temp. °C	HR K/min	Acq.Rate pts/min	Duration hh:mm
---	Start	20.0			
**1**	Dynamic	60.0	10.000	300.00	00:04
**2**	Isothermal	60.0		25.00	00:05
**3**	Dynamic	210.0	5.000	300.00	00:30
**4**	Isothermal	210.0		50.00	00:05
**5**	Dynamic	60.0	10.000	600.00	00:15
**6**	Isothermal	60.0		25.00	00:10
**7**	Dynamic	210.0	5.000	300.00	00:30
---	Emergency	220.0			

**Sample1**	
**Sample Mass:**	1.497 mg

B

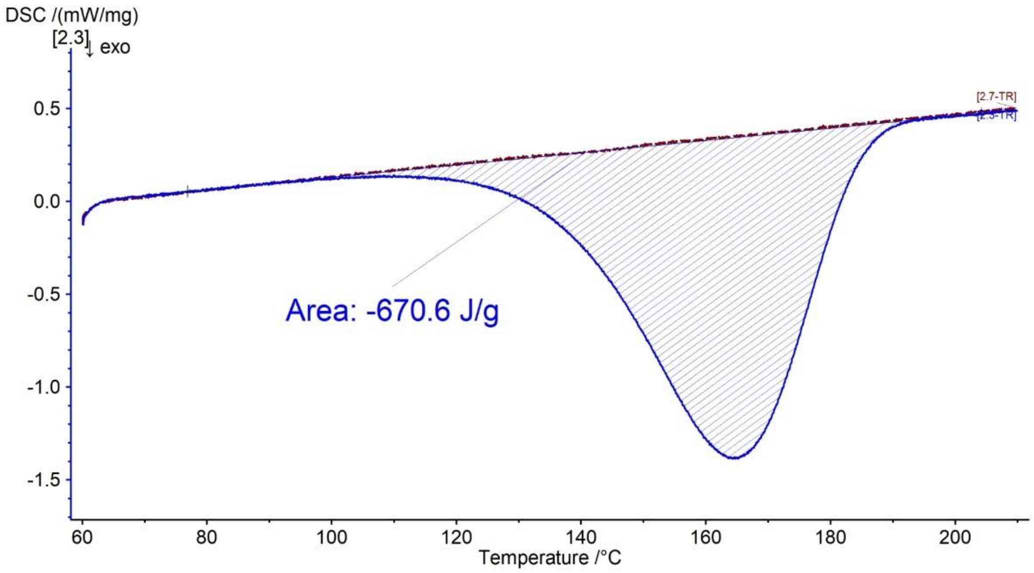

Parameters	Result	Range (min)	Range (max)
**Area (DSC),o**	-670.6 J/g	76.9 °C	204.2 °C
**Corrected heat release**	-713.4 J/g		

Sample 2

Sample Mass: 1.415 mg

C

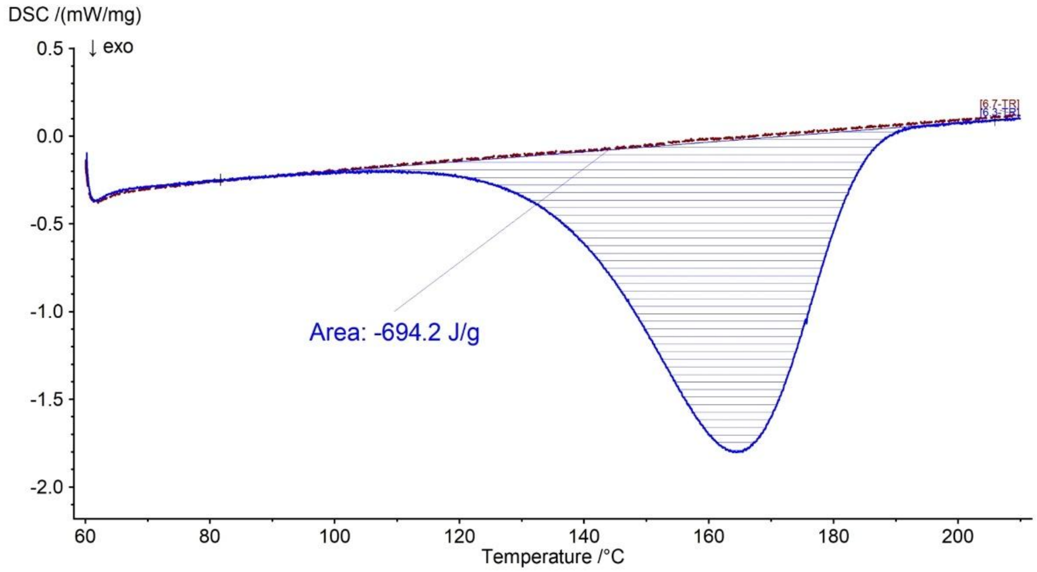

Parameters	Result	Range (min)	Range (max)
**Area (DSC),o**	-694.2 J/g	81.7 °C	205.9 °C
**Corrected heat release**	-735.1 J/g		

Sample3

Sample Mass: 0.917 mg

D

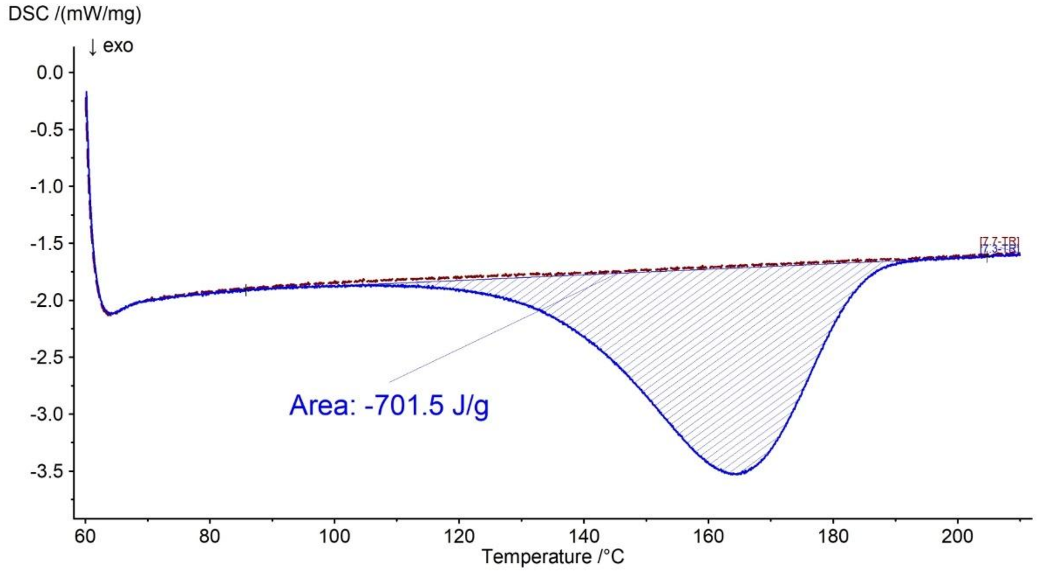

Parameters	Result	Range (min)	Range (max)
**Area (DSC),o**	-701.5 J/g	85.8 °C	204.7 °C
**Corrected heat release**	-746.3 J/g		

Figure S40. Temperature program [A] and thermograms [B-D] of 2**BND** in dodecane.

7.10 Thermal analysis for irradiated "oily" 3 (no solvent):

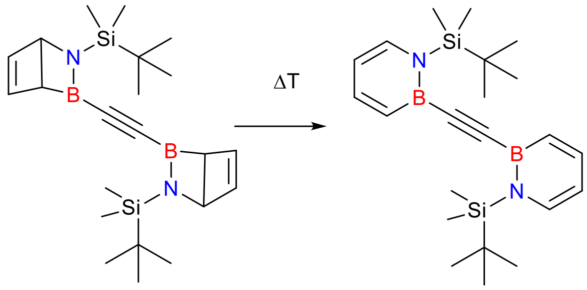

Sample

**Sample name:**	**3 neat**
**Sample Mass:**	4.18 mg

Graphic

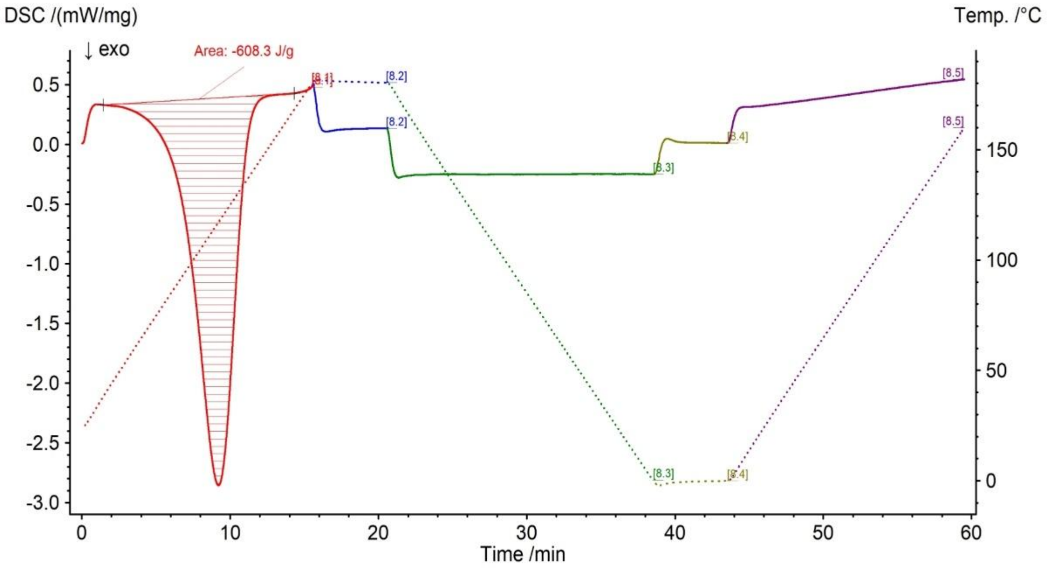

Results

Parameters	Result	Range (min)	Range (max)
**Area (DSC),o**	-608.3 J/g	1.440 min	14.330 min

Figure S41. Thermogram of **3BNB-BNB** without solvent.

7.11 Thermal analysis of irradiated 3 in solution:

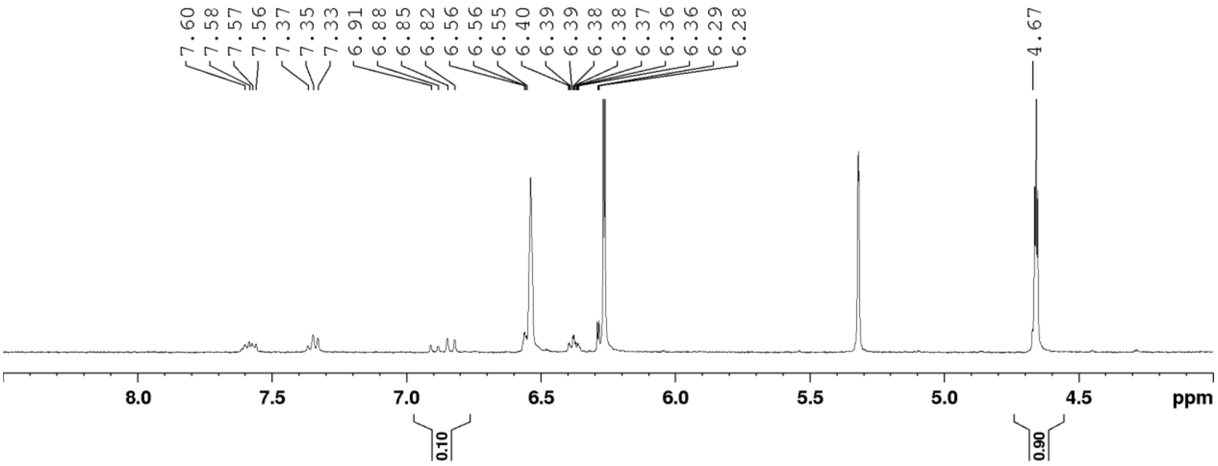

Figure S42. ^{1}H NMR spectra of **3BNB-BNB** and **3BND-BNB** and **3BNB-BNB** in decalin/dichloromethane-d_2 after photoconversion for determination of conversion ratio and before DSC heat release. The conversion ratio of **BNB** into **BND** units is 90% overall.

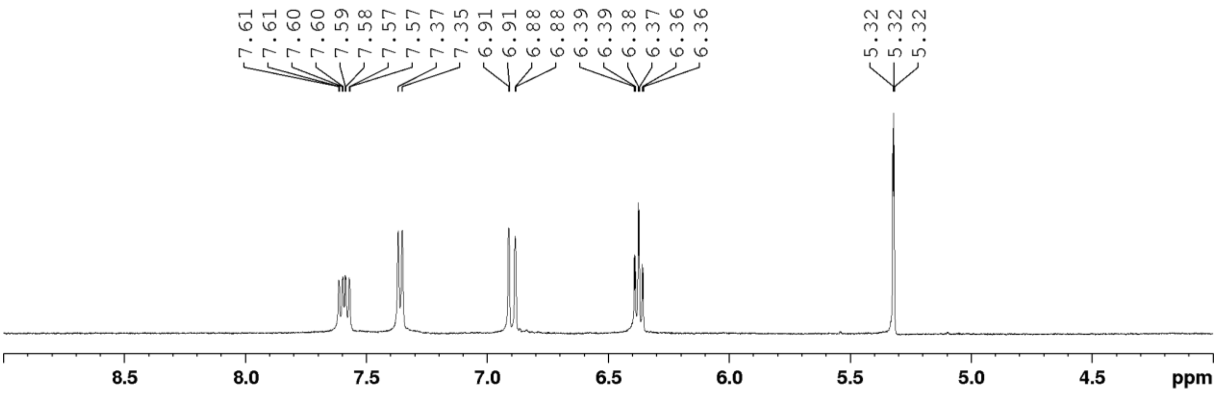

Figure S43. ^{1}H NMR spectra of all the combined DSC samples in decalin/dichloromethane-d_2 after heat release showing only **3** .

Temperature program and thermograms for **3**

A

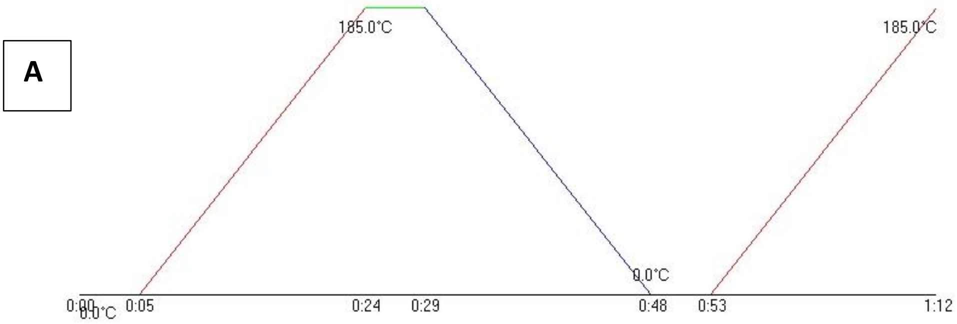

Temperature Steps

Num	Mode	Temp. °C	HR K/min	Acq.Rate pts/min	Duration hh:mm
---	Start	0.0			
1	Isothermal	0.0		50.00	00:05
2	Dynamic	185.0	10.000	150.00	00:19
3	Isothermal	185.0		50.00	00:05
4	Dynamic	0.0	10.000	300.00	00:19
5	Isothermal	0.0		50.00	00:05
6	Dynamic	185.0	10.000	150.00	00:19
---	Emergency	195.0			

Sample1

Sample Mass: 0.762 mg

B

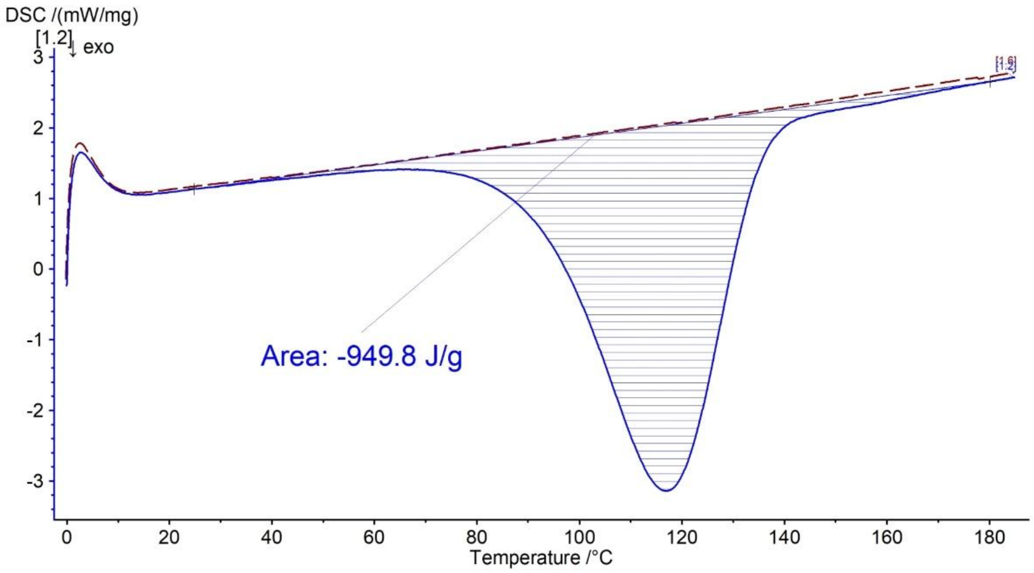

Parameters	Result	Range (min)	Range (max)
**Area (DSC),o**	-949.8 J/g	24.8 °C	180.2 °C
**Corrected heat release**	-1055.3 J/g		

Sample2

Sample Mass: 0.777 mg

C

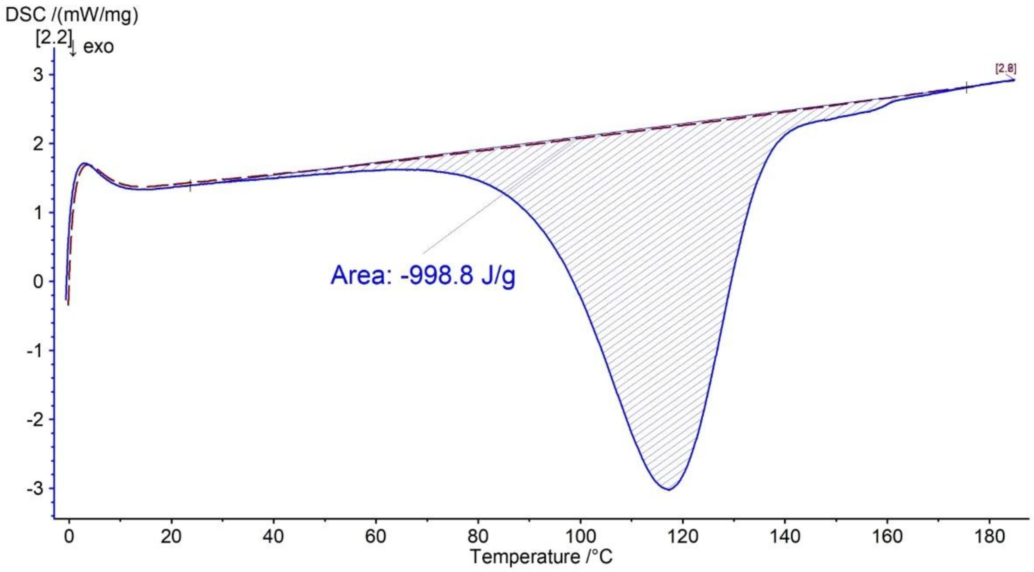

Parameters	Result	Range (min)	Range (max)
**Area (DSC),o**	-998.8 J/g	23.7 °C	175.6 °C
**Corrected heat release**	-1109.8		

Sample3

Sample Mass: 0.765 mg

D

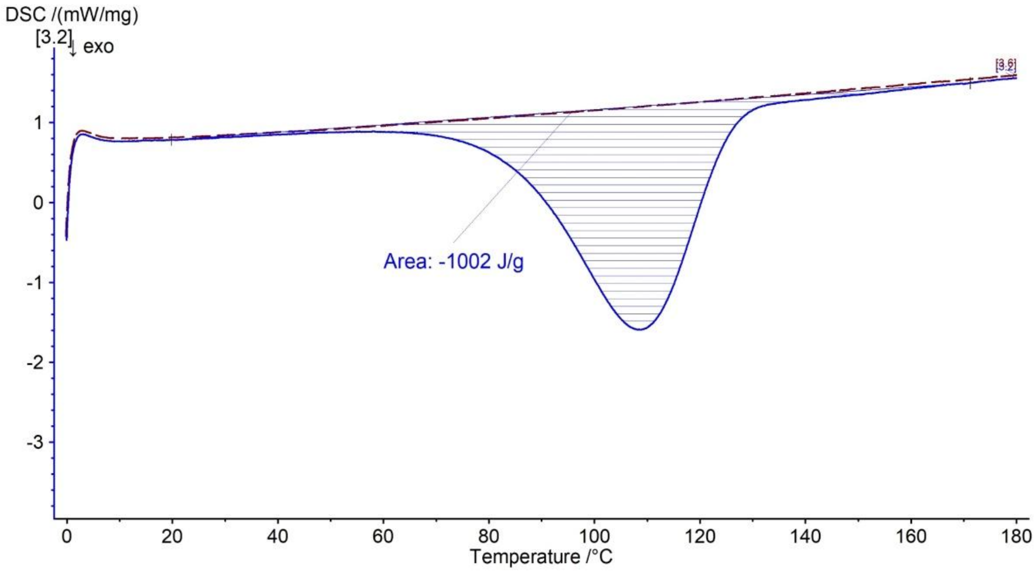

Parameters	Result	Range (min)	Range (max)
**Area (DSC),o**	-1002 J/g	19.9 °C	171.3 °C
**Corrected heat release**	-1113.3		

Figure S44. Temperature program [A] and thermograms [B-D] of **3BND-BND** in decalin.

7.12 Thermal analysis for "oily" 4 (no solvent):

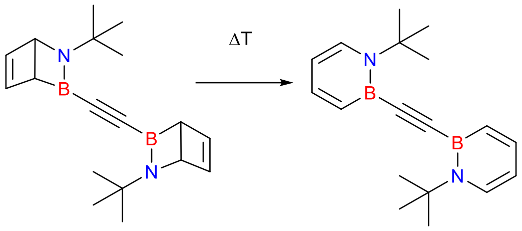

Sample

**Sample name:**	**4 neat**
**Sample Mass:**	4.48 mg

Graphic

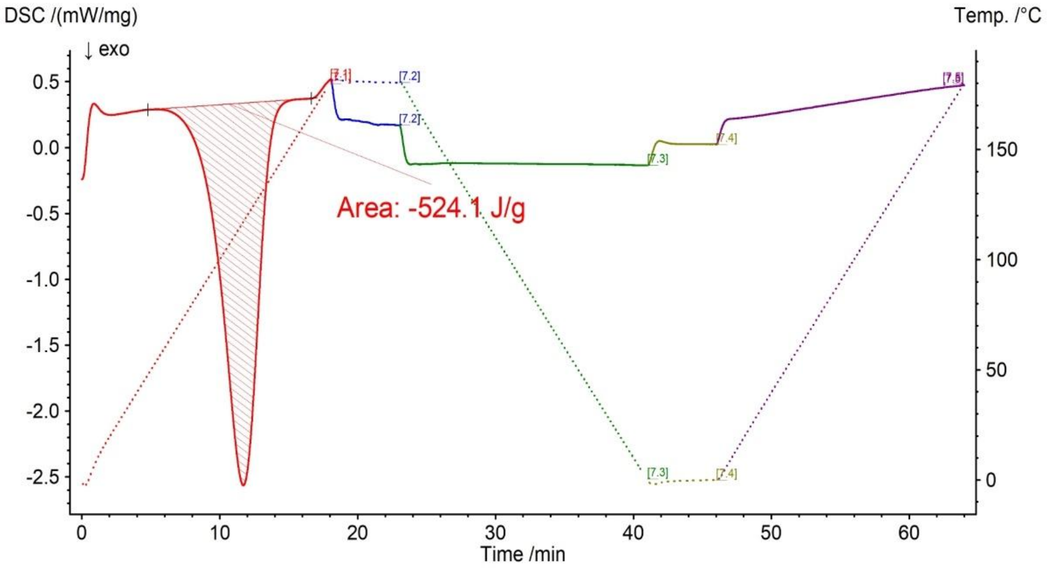

Results

Parameters	Result	Range (min)	Range (max)
**Area (DSC),o**	-524.1 J/g	4.800 min	16.640 min

Figure S45. Thermogram of **4BND-BND** without solvent.

7.13 Thermal analysis of irradiated 4 in solution:

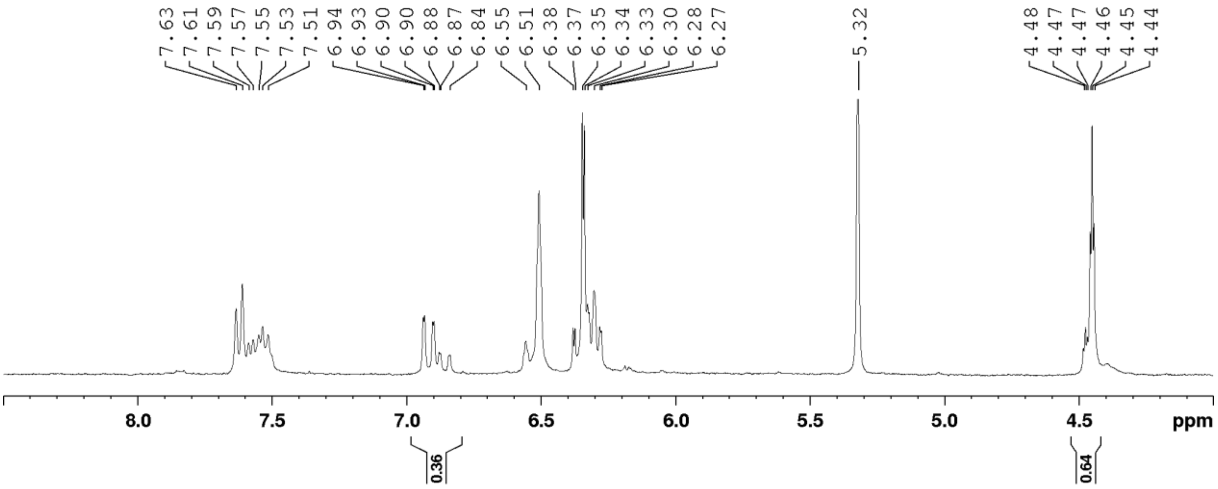

Figure S46. ^{1}H NMR spectra of **4BNB-BNB** and **4BND-BNB** and **4BNB-BNB** in decalin/dichloromethane-d_2 after photoconversion for determination of conversion ratio and before DSC heat release. The conversion ratio of **BNB** into **BND** units is 64% overall.

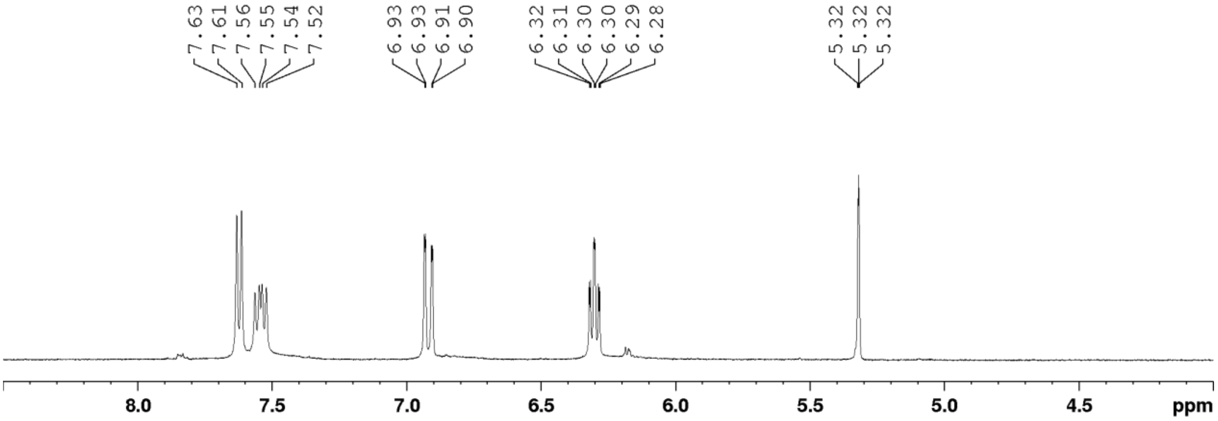

Figure S47. ^{1}H NMR spectra of all the combined DSC samples in decalin/dichloromethane-d_2 after heat release showing only **4**.

Temperature program and thermograms for **4**

A

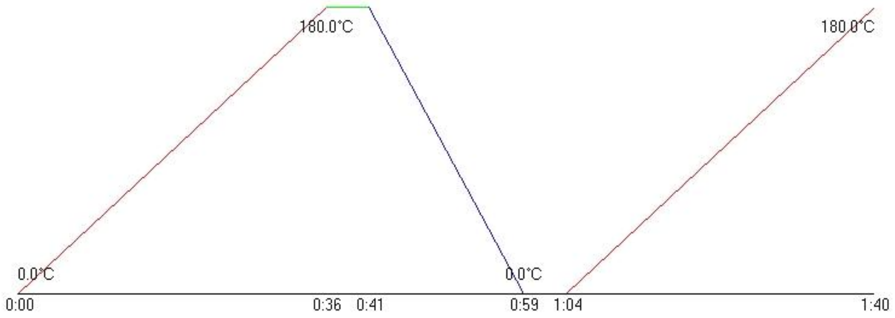

Temperature Steps

Num	Mode	Temp. °C	HR K/min	Acq.Rate pts/min	Duration hh:mm
---	Start	0.0			
**1**	Dynamic	180.0	5.000	150.00	00:36
**2**	Isothermal	180.0		50.00	00:05
**3**	Dynamic	0.0	10.000	300.00	00:18
**4**	Isothermal	0.0		50.00	00:05
**5**	Dynamic	180.0	5.000	150.00	00:36
---	Emergency	190.0			

**Sample1**	
**Sample Mass:**	1.091 mg

B

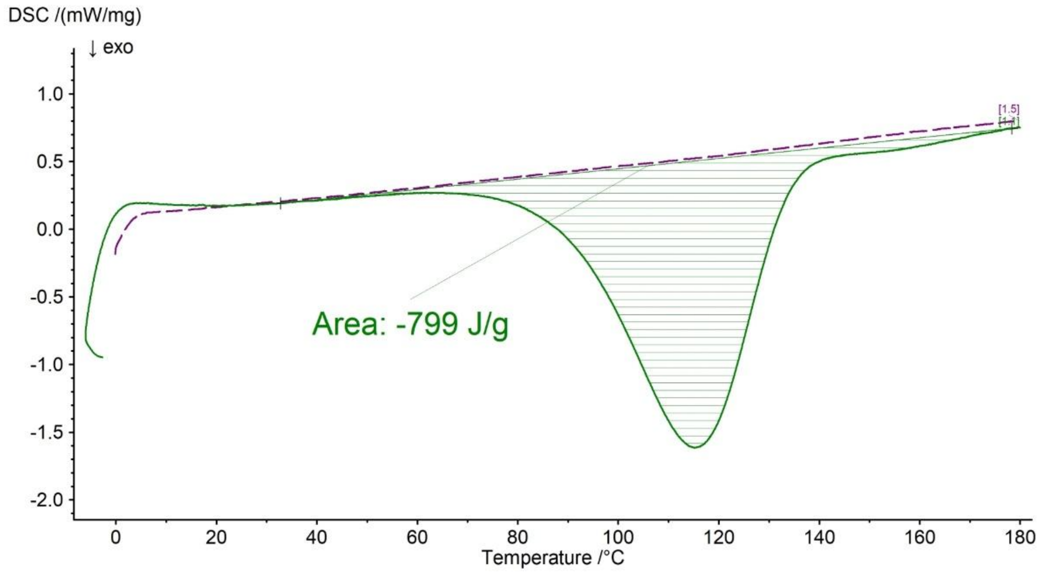

Parameters	Result	Range (min)	Range (max)
**Area (DSC),o**	-799 J/g	32.8 °C	178.4 °C
**Corrected heat release**	-1248 J/g		

Sample2

Sample Mass: 1.118 mg

C

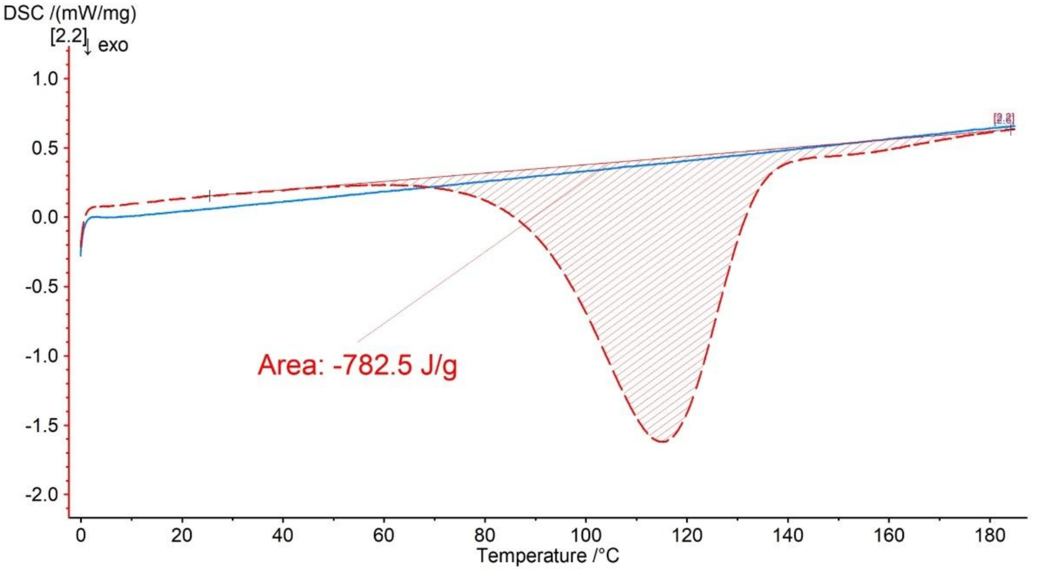

Parameters	Result	Range (min)	Range (max)
**Area (DSC),o**	-782.5 J/g	25.5 °C	184.1 °C
**Corrected heat release**	-1220 J/g		

Sample2

Sample Mass: 1.158 mg

D

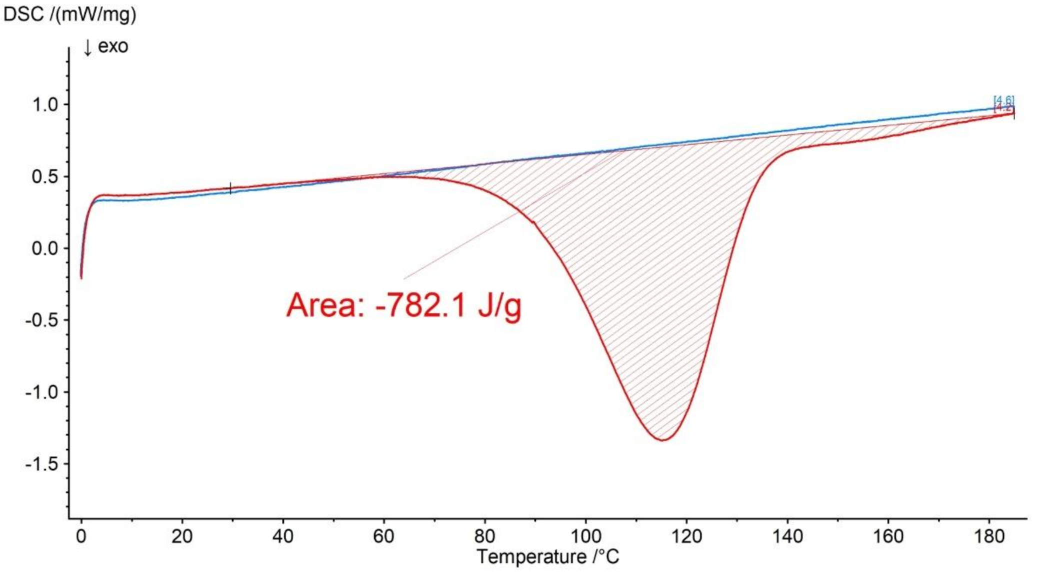

Parameters	Result	Range (min)	Range (max)
**Area (DSC),o**	-782.1 J/g	29.6 °C	185.0 °C
**Corrected heat release**	-1219 J/g		

Figure S48. Temperature program [A] and thermograms [B-D] of **4BND-BND** in decalin.

8. Computational Details

The structures of the reactants, products, intermediates, and transition states were fully optimized without symmetry constraints using the M06-2X[3] functional in conjunction with the 6-311+G**[4] basis set. Harmonic vibrational frequencies were computed analytically to confirm the nature of the stationary points as minimal (no imaginary vibrational frequency) or first order saddle points (one imaginary vibrational frequency). The computation of the intrinsic reaction coordinate (IRC) confirmed the connectivity of the transition states to the corresponding minima. The M06-2X/6-311+G** and TD-DFT computations were performed using the Gaussian 16 program.[6]

The energies were refined by single energy computations at the M06-2X/6-311+G** geometries using the DLPNO-CCSD(T) method[7-9] and the TightPNO truncation criteria in Orca 5.0.1.[10-12] The cc-pVTZ basis set along with the recommended fitting basis set cc-pVTZ/C was employed in these computations.[12, 13-15]

The absolute energies and relative energies computed for the stationary points are given in Tables S3-S6 below.

Table S3. Electronic energies E_{el}, zero-point corrected energies E_0, enthalpies H at 298.15 K and free energies G at 298.15 K computed at the M06-2X/6-311+G** level of theory. The electronic energies E_{el} computed at the DLPNO-CCSD(T)/cc-pVTZ//M06-2X/6-311+G** level of theory are also given. All energies are given in Hartree.

Structures[a]	M062X/6-311+G**				DLPNO-CCSD(T)
**4**	E_{el}	E_0	H	G	E_{el}
R3	-860.68294	-860.26698	-860.24374	-860.31894	-859.23299
TS1_A_3	-860.55395	-860.14335	-860.11974	-860.19530	-859.10460
TS1_A_a	-860.55012	-860.13905	-860.11565	-860.19070	-859.09999
I_A_3	-860.55884	-860.14761	-860.12369	-860.19992	-859.11083
I_A_3_a	-860.55073	-860.13933	-860.11532	-860.19298	-859.10097
TS2_A_3	-860.55012	-860.13905	-860.11565	-860.19070	-859.09999
TS2_A_3_e	-860.55782	-860.14650	-860.12338	-860.19655	-859.10842
TS2_A_f	-860.55719	-860.14610	-860.12274	-860.19797	-859.10840
P_A_3	-860.60103	-860.18755	-860.16413	-860.23984	-859.15097
TS1_B_3	-860.47152	-860.06336	-860.03962	-860.11493	-859.02201
TS1_B_3_a	-860.47085	-860.06292	-860.03895	-860.11651	-859.02229
I_B_3	-860.47704	-860.06818	-860.04414	-860.12001	-859.02875
I_B_3_a	-860.46892	-860.06024	-860.03589	-860.11516	-859.01945
I_B_4_b	-860.47622	-860.06780	-860.04344	-860.12249	-859.02882
TS2_B_3	-860.46705	-860.05885	-860.03492	-860.11334	-859.01759
TS2_B_3_e	-860.47495	-860.06663	-860.04288	-860.11976	-859.02675
P3	-860.51870	-860.10796	-860.08410	-860.16244	-859.06922

[a] The names of the conformers reflect their finding during the search and not numerical order.

Table S4. Relative electronic energies E_{el}, zero-point corrected energies E_0, enthalpies H at 298.15 K and free energies G at 298.15 K computed at the M06-2X/6-311+G** and DLPNO-CCSD(T)/cc-pVTZ//M06-2X/6-311+G** levels of theory. All energies are given in kcal/mol.								
Structure[a]	M062X/6-311+G**				DLPNO-CCSD(T)			
**4**	E_{el}	E_0	H	G	E_{el}	E_0	H	G
R3	0.0	0.0	0.0	0.0	0.0	0.0	0.0	0.0
TS1_A_3	80.9	77.6	77.8	77.6	80.6	77.2	77.4	77.2
TS1_A_3_a	83.3	80.3	80.4	80.5	83.5	80.4	80.5	80.6
I_A_3	77.9	74.9	75.3	74.7	76.7	73.7	74.1	73.5
I_A_3_a	83.0	80.1	80.6	79.0	82.8	80.0	80.5	78.9
TS2_A_3	83.3	80.3	80.4	80.5	83.5	80.4	80.5	80.6
TS2_A_3_e	78.5	75.6	75.5	76.8	78.2	75.3	75.2	76.5
TS2_A_3_f	78.9	75.9	75.9	75.9	78.2	75.1	75.2	75.2
P_A_3	51.4	49.8	50.0	49.6	51.5	49.9	50.0	49.7
TS1_B_3	132.7	127.8	128.1	128.0	132.4	127.5	127.8	127.7
TS1_B_3_a	133.1	128.1	128.5	127.0	132.2	127.2	127.6	126.2
I_B_3	129.2	124.8	125.3	124.8	128.2	123.7	124.2	123.8
I_B_3_a	134.3	129.7	130.4	127.9	134.0	129.4	130.1	127.6
I_B_4_b	129.7	125.0	125.7	123.3	128.1	123.4	124.1	121.7
TS2_B_3	135.5	130.6	131.0	129.0	135.2	130.3	130.7	128.7
TS2_B_3_e	130.5	125.7	126.0	125.0	129.4	124.6	124.9	123.9
P3	103.1	99.8	100.2	98.2	102.8	99.5	99.9	97.9
[a] The names of the conformers reflect their finding during the search and not numerical order.								

Table S5. Electronic energies E_{el}, zero-point corrected energies E_0, enthalpies H at 298.15 K and free energies G at 298.15 K computed at the M06-2X/6-311+G** level of theory. The electronic energies E_{el} computed at the DLPNO-CCSD(T)/cc-pVTZ//M06-2X/6-311+G** level of theory are also given. All energies are given in Hartree.					
Structure[a]	M062X/6-311+G**				DLPNO-CCSD(T)
**3**	E_{el}	E_0	H	G	E_{el}
R4	-1599.38988	-1598.82555	-1598.78995	-1598.89162	-1596.83857
TS2_A_4	-1599.25692	-1598.69773	-1598.66174	-1598.76446	-1596.70673
P_A	-1599.29902	-1598.73663	-1598.70097	-1598.80232	-1596.74912
TS1_B_4	-1599.16445	-1598.60791	-1598.57149	-1598.67675	-1596.61663
TS2_B_4	-1599.16838	-1598.61181	-1598.57533	-1598.68061	-1596.62045
P4	-1599.21114	-1598.65195	-1598.61543	-1598.72147	-1596.66388
[a] The names of the conformers reflect their finding during the search and not numerical order.					

Table S6. Relative electronic energies E_{el}, zero-point corrected energies E_0, enthalpies H at 298.15 K and free energies G at 298.15 K computed at the M06-2X/6-311+G** and DLPNO-CCSD(T)/cc-pVTZ//M06-2X/6-311+G** levels of theory. All energies are given in kcal/mol.								
Structure[a]	M062X/6-311+G**				DLPNO-CCSD(T)			
**3**	E_{el}	E_0	H	G	E_{el}	E_0	H	G
R4	0.0	0.0	0.0	0.0	0.0	0.0	0.0	0.0
TS2_A_4	83.4	80.2	80.5	79.8	82.7	79.5	79.8	79.1
P_A	57.0	55.8	55.8	56.0	56.1	54.9	55.0	55.2
TS1_B_4	141.5	136.6	137.1	134.8	139.3	134.4	134.9	132.6
TS2_B_4	139.0	134.1	134.7	132.4	136.9	132.0	132.6	130.3

P4	112.2	108.9	109.5	106.8	109.6	106.4	107.0	104.2
[a] The names of the conformers reflect their finding during the search and not numerical order.								

9. Computational Results

We identified a reaction pathway for the thermal ring opening of the double Dewar isomer **P** (BND-BND) to the double azaborinine isomer **R** (BNB-BNB) that involves the mono-Dewar intermediate **P_A**. (BND-BNB) For compound **4**, we explored several conformational isomers along the reaction **P → R**. As the energies of these isomers turned out to be very similar, we did not perform a similar analysis for the larger system with TBS substituents.

For the derivative **4**, each individual ring opening is a stepwise process that involves a shallow energy minimum on the reaction coordinate (see Figure S49), similar to the reaction profile computed earlier for the parent azaborinine–Dewar thermal isomerization.[16]

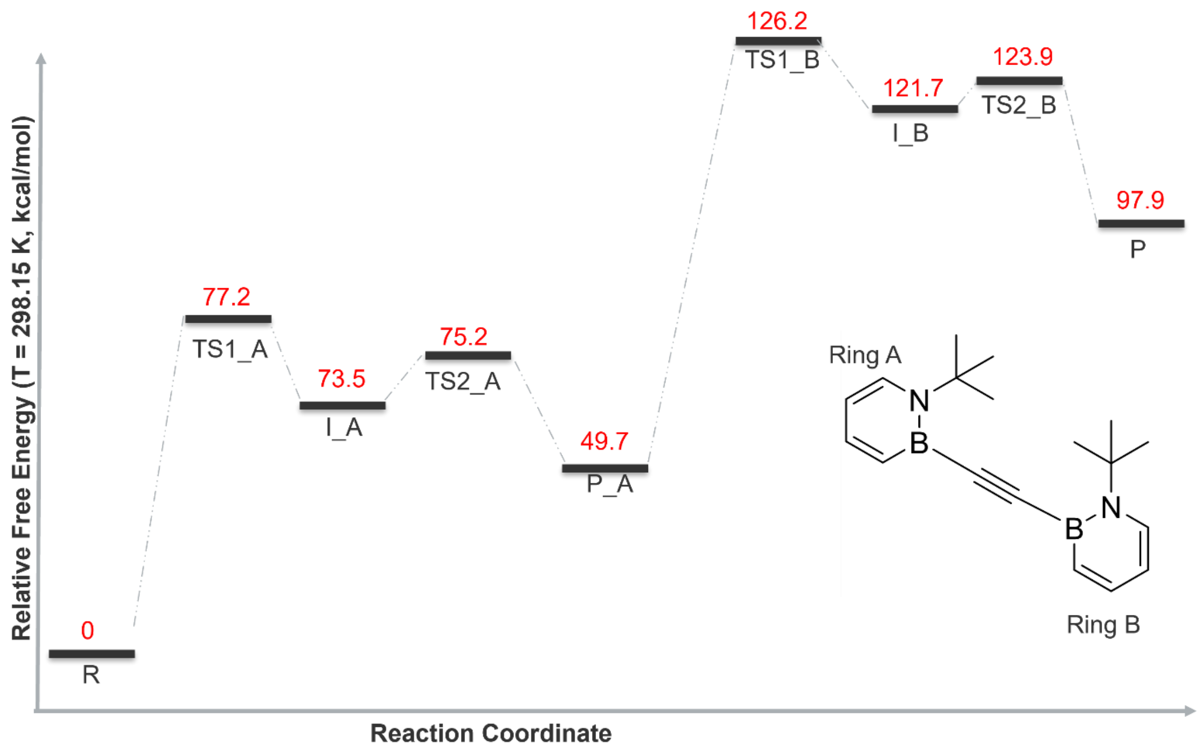

Figure S49. Free energy ΔG°(298.15 K) for the thermal transformation of the double azaborinine R to the double Dewar-isomer P of **4** as computed at the DLPNO-CCSD(T)/cc-pVTZ//M06-2X/6-311+G** level of theory. The correction to G was computed at the M06-2X/6-311+G** level of theory. The diagram is not drawn to scale.

In contrast, the individual ring opening reactions of the TBS-derivative (**3**) proceed in a concerted fashion, similar to the previous finding for the 1-TBS-2-mesityl-1,2-dihydro-1,2-azaborinine (**1**).[17]

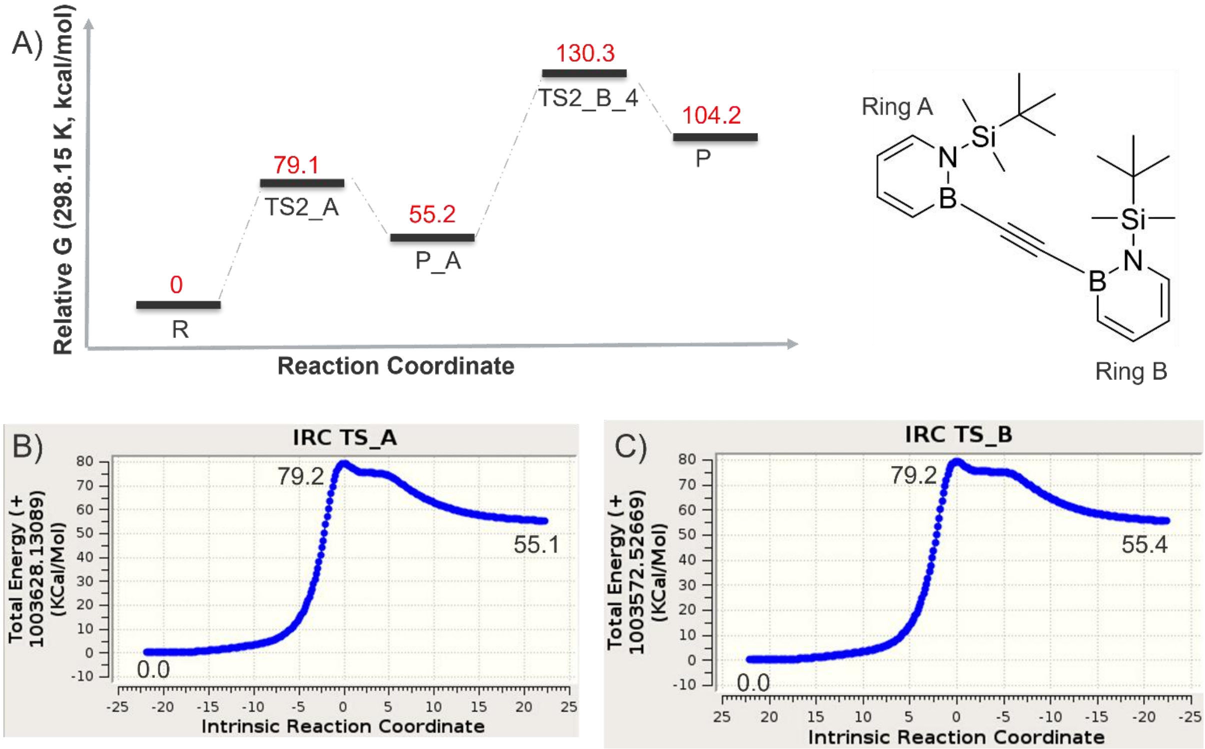

Figure S50. A) Free energy ΔG°(298.15 K) for the thermal transformation of the double azaborinine R to the double Dewar-isomer P of the TBS compound (**3**) as computed at the DLPNO-CCSD(T)/cc-pVTZ//M06-2X/6-311+G** level of theory. The correction to G was computed at the M06-2X/6-311+G** level of theory. The diagram is not drawn to scale. B) Intrinsic reaction coordinate energy profile computed from TS2_A. C) Intrinsic reaction coordinate energy profile computed from TS2_B_4.

We note that the agreement between M06-2X/6-311+G** and DLPNO-CCSD(T)/cc-pVTZ is very good. The mean absolute deviation (MAD) of the relative energy is 0.56 kcal/mol considering all stationary points identified for the system **4**, and 1.68 kcal/mol for the system **3**.

10.Cartesian Coordinates

All structures were optimized at the M06-2X/6-311+G** level of theory and are given in Å.

I. Compound 4, R = tBu

The lowest energy conformers used for the depiction of the potential energy surface (Figure S49) are highlighted on light yellow background.

R3

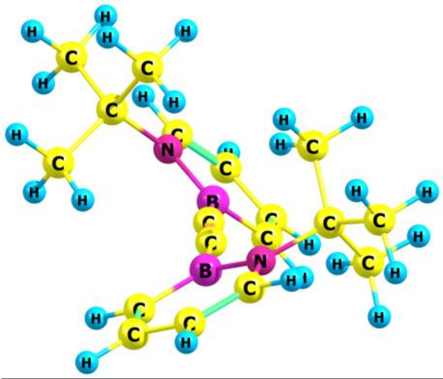

6	-3.635494000	2.370135000	-1.447390000
6	-2.349031000	1.914647000	-1.381315000
6	-4.412480000	0.497848000	-0.131428000
6	-4.677577000	1.650886000	-0.813083000
1	-3.886077000	3.282806000	-1.981275000
1	-1.564211000	2.477882000	-1.875561000
1	-5.230412000	-0.023553000	0.339189000
1	-5.701526000	1.997280000	-0.855004000
7	-3.165416000	-0.045862000	-0.004570000
5	-2.046387000	0.637655000	-0.628108000
6	-0.591358000	0.141734000	-0.562146000
6	0.591441000	-0.141666000	-0.562081000
6	2.349242000	-1.914455000	-1.381246000
6	3.635717000	-2.369928000	-1.447197000
1	1.564497000	-2.477623000	-1.875689000
6	4.412501000	-0.497826000	-0.130854000
6	4.677704000	-1.650766000	-0.812633000
1	3.886383000	-3.282521000	-1.981177000
1	5.230361000	0.023511000	0.339960000
1	5.701659000	-1.997150000	-0.854452000
5	2.046482000	-0.637572000	-0.627901000
7	3.165416000	0.045862000	-0.004102000
6	-2.998859000	-1.328626000	0.773768000
6	2.998740000	1.328517000	0.774388000
6	-4.324890000	-1.856031000	1.331814000
1	-4.785113000	-1.162070000	2.038604000
1	-5.040921000	-2.098224000	0.543524000
1	-4.108730000	-2.778624000	1.872323000
6	-2.067494000	-1.065928000	1.963261000
1	-1.074868000	-0.760136000	1.638482000
1	-2.487428000	-0.284678000	2.601687000
1	-1.966920000	-1.979063000	2.554763000
6	-2.428785000	-2.402939000	-0.160293000
1	-1.450130000	-2.126063000	-0.547559000
1	-2.327382000	-3.342941000	0.387433000
1	-3.107841000	-2.564631000	-1.001067000
6	4.324687000	1.855851000	1.332700000
1	4.784810000	1.161795000	2.039462000
1	5.040832000	2.098155000	0.544548000
1	4.108444000	2.778369000	1.873305000
6	2.428799000	2.402957000	-0.159608000
1	1.450202000	2.126130000	-0.547058000
1	2.327310000	3.342883000	0.388234000
1	3.107979000	2.564769000	-1.000259000
6	2.067202000	1.065651000	1.963708000
1	1.966536000	1.978704000	2.555320000
1	1.074626000	0.759896000	1.638742000
1	2.487048000	0.284317000	2.602090000

TS1_A_3

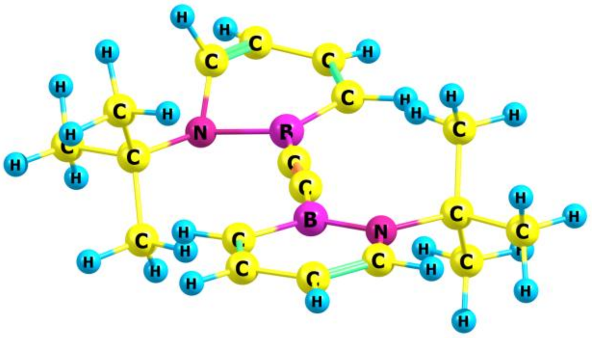

6	-4.452973000	-1.957297000	-0.418410000
6	-3.775193000	-0.663293000	-0.733813000
6	-2.230636000	-2.481725000	0.064399000
6	-3.600993000	-2.924518000	-0.048386000
1	-5.532226000	-1.952053000	-0.314452000
1	-3.930063000	-3.918732000	0.236239000
1	-3.843432000	-0.220527000	-1.734088000
1	-1.413467000	-3.194402000	0.064947000
5	-2.000618000	-1.042921000	0.175036000
7	-3.264680000	0.004744000	0.272533000
6	-0.627068000	-0.381462000	0.087697000
6	0.504791000	0.060098000	0.027525000
6	1.901748000	2.274247000	-0.182230000
6	4.286261000	0.813212000	-0.109902000
6	3.105344000	2.917110000	-0.259744000
1	0.988818000	2.861358000	-0.210985000
6	4.310314000	2.174582000	-0.222574000
1	5.223683000	0.281070000	-0.084001000
1	3.165567000	3.998422000	-0.349384000
1	5.271817000	2.666710000	-0.281754000
5	1.865931000	0.766354000	-0.057319000
7	3.142141000	0.073179000	-0.026816000
6	-3.117903000	1.490986000	0.198661000
6	3.242608000	-1.428716000	0.093490000
6	-2.328759000	1.952616000	1.421923000
1	-2.760983000	1.520025000	2.326711000
1	-1.281234000	1.663130000	1.359738000
1	-2.385561000	3.041109000	1.496546000
6	-2.478155000	1.993771000	-1.100581000
1	-3.093820000	1.773501000	-1.976056000
1	-2.386395000	3.080776000	-1.040721000
1	-1.484257000	1.571288000	-1.245093000
6	-4.554441000	2.022815000	0.300924000
1	-5.028313000	1.675015000	1.220865000
1	-4.542685000	3.114529000	0.301926000
1	-5.156631000	1.688755000	-0.549118000
6	2.595200000	-1.862524000	1.414147000
1	1.538047000	-1.606867000	1.451652000
1	2.689627000	-2.945462000	1.524989000
1	3.104095000	-1.384243000	2.254752000
6	2.543340000	-2.068669000	-1.112184000
1	2.637879000	-3.155498000	-1.049978000
1	1.484534000	-1.819242000	-1.147428000
1	3.015943000	-1.734064000	-2.039039000
6	4.693364000	-1.921660000	0.103792000
1	5.221778000	-1.680009000	-0.821078000
1	5.258278000	-1.536004000	0.955331000
1	4.669109000	-3.008636000	0.192474000

TS1_A_3_a

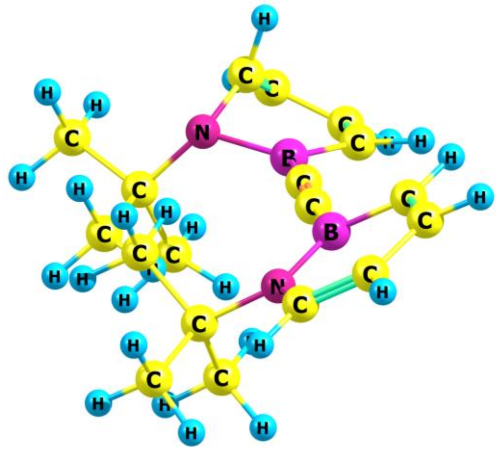

6	-4.730716000	1.419339000	0.087464000
6	-3.561325000	1.109013000	0.932467000
6	-2.717146000	2.231184000	-0.664859000
6	-4.171461000	2.215687000	-0.838843000
1	-5.713066000	0.970760000	0.135045000
1	-4.681597000	2.735424000	-1.644335000
1	-3.307828000	1.778099000	1.751141000
1	-2.185445000	3.145620000	-0.931240000
5	-1.914878000	1.103023000	-0.062675000
7	-2.719062000	0.055948000	0.702959000
6	-0.383261000	1.096697000	0.012209000
6	0.831354000	1.037900000	0.037353000
6	3.062047000	2.417754000	0.267108000
6	4.570250000	0.072051000	0.044269000
6	4.426339000	2.468619000	0.320356000
1	2.495647000	3.338808000	0.354631000
6	5.186929000	1.279390000	0.206714000
1	5.180807000	-0.812582000	-0.039827000
1	4.953147000	3.410486000	0.447655000
1	6.267838000	1.301275000	0.244939000
5	2.369082000	1.084716000	0.088855000
7	3.215386000	-0.091819000	-0.019536000
6	-3.111323000	-1.266617000	0.155760000
6	2.652160000	-1.480952000	-0.195554000
6	-3.610809000	-1.232157000	-1.295842000
1	-4.573926000	-0.731544000	-1.389119000
1	-2.890658000	-0.717593000	-1.936380000
1	-3.723401000	-2.256154000	-1.661678000
6	-1.841980000	-2.118847000	0.225338000
1	-1.472548000	-2.162225000	1.251610000
1	-2.047215000	-3.133348000	-0.125449000
1	-1.055920000	-1.686095000	-0.399487000
6	-4.184562000	-1.848949000	1.075270000
1	-5.092774000	-1.240193000	1.051200000
1	-4.447944000	-2.859633000	0.755282000
1	-3.821382000	-1.890588000	2.104275000
6	1.778536000	-1.815042000	1.019774000
1	0.954294000	-1.113499000	1.132559000
1	1.362061000	-2.819096000	0.903246000
1	2.384338000	-1.795127000	1.929235000
6	1.849304000	-1.525770000	-1.501346000
1	1.436432000	-2.528243000	-1.640238000
1	1.027575000	-0.812306000	-1.497516000
1	2.502396000	-1.300979000	-2.348323000
6	3.741970000	-2.554155000	-0.289717000
1	4.397144000	-2.408130000	-1.151288000
1	4.345719000	-2.615417000	0.618276000
1	3.243167000	-3.516187000	-0.416604000

I_A_3

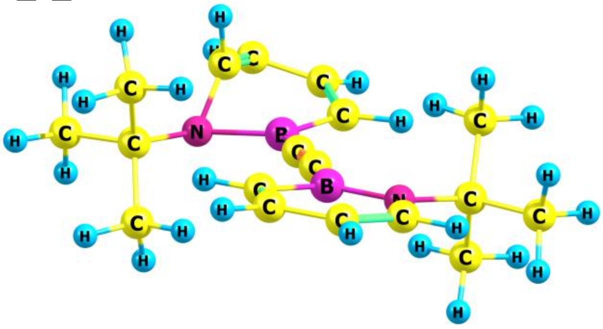

6	-4.339797000	-1.951609000	-0.545791000
6	-3.520820000	-0.748772000	-0.820273000
6	-2.210181000	-2.472521000	0.090002000
6	-3.540402000	-2.961424000	-0.138314000
1	-5.414802000	-1.874850000	-0.428394000
1	-3.871887000	-3.961993000	0.117686000
1	-3.234704000	-0.434761000	-1.826059000
1	-1.359369000	-3.150955000	0.102549000
5	-2.035312000	-0.991008000	0.183160000
7	-3.304291000	-0.014268000	0.292640000
6	-0.641053000	-0.360309000	0.065474000
6	0.494299000	0.069669000	0.003475000
6	1.888488000	2.279652000	-0.259489000
6	4.277311000	0.830595000	-0.122317000
6	3.090402000	2.924522000	-0.345642000
1	0.973335000	2.861282000	-0.312938000
6	4.297514000	2.187846000	-0.275846000
1	5.216207000	0.302922000	-0.071833000
1	3.147564000	4.002909000	-0.466688000
1	5.257835000	2.681526000	-0.341279000
5	1.856598000	0.776460000	-0.090076000
7	3.134563000	0.089153000	-0.027148000
6	-3.180931000	1.468406000	0.248617000
6	3.239976000	-1.407640000	0.139614000
6	-2.412631000	1.921473000	1.487992000
1	-2.841422000	1.456153000	2.378221000
1	-1.358105000	1.654500000	1.425378000
1	-2.491275000	3.006371000	1.590517000
6	-2.544521000	2.015397000	-1.033909000
1	-3.159672000	1.800439000	-1.911985000
1	-2.471982000	3.102426000	-0.951275000
1	-1.541952000	1.617321000	-1.192182000
6	-4.629622000	1.967345000	0.340559000
1	-5.095608000	1.616067000	1.262712000
1	-4.649288000	3.059641000	0.326320000
1	-5.216050000	1.601355000	-0.507084000
6	2.581278000	-1.803667000	1.466520000
1	1.523930000	-1.546810000	1.487009000
1	2.675797000	-2.882783000	1.610273000
1	3.082082000	-1.300217000	2.297164000
6	2.555147000	-2.086286000	-1.053101000
1	2.648845000	-3.170645000	-0.955442000
1	1.497248000	-1.836600000	-1.108755000
1	3.038528000	-1.781165000	-1.984523000
6	4.692137000	-1.894962000	0.179061000
1	5.228871000	-1.679548000	-0.747448000
1	5.247236000	-1.481453000	1.023902000
1	4.670742000	-2.978870000	0.300514000

I_A_3_a

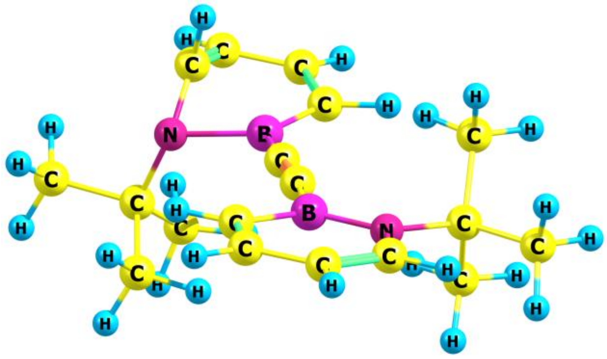

6	-4.415150000	-1.410282000	-1.087027000
6	-3.461861000	-0.344973000	-1.403789000
6	-2.282447000	-2.038880000	-0.334666000
6	-3.655562000	-2.418007000	-0.586295000
1	-5.492480000	-1.328497000	-1.102261000
1	-4.055526000	-3.390600000	-0.314184000
1	-2.902059000	-0.451823000	-2.336454000
1	-1.550214000	-2.829874000	-0.184270000
5	-1.842624000	-0.617475000	-0.400728000
7	-2.968847000	0.557296000	-0.559485000
6	-0.397673000	-0.125341000	-0.391147000
6	0.760743000	0.242711000	-0.350652000
6	2.375278000	2.287199000	-0.712077000
6	4.580146000	0.710415000	-0.023180000
6	3.631375000	2.824725000	-0.709571000
1	1.532292000	2.914537000	-0.982018000
6	4.745526000	2.023479000	-0.360218000
1	5.451281000	0.131290000	0.237728000
1	3.803001000	3.865357000	-0.971351000
1	5.747307000	2.431792000	-0.352279000
5	2.181284000	0.833196000	-0.340761000
7	3.371481000	0.074512000	0.005939000
6	-3.668160000	1.099138000	0.634398000
6	3.319276000	-1.383441000	0.393497000
6	-4.318172000	0.078459000	1.579278000
1	-5.219409000	-0.367786000	1.160598000
1	-3.617387000	-0.718016000	1.835447000
1	-4.599421000	0.597515000	2.499117000
6	-2.601024000	1.882533000	1.401478000
1	-2.092130000	2.580746000	0.735012000
1	-3.061426000	2.437606000	2.221680000
1	-1.850797000	1.205792000	1.817958000
6	-4.733558000	2.054541000	0.089021000
1	-5.475535000	1.506463000	-0.499351000
1	-5.249897000	2.548918000	0.914916000
1	-4.276173000	2.814170000	-0.547545000
6	2.453073000	-1.529859000	1.650477000
1	1.429985000	-1.202045000	1.477774000
1	2.431300000	-2.578983000	1.955536000
1	2.878774000	-0.941879000	2.467422000
6	2.747899000	-2.186302000	-0.781578000
1	2.728879000	-3.247329000	-0.520548000
1	1.735104000	-1.875279000	-1.029748000
1	3.381508000	-2.059346000	-1.662895000
6	4.702643000	-1.958186000	0.716865000
1	5.378836000	-1.923980000	-0.140131000
1	5.171699000	-1.460621000	1.568538000
1	4.566646000	-3.006959000	0.984688000

TS2_A_3

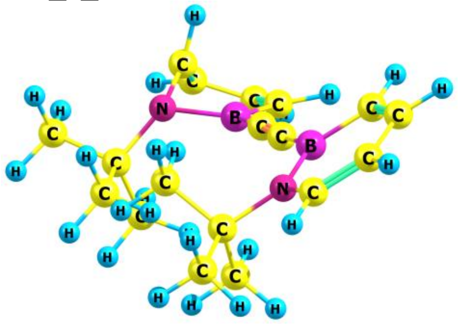

6	4.730715000	1.419339000	-0.087467000
6	3.561323000	1.109014000	-0.932470000
6	2.717146000	2.231183000	0.664858000
6	4.171462000	2.215687000	0.838841000
1	5.713066000	0.970760000	-0.135050000
1	4.681598000	2.735424000	1.644332000
1	3.307826000	1.778099000	-1.751143000
1	2.185446000	3.145620000	0.931239000
5	1.914878000	1.103023000	0.062675000
7	2.719061000	0.055949000	-0.702961000
6	0.383261000	1.096696000	-0.012208000
6	-0.831354000	1.037900000	-0.037352000
6	-3.062048000	2.417755000	-0.267104000
6	-4.570251000	0.072051000	-0.044264000
6	-4.426339000	2.468619000	-0.320351000
1	-2.495647000	3.338808000	-0.354627000
6	-5.186930000	1.279390000	-0.206707000
1	-5.180807000	-0.812581000	0.039832000
1	-4.953148000	3.410487000	-0.447648000
1	-6.267839000	1.301275000	-0.244931000
5	-2.369082000	1.084716000	-0.088852000
7	-3.215386000	-0.091819000	0.019538000
6	-2.652160000	-1.480952000	0.195555000
6	3.111324000	-1.266617000	-0.155763000
6	-3.741970000	-2.554155000	0.289717000
1	-4.397143000	-2.408131000	1.151289000
1	-4.345720000	-2.615415000	-0.618275000
1	-3.243167000	-3.516188000	0.416603000
6	-1.849303000	-1.525771000	1.501346000
1	-1.027575000	-0.812307000	1.497517000
1	-2.502394000	-1.300982000	2.348324000
1	-1.436431000	-2.528244000	1.640238000
6	-1.778537000	-1.815041000	-1.019774000
1	-0.954294000	-1.113498000	-1.132558000
1	-1.362062000	-2.819095000	-0.903247000
1	-2.384339000	-1.795125000	-1.929234000
6	1.841980000	-2.118847000	-0.225341000
1	2.047216000	-3.133349000	0.125445000
1	1.055921000	-1.686097000	0.399486000
1	1.472547000	-2.162224000	-1.251613000
6	3.610811000	-1.232159000	1.295838000
1	3.723404000	-2.256155000	1.661674000
1	4.573928000	-0.731544000	1.389114000
1	2.890661000	-0.717595000	1.936377000
6	4.184561000	-1.848948000	-1.075275000
1	3.821379000	-1.890585000	-2.104280000
1	5.092773000	-1.240192000	-1.051206000
1	4.447944000	-2.859633000	-0.755289000

TS2_A_3_e

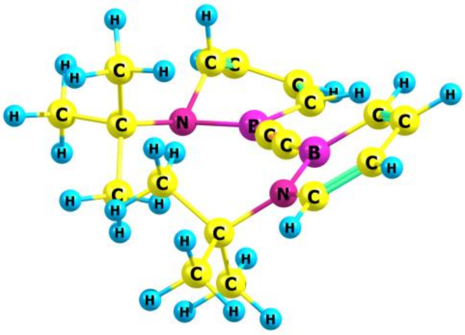

6	4.794321000	1.503983000	0.170273000
6	3.652378000	0.839433000	-0.508036000
6	2.788963000	2.314457000	0.768996000
6	4.228753000	2.482506000	0.888793000
1	5.790933000	1.081478000	0.210724000
1	4.713467000	3.213733000	1.525553000
1	3.405838000	1.012950000	-1.556540000
1	2.131679000	3.182828000	0.734344000
5	2.168696000	0.959541000	0.523842000
7	3.108603000	-0.199266000	0.226200000
6	0.655802000	0.897789000	0.243333000
6	-0.550927000	0.908902000	0.094600000
6	-2.581663000	2.455550000	-0.530530000
6	-4.366830000	0.333088000	-0.168690000
6	-3.921706000	2.642693000	-0.722324000
1	-1.905956000	3.291821000	-0.675217000
6	-4.822762000	1.566508000	-0.536693000
1	-5.080999000	-0.463675000	-0.036094000
1	-4.321918000	3.609268000	-1.016040000
1	-5.886888000	1.697009000	-0.681185000
5	-2.066619000	1.093920000	-0.120485000
7	-3.050329000	0.037833000	0.045962000
6	-2.669159000	-1.369581000	0.433554000
6	2.677101000	-1.470903000	-0.404997000
6	-3.880034000	-2.299932000	0.559776000
1	-4.575350000	-1.973935000	1.336303000
1	-4.418261000	-2.414962000	-0.383467000
1	-3.507196000	-3.284587000	0.846004000
6	-1.972424000	-1.335591000	1.799161000
1	-1.081887000	-0.710527000	1.789393000
1	-2.658891000	-0.949087000	2.556566000
1	-1.679260000	-2.349129000	2.084121000
6	-1.758481000	-1.942772000	-0.658990000
1	-0.862166000	-1.340968000	-0.793321000
1	-1.453860000	-2.957193000	-0.389036000
1	-2.297348000	-1.983076000	-1.608988000
6	1.986532000	-1.289943000	-1.763104000
1	1.632997000	-2.261134000	-2.118025000
1	1.131807000	-0.615721000	-1.698559000
1	2.680797000	-0.905005000	-2.515045000
6	1.763194000	-2.184566000	0.588629000
1	1.509302000	-3.179397000	0.214050000
1	2.273371000	-2.290935000	1.548496000
1	0.839496000	-1.627428000	0.746216000
6	3.956463000	-2.291516000	-0.601175000
1	4.649454000	-1.765987000	-1.265041000
1	4.452663000	-2.454000000	0.357234000
1	3.722745000	-3.260245000	-1.049824000

TS2_A_3_f

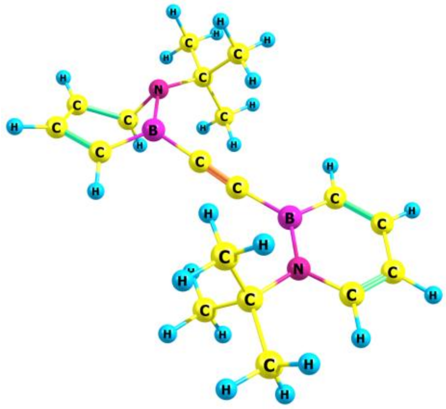

6	4.406296000	2.001715000	-0.385021000
6	3.518922000	0.863441000	-0.737724000
6	2.231571000	2.357849000	0.018222000
6	3.556742000	2.961802000	-0.000346000
1	5.479282000	1.911681000	-0.267738000
1	3.782354000	3.968501000	0.332449000
1	3.275024000	0.605619000	-1.769264000
1	1.357463000	2.935508000	-0.283831000
5	2.027140000	0.885708000	0.294142000
7	3.262423000	0.011317000	0.324253000
6	0.612583000	0.301033000	0.148108000
6	-0.529146000	-0.108611000	0.077575000
6	-1.959291000	-2.299842000	-0.144297000
6	-4.322868000	-0.804212000	-0.119534000
6	-3.170839000	-2.925886000	-0.239774000
1	-1.053855000	-2.898594000	-0.154435000
6	-4.364559000	-2.165612000	-0.226934000
1	-5.252653000	-0.258460000	-0.112145000
1	-3.245234000	-4.006533000	-0.325638000
1	-5.332128000	-2.643742000	-0.300725000
5	-1.904437000	-0.793516000	-0.025676000
7	-3.169185000	-0.080786000	-0.019423000
6	-3.251036000	1.422819000	0.093598000
6	3.242577000	-1.466124000	0.167730000
6	-4.694643000	1.936326000	0.082656000
1	-5.213995000	1.699670000	-0.848541000
1	-5.276290000	1.561089000	0.927491000
1	-4.656236000	3.023120000	0.168770000
6	-2.527530000	2.047077000	-1.105784000
1	-1.473717000	1.776232000	-1.128309000
1	-2.995611000	1.718697000	-2.037075000
1	-2.602346000	3.135692000	-1.047459000
6	-2.615500000	1.853372000	1.420986000
1	-1.562596000	1.583539000	1.473582000
1	-2.697472000	2.937813000	1.526933000
1	-3.141500000	1.384854000	2.256442000
6	2.584762000	-1.948904000	-1.131106000
1	2.594077000	-3.041421000	-1.147597000
1	1.549669000	-1.614620000	-1.208537000
1	3.136756000	-1.610152000	-2.012143000
6	2.528497000	-2.049511000	1.384895000
1	2.628607000	-3.137502000	1.385241000
1	2.976782000	-1.658207000	2.300749000
1	1.466951000	-1.799110000	1.378870000
6	4.712705000	-1.898887000	0.177630000
1	5.251272000	-1.443708000	-0.659220000
1	5.192390000	-1.587031000	1.107125000
1	4.791360000	-2.984687000	0.083592000

P_A_3

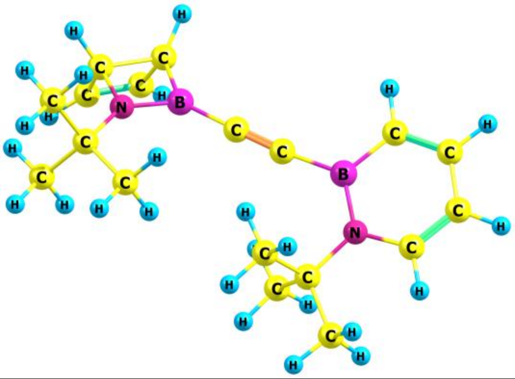

6	4.398318000	1.307848000	1.387992000
6	4.020742000	1.162103000	-0.079946000
6	2.958009000	2.281646000	0.179530000
6	3.505336000	2.281062000	1.594166000
1	5.081088000	0.764211000	2.029044000
1	3.214852000	2.847849000	2.471042000
1	4.789876000	1.284284000	-0.843827000
1	2.979181000	3.206244000	-0.394131000
5	1.924221000	1.100741000	-0.176876000
7	2.961799000	0.184435000	-0.393129000
6	0.406725000	0.974172000	-0.243859000
6	-0.807766000	0.919189000	-0.260769000
6	-2.997098000	2.273882000	-0.790525000
6	-4.360395000	2.363296000	-0.831540000
1	-2.402138000	3.125060000	-1.103804000
6	-4.576571000	0.108440000	0.010608000
6	-5.156439000	1.266563000	-0.422791000
1	-4.858318000	3.267349000	-1.171245000
1	-5.213969000	-0.706165000	0.314645000
1	-6.236637000	1.321241000	-0.444198000
5	-2.347198000	0.997116000	-0.308646000
7	-3.226932000	-0.088384000	0.086281000
6	3.159859000	-1.243392000	-0.620573000
6	-2.706087000	-1.424230000	0.558676000
6	3.944525000	-1.441242000	-1.922236000
1	4.919296000	-0.950099000	-1.867742000
1	4.112696000	-2.504982000	-2.107597000
1	3.391611000	-1.021275000	-2.765113000
6	3.945632000	-1.833172000	0.555437000
1	3.417122000	-1.655464000	1.495002000
1	4.075260000	-2.909633000	0.420494000
1	4.938018000	-1.379929000	0.626745000
6	1.789802000	-1.906857000	-0.729008000
1	1.217078000	-1.768334000	0.191691000
1	1.217942000	-1.471698000	-1.552022000
1	1.907292000	-2.977507000	-0.912667000
6	-1.850823000	-1.210382000	1.813215000
1	-2.450188000	-0.744739000	2.599419000
1	-1.495634000	-2.177307000	2.178069000
1	-0.985958000	-0.580739000	1.614672000
6	-1.893520000	-2.055657000	-0.577524000
1	-1.493168000	-3.018913000	-0.250950000
1	-2.534523000	-2.223315000	-1.446604000
1	-1.060582000	-1.421348000	-0.873534000
6	-3.828230000	-2.400347000	0.926904000
1	-4.442677000	-2.035322000	1.752864000
1	-4.469709000	-2.638803000	0.075918000
1	-3.359116000	-3.329845000	1.252844000

TS1_B_3

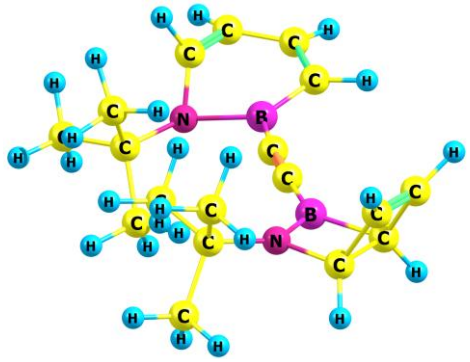

6	4.525708000	1.752208000	0.554140000
6	3.961128000	0.935490000	-0.600140000
6	2.946983000	2.108624000	-0.810642000
6	3.671097000	2.760201000	0.351816000
1	5.282059000	1.544060000	1.300764000
1	3.497409000	3.699830000	0.863116000
1	4.626305000	0.615681000	-1.403676000
1	2.898244000	2.632736000	-1.763142000
5	1.867673000	0.978059000	-0.424824000
7	2.865824000	0.004100000	-0.272523000
6	0.352162000	0.947947000	-0.290545000
6	-0.862821000	1.002428000	-0.242358000
5	-2.379113000	1.208439000	-0.226083000
6	-3.016933000	2.513650000	-0.081965000
7	-3.283394000	-0.166400000	-0.260148000
6	-4.447858000	2.534122000	0.118753000
1	-2.447337000	3.434601000	-0.123502000
6	-3.910593000	0.324430000	0.781441000
6	-4.954813000	1.361946000	0.525941000
1	-5.070131000	3.387393000	-0.130781000
1	-3.790104000	-0.118339000	1.776800000
1	-5.990235000	1.041925000	0.494708000
6	3.029969000	-1.366805000	0.198081000
6	-2.697497000	-1.539781000	-0.204304000
6	3.565436000	-2.232104000	-0.948522000
1	4.526758000	-1.853709000	-1.304879000
1	3.710976000	-3.262365000	-0.614048000
1	2.862048000	-2.229678000	-1.783989000
6	4.020664000	-1.379973000	1.366912000
1	3.668467000	-0.732307000	2.173234000
1	4.134952000	-2.395404000	1.753641000
1	5.004886000	-1.027658000	1.046330000
6	1.668720000	-1.883430000	0.653813000
1	1.282413000	-1.272329000	1.473123000
1	0.947459000	-1.846809000	-0.166385000
1	1.755978000	-2.917293000	0.997082000
6	-1.874658000	-1.754270000	-1.472628000
1	-2.477998000	-1.512960000	-2.350314000
1	-0.980859000	-1.132971000	-1.483614000
1	-1.573970000	-2.802873000	-1.535076000
6	-3.908040000	-2.482721000	-0.230424000
1	-4.537677000	-2.335194000	0.651844000
1	-4.512623000	-2.308381000	-1.122485000
1	-3.565239000	-3.519425000	-0.234360000
6	-1.868853000	-1.804411000	1.057890000
1	-2.486097000	-1.817526000	1.959399000
1	-1.405047000	-2.789980000	0.972024000
1	-1.079263000	-1.062729000	1.173855000

TS1_B_3_a

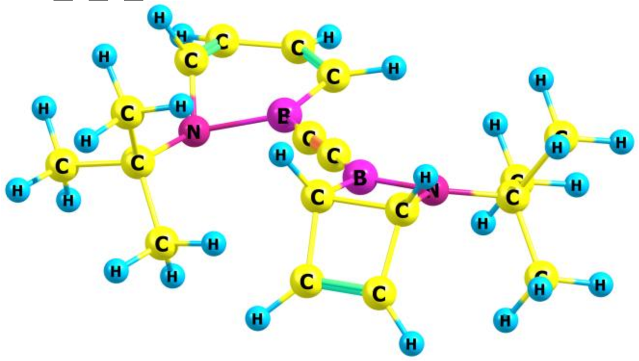

6	-3.972606000	-2.147889000	0.426649000
6	-3.707670000	-1.160850000	-0.702490000
6	-2.336956000	-1.893142000	-0.893833000
6	-2.811550000	-2.783250000	0.239509000
1	-4.771025000	-2.236508000	1.152814000
1	-2.329878000	-3.611007000	0.747248000
1	-4.423501000	-1.085998000	-1.522407000
1	-2.085695000	-2.346871000	-1.850884000
5	-1.744529000	-0.457904000	-0.461556000
7	-3.025953000	0.093078000	-0.335159000
6	-0.351463000	0.111652000	-0.245145000
6	0.788000000	0.506643000	-0.084823000
5	2.177948000	1.105061000	0.132159000
6	2.470949000	2.534046000	0.068294000
7	3.388038000	0.007107000	0.308834000
6	3.862796000	2.922816000	0.067283000
1	1.684657000	3.278432000	0.018672000
6	4.002671000	0.665584000	-0.643840000
6	4.701965000	1.928717000	-0.255967000
1	4.207331000	3.898502000	0.394279000
1	4.137376000	0.230306000	-1.640637000
1	5.770119000	1.880710000	-0.075912000
6	-3.673624000	1.307021000	0.152994000
6	3.193455000	-1.471465000	0.206207000
6	-4.506795000	1.918985000	-0.978738000
1	-5.270183000	1.217340000	-1.324637000
1	-5.012279000	2.823831000	-0.632488000
1	-3.864806000	2.177608000	-1.823402000
6	-4.583464000	0.947284000	1.332401000
1	-4.006784000	0.465611000	2.125536000
1	-5.052752000	1.848071000	1.734879000
1	-5.377616000	0.264262000	1.018293000
6	-2.591028000	2.287030000	0.597396000
1	-1.991561000	1.863864000	1.406289000
1	-1.918632000	2.523035000	-0.230195000
1	-3.052591000	3.212677000	0.948961000
6	2.296093000	-1.910725000	1.360865000
1	2.680108000	-1.511028000	2.301758000
1	1.271112000	-1.569948000	1.226057000
1	2.295008000	-3.001496000	1.421417000
6	4.597091000	-2.058367000	0.410735000
1	5.272786000	-1.743491000	-0.389933000
1	5.014087000	-1.733253000	1.365815000
1	4.544203000	-3.148802000	0.402538000
6	2.635872000	-1.937862000	-1.143353000
1	3.319177000	-1.722676000	-1.968421000
1	2.510150000	-3.022415000	-1.107593000
1	1.667069000	-1.485199000	-1.351646000

I_B_3

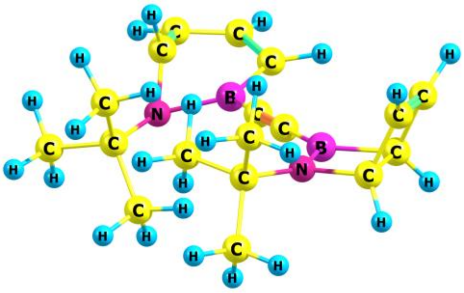

6	4.514761000	1.743487000	0.560360000
6	3.955847000	0.926103000	-0.596171000
6	2.944113000	2.099946000	-0.814031000
6	3.662719000	2.752300000	0.351812000
1	5.266738000	1.535215000	1.311323000
1	3.488029000	3.693017000	0.860843000
1	4.625269000	0.604818000	-1.395506000
1	2.902709000	2.622711000	-1.767684000
5	1.862211000	0.970018000	-0.433442000
7	2.857857000	-0.003857000	-0.273840000
6	0.344628000	0.935446000	-0.305691000
6	-0.869853000	0.972627000	-0.257130000
5	-2.399490000	1.134603000	-0.272049000
6	-3.004869000	2.499483000	-0.200502000
7	-3.321739000	-0.178438000	-0.267668000
6	-4.401428000	2.579151000	0.122844000
1	-2.399582000	3.398250000	-0.294607000
6	-3.666824000	0.506389000	0.846301000
6	-4.828096000	1.396040000	0.614654000
1	-5.034985000	3.423417000	-0.126350000
1	-3.231438000	0.332570000	1.832087000
1	-5.836140000	0.997789000	0.591725000
6	3.017413000	-1.376459000	0.194585000
6	-2.754898000	-1.551674000	-0.199842000
6	3.557758000	-2.239061000	-0.951708000
1	4.521512000	-1.861281000	-1.302192000
1	3.699967000	-3.270300000	-0.618991000
1	2.858413000	-2.233849000	-1.790493000
6	4.002683000	-1.392680000	1.367878000
1	3.647039000	-0.746824000	2.174161000
1	4.114242000	-2.409052000	1.752813000
1	4.988775000	-1.040434000	1.052852000
6	1.653421000	-1.892879000	0.642327000
1	1.263642000	-1.284002000	1.461632000
1	0.935022000	-1.854121000	-0.180217000
1	1.738533000	-2.927480000	0.983628000
6	-1.962770000	-1.803723000	-1.480496000
1	-2.576510000	-1.553990000	-2.348703000
1	-1.054021000	-1.203365000	-1.513177000
1	-1.687442000	-2.859469000	-1.540324000
6	-3.983870000	-2.470305000	-0.176603000
1	-4.603352000	-2.259384000	0.699690000
1	-4.588186000	-2.321798000	-1.073001000
1	-3.667390000	-3.515146000	-0.130309000
6	-1.907122000	-1.818021000	1.048544000
1	-2.514624000	-1.796773000	1.957411000
1	-1.469917000	-2.816752000	0.974605000
1	-1.094427000	-1.097840000	1.144009000

I_B_3_a

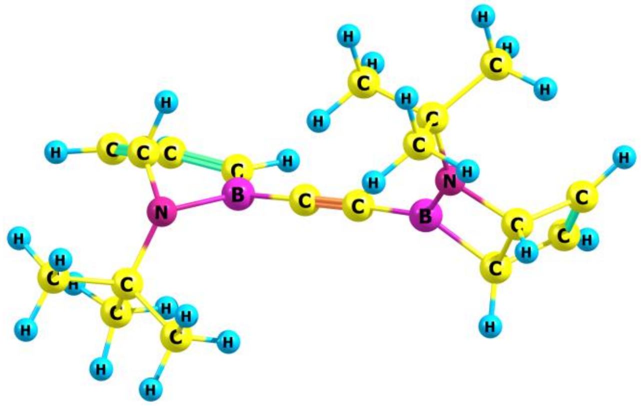

6	4.786052000	-1.772837000	0.122744000
6	4.107994000	-0.698688000	0.962578000
6	2.983013000	-1.753225000	1.232249000
6	3.832807000	-2.674640000	0.378169000
1	5.667439000	-1.766420000	-0.506427000
1	3.670952000	-3.695426000	0.051988000
1	4.657556000	-0.240015000	1.785919000
1	2.739669000	-2.052159000	2.250061000
5	2.075905000	-0.689091000	0.433387000
7	3.158029000	0.184127000	0.261220000
6	0.616690000	-0.615287000	0.008058000
6	-0.558483000	-0.593933000	-0.304052000
5	-2.039865000	-0.569155000	-0.677119000
6	-2.718567000	-1.493186000	-1.627304000
7	-2.882476000	0.654742000	-0.002636000
6	-4.053807000	-1.223222000	-2.113238000
1	-2.177750000	-2.338300000	-2.047880000
6	-3.335908000	0.715229000	-1.254404000
6	-4.504238000	0.016886000	-1.794069000
1	-4.663636000	-1.976158000	-2.604188000
1	-2.624594000	1.115760000	-1.980807000
1	-5.524156000	0.373401000	-1.773670000
6	3.502038000	1.407159000	-0.457373000
6	-3.712978000	0.424230000	1.208120000
6	3.960777000	2.467678000	0.549911000
1	4.831961000	2.121113000	1.111642000
1	4.237687000	3.389677000	0.032674000
1	3.158398000	2.689359000	1.256750000
6	4.634105000	1.104238000	-1.444724000
1	4.333869000	0.315658000	-2.138716000
1	4.885929000	2.000552000	-2.016525000
1	5.533948000	0.776143000	-0.917062000
6	2.263291000	1.894194000	-1.204418000
1	1.929178000	1.147294000	-1.927874000
1	1.441042000	2.081350000	-0.510488000
1	2.492764000	2.820993000	-1.735527000
6	-4.707718000	-0.744047000	1.155856000
1	-5.563601000	-0.539407000	0.513906000
1	-4.219697000	-1.657485000	0.811599000
1	-5.083608000	-0.916092000	2.167615000
6	-4.464806000	1.736766000	1.447205000
1	-3.763787000	2.568970000	1.533887000
1	-5.150250000	1.942575000	0.619655000
1	-5.050182000	1.669802000	2.366956000
6	-2.710921000	0.177039000	2.337533000
1	-1.968121000	0.975910000	2.365200000
1	-3.230504000	0.135181000	3.297226000
1	-2.185608000	-0.769267000	2.185620000

I_B_3_b

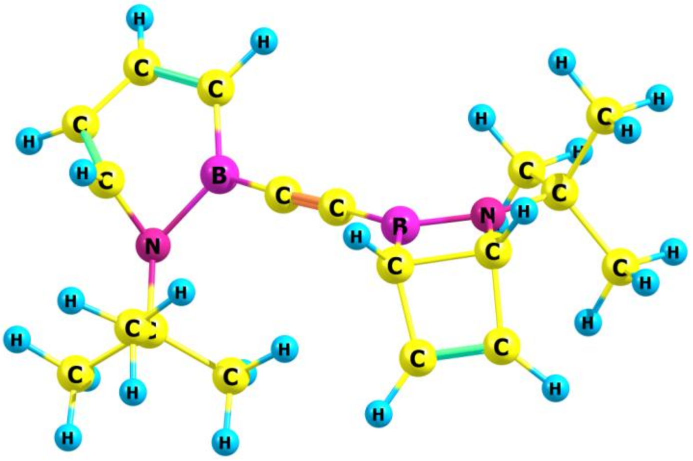

6	3.932722000	-2.158385000	0.184430000
6	3.751985000	-0.874730000	0.983651000
6	2.423443000	-1.506734000	1.518878000
6	2.809744000	-2.700465000	0.666098000
1	4.661587000	-2.467386000	-0.554440000
1	2.302086000	-3.640298000	0.481973000
1	4.539689000	-0.559146000	1.669296000
1	2.272726000	-1.648122000	2.587484000
5	1.758588000	-0.266335000	0.735051000
7	3.008234000	0.213444000	0.323388000
6	0.336621000	0.218761000	0.493714000
6	-0.822639000	0.546347000	0.329061000
5	-2.254180000	1.055530000	0.100394000
6	-2.609972000	2.468886000	0.434862000
7	-3.370611000	0.014127000	-0.380999000
6	-4.006495000	2.780114000	0.547617000
1	-1.850654000	3.209526000	0.676169000
6	-3.816240000	0.487276000	0.805187000
6	-4.727962000	1.641116000	0.634734000
1	-4.416029000	3.777953000	0.433181000
1	-3.627348000	0.006817000	1.767022000
1	-5.760786000	1.493315000	0.340070000
6	3.579242000	1.220194000	-0.566434000
6	-3.086202000	-1.428912000	-0.603994000
6	4.492757000	2.145300000	0.245530000
1	5.300284000	1.579952000	0.717630000
1	4.943779000	2.899975000	-0.403477000
1	3.920986000	2.650976000	1.026490000
6	4.390705000	0.517236000	-1.659705000
1	3.758814000	-0.179837000	-2.215079000
1	4.801296000	1.252361000	-2.355862000
1	5.225308000	-0.041615000	-1.227412000
6	2.437247000	2.021436000	-1.185666000
1	1.780449000	1.373492000	-1.770142000
1	1.834341000	2.498868000	-0.410300000
1	2.841696000	2.795772000	-1.841605000
6	-2.113901000	-1.544726000	-1.775316000
1	-2.479553000	-0.953606000	-2.617731000
1	-1.118833000	-1.192719000	-1.504812000
1	-2.039150000	-2.588709000	-2.088405000
6	-4.442676000	-2.021198000	-1.010063000
1	-5.172931000	-1.892933000	-0.205993000
1	-4.821680000	-1.526100000	-1.905650000
1	-4.337973000	-3.089793000	-1.212092000
6	-2.566005000	-2.172044000	0.631084000
1	-3.309159000	-2.187407000	1.432977000
1	-2.367912000	-3.210761000	0.356563000
1	-1.640663000	-1.736390000	1.008176000

TS2_B_3

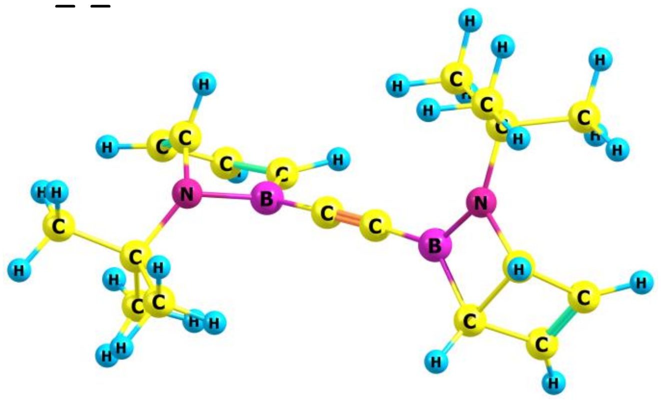

6	-4.632088000	1.481399000	-1.293145000
6	-4.064345000	1.288984000	0.106530000
6	-2.830504000	2.119155000	-0.382219000
6	-3.585625000	2.210095000	-1.694479000
1	-5.515389000	1.096208000	-1.787249000
1	-3.319148000	2.667443000	-2.640210000
1	-4.651119000	1.609276000	0.968664000
1	-2.546225000	3.037411000	0.128200000
5	-2.050709000	0.736330000	-0.111927000
7	-3.221293000	0.097844000	0.317041000
6	-0.611350000	0.262602000	-0.255754000
6	0.552680000	-0.066798000	-0.380217000
5	2.026935000	-0.454198000	-0.557350000
6	2.528128000	-1.280214000	-1.716185000
7	3.011643000	-0.247603000	0.588182000
6	3.936701000	-1.609034000	-1.952204000
1	1.787343000	-1.720928000	-2.384858000
6	3.483196000	-1.463321000	0.172892000
6	4.597183000	-1.622893000	-0.783313000
1	4.358548000	-1.754199000	-2.942133000
1	2.968750000	-2.348571000	0.539055000
1	5.655686000	-1.584281000	-0.567247000
6	-3.700889000	-1.234874000	0.670126000
6	3.826117000	0.977364000	0.788415000
6	-4.257891000	-1.205149000	2.097889000
1	-5.081851000	-0.491784000	2.180409000
1	-4.635647000	-2.191057000	2.379966000
1	-3.475965000	-0.914443000	2.802603000
6	-4.805302000	-1.643361000	-0.310416000
1	-4.431006000	-1.621609000	-1.336609000
1	-5.156331000	-2.652903000	-0.083879000
1	-5.659764000	-0.964633000	-0.239790000
6	-2.528710000	-2.208640000	0.584221000
1	-2.124896000	-2.240017000	-0.430075000
1	-1.723843000	-1.905480000	1.257213000
1	-2.859969000	-3.211828000	0.862547000
6	2.845311000	2.025889000	1.317276000
1	3.375368000	2.950887000	1.556066000
1	2.079675000	2.246522000	0.569372000
1	2.345613000	1.656935000	2.214417000
6	4.497642000	1.517545000	-0.483013000
1	5.317932000	0.884464000	-0.819635000
1	3.770979000	1.603080000	-1.294363000
1	4.900618000	2.512959000	-0.278856000
6	4.878860000	0.657372000	1.849411000
1	4.400612000	0.304791000	2.765517000
1	5.566182000	-0.116724000	1.495579000
1	5.465575000	1.549415000	2.080231000

TS2_B_3_e

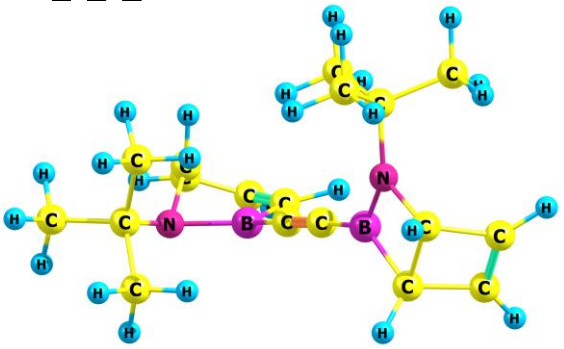

6	4.618026000	-1.495839000	0.815982000
6	3.936579000	-0.157762000	1.068656000
6	2.885020000	-0.959168000	1.906890000
6	3.729393000	-2.168871000	1.553636000
1	5.460829000	-1.786857000	0.201529000
1	3.601662000	-3.219590000	1.786111000
1	4.508776000	0.656388000	1.515480000
1	2.717207000	-0.720499000	2.955341000
5	1.881070000	-0.445660000	0.759230000
7	2.902079000	0.249145000	0.098934000
6	0.398609000	-0.626045000	0.459216000
6	-0.789186000	-0.791679000	0.261683000
5	-2.278729000	-1.092470000	0.021824000
6	-2.634458000	-2.259829000	-0.868125000
7	-3.367737000	-0.080431000	0.325067000
6	-4.008583000	-2.517494000	-1.269665000
1	-1.815740000	-2.838528000	-1.295059000
6	-3.562430000	-0.346842000	-1.019004000
6	-4.639642000	-1.336046000	-1.273948000
1	-4.428586000	-3.504061000	-1.429292000
1	-3.115647000	0.256708000	-1.810323000
1	-5.694580000	-1.096714000	-1.218240000
6	3.140035000	0.956006000	-1.156057000
6	-3.114928000	1.291888000	0.833475000
6	-2.189961000	2.127817000	-0.059656000
1	-2.630384000	2.300928000	-1.045346000
1	-2.039320000	3.106445000	0.402188000
1	-1.215770000	1.653759000	-0.182789000
6	-4.490675000	1.963608000	0.901190000
1	-5.156577000	1.395966000	1.553527000
1	-4.399039000	2.982163000	1.286024000
1	-4.941624000	2.013562000	-0.094574000
6	-2.537898000	1.161762000	2.240921000
1	-3.185687000	0.527411000	2.849682000
1	-1.538972000	0.724603000	2.220293000
1	-2.475395000	2.147341000	2.708114000
6	1.824186000	1.030538000	-1.925891000
1	1.446749000	0.030430000	-2.149354000
1	1.065249000	1.555468000	-1.341245000
1	1.974714000	1.568234000	-2.864830000
6	4.189337000	0.191066000	-1.969866000
1	4.358298000	0.687026000	-2.928608000
1	5.143169000	0.152519000	-1.436909000
1	3.856069000	-0.832630000	-2.155526000
6	3.649754000	2.367971000	-0.846945000
1	4.581455000	2.330432000	-0.276910000
1	3.843345000	2.913740000	-1.773634000
1	2.908632000	2.918935000	-0.263892000

P3

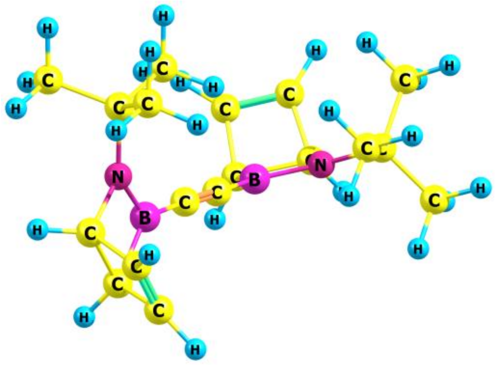

6	4.539180000	1.791699000	-0.462811000
6	4.209794000	0.384576000	-0.941030000
6	3.158579000	1.054160000	-1.888802000
6	3.656680000	2.356972000	-1.292682000
1	5.190080000	2.169429000	0.315799000
1	3.346355000	3.381790000	-1.459803000
1	5.006677000	-0.243886000	-1.341141000
1	3.215696000	0.910109000	-2.965957000
5	2.119592000	0.217425000	-0.989213000
7	3.145309000	-0.326504000	-0.208303000
6	0.605647000	0.051397000	-0.953516000
6	-0.605647000	-0.051396000	-0.953516000
5	-2.119592000	-0.217424000	-0.989213000
6	-3.158578000	-1.054160000	-1.888802000
7	-3.145309000	0.326504000	-0.208303000
6	-4.209794000	-0.384577000	-0.941030000
6	-3.656679000	-2.356972000	-1.292683000
1	-3.215695000	-0.910108000	-2.965958000
6	-4.539180000	-1.791699000	-0.462812000
1	-5.006677000	0.243886000	-1.341141000
1	-3.346353000	-3.381790000	-1.459804000
1	-5.190079000	-2.169430000	0.315798000
6	3.325662000	-1.121303000	1.003051000
6	-3.325663000	1.121303000	1.003052000
6	4.117904000	-0.298167000	2.023997000
1	3.598720000	0.637283000	2.245528000
1	4.238129000	-0.863095000	2.951302000
1	5.114394000	-0.058580000	1.642702000
6	4.096170000	-2.399524000	0.653864000
1	4.257231000	-3.004084000	1.549831000
1	3.536309000	-2.991845000	-0.072880000
1	5.073726000	-2.160878000	0.227266000
6	1.948628000	-1.472971000	1.559872000
1	1.391025000	-0.567682000	1.811058000
1	1.366150000	-2.035904000	0.827476000
1	2.057931000	-2.080228000	2.461352000
6	-1.948628000	1.472972000	1.559871000
1	-2.057932000	2.080229000	2.461351000
1	-1.391024000	0.567683000	1.811058000
1	-1.366151000	2.035905000	0.827475000
6	-4.117904000	0.298166000	2.023997000
1	-4.238128000	0.863094000	2.951303000
1	-5.114394000	0.058578000	1.642703000
1	-3.598719000	-0.637283000	2.245528000
6	-4.096171000	2.399523000	0.653865000
1	-4.257233000	3.004083000	1.549832000
1	-3.536312000	2.991844000	-0.072880000
1	-5.073728000	2.160876000	0.227267000

II. R = TBDMS

The lowest energy conformers used for the depiction of the potential energy surface (Fig. S50A) are highlighted on light green background.

R4

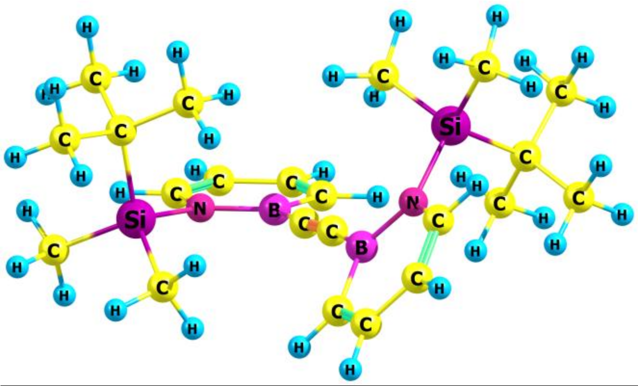

6	2.662987000	-3.362014000	-1.848877000
6	1.594903000	-2.510904000	-1.816663000
6	3.853839000	-2.046485000	-0.208097000
6	3.806767000	-3.126306000	-1.037453000
1	2.665442000	-4.236545000	-2.494331000
1	0.739429000	-2.715410000	-2.452444000
1	4.732056000	-1.882952000	0.405816000
1	4.653255000	-3.799604000	-1.063691000
7	2.837463000	-1.129569000	-0.092134000
5	1.649374000	-1.303842000	-0.900732000
6	0.479933000	-0.305027000	-0.845756000
6	-0.508847000	0.401721000	-0.870619000
6	-1.486195000	2.873806000	-1.028999000
6	-2.570655000	3.702323000	-0.967406000
1	-0.506255000	3.305536000	-1.204619000
6	-4.065270000	1.839499000	-0.599479000
6	-3.874914000	3.180419000	-0.747019000
1	-2.463611000	4.777335000	-1.086880000
1	-5.066458000	1.457236000	-0.436692000
1	-4.733696000	3.836675000	-0.698569000
5	-1.694459000	1.382302000	-0.855467000
7	-3.049772000	0.915640000	-0.647264000
14	3.112384000	0.291635000	0.998505000
14	-3.483140000	-0.817286000	-0.339058000
6	-5.281046000	-1.032111000	-0.849306000
1	-5.514282000	-2.100213000	-0.862121000
1	-5.999248000	-0.550077000	-0.183509000
1	-5.444651000	-0.650579000	-1.860987000
6	-2.460411000	-1.985823000	-1.383008000
1	-2.221062000	-1.541552000	-2.351790000
1	-1.518124000	-2.256507000	-0.906712000
1	-3.041288000	-2.896167000	-1.557749000
6	-3.287432000	-1.144538000	1.517606000
6	-3.687570000	-2.603247000	1.791197000
1	-3.049528000	-3.306193000	1.246828000
1	-3.585028000	-2.824502000	2.859843000
1	-4.727738000	-2.800161000	1.513653000
6	-1.836916000	-0.923045000	1.967026000
1	-1.523239000	0.113977000	1.817558000
1	-1.744535000	-1.150794000	3.036142000
1	-1.134008000	-1.559928000	1.422376000
6	-4.201068000	-0.204042000	2.316738000
1	-4.083633000	-0.394447000	3.389933000
1	-3.951205000	0.846473000	2.139030000
1	-5.257145000	-0.351995000	2.072799000
6	3.815006000	1.706838000	-0.046276000
6	4.371474000	-0.242323000	2.290323000
1	4.075981000	-1.186740000	2.755598000
1	5.387778000	-0.354284000	1.907459000
1	4.401839000	0.514362000	3.078884000
6	1.548606000	0.766672000	1.908407000
1	1.824506000	1.281324000	2.833469000

1	0.898488000	1.416784000	1.323081000
1	0.971390000	-0.122146000	2.175200000
6	4.155807000	2.871976000	0.897264000
1	4.559830000	3.712013000	0.320620000
1	3.270352000	3.234330000	1.428989000
1	4.908994000	2.590132000	1.639366000
6	5.088931000	1.243072000	-0.767264000
1	5.867385000	0.928418000	-0.065624000
1	4.884874000	0.411573000	-1.448503000
1	5.499512000	2.066465000	-1.363245000
6	2.798035000	2.184784000	-1.091410000
1	2.550505000	1.397332000	-1.807768000
1	1.862661000	2.513909000	-0.631408000
1	3.216845000	3.029781000	-1.651079000

TS2_A_4

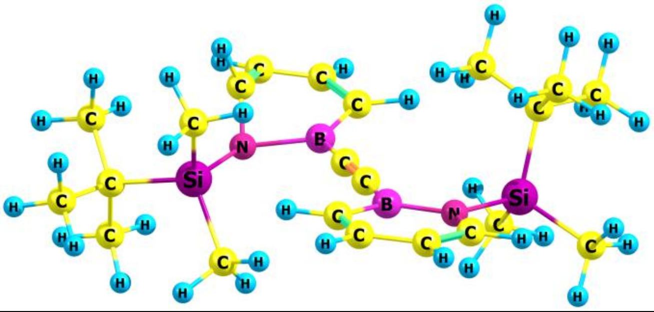

6	1.105644000	3.085942000	0.265025000
5	1.338467000	1.644891000	0.288949000
7	2.842028000	1.029873000	0.197392000
6	3.080243000	1.913972000	-0.744384000
6	3.355021000	3.326973000	-0.346472000
6	2.266890000	3.942296000	0.139840000
1	3.177761000	1.609623000	-1.796704000
1	0.113357000	3.514855000	0.349260000
1	2.284782000	4.968156000	0.494156000
1	4.382452000	3.670623000	-0.312793000
14	3.162712000	-0.734687000	0.006184000
6	2.381872000	-1.637715000	1.439912000
1	1.369700000	-1.967701000	1.206628000
1	2.983025000	-2.509657000	1.712149000
1	2.327043000	-0.979621000	2.310857000
6	2.569216000	-1.324037000	-1.670179000
1	3.141106000	-0.893329000	-2.496495000
1	2.670221000	-2.411278000	-1.735120000
1	1.514588000	-1.073634000	-1.806119000
6	5.057862000	-0.835183000	0.119988000
6	5.508485000	-0.507386000	1.550966000
1	6.601835000	-0.550474000	1.620007000
1	5.190783000	0.495621000	1.850200000
1	5.103056000	-1.219608000	2.274882000
6	5.484515000	-2.268443000	-0.237719000
1	5.003789000	-3.012827000	0.404541000
1	5.246742000	-2.513784000	-1.276912000
1	6.567545000	-2.378194000	-0.110528000
6	5.735842000	0.138112000	-0.852790000
1	6.821172000	-0.014967000	-0.836719000
1	5.402876000	-0.006158000	-1.885858000
1	5.559951000	1.181501000	-0.572589000
6	0.278769000	0.546375000	0.213981000
6	-0.582271000	-0.310198000	0.148044000
5	-1.592937000	-1.458122000	0.005742000
6	-1.154767000	-2.835829000	-0.452907000
7	-3.000255000	-1.261926000	0.285282000
6	-2.097492000	-3.815342000	-0.589089000
1	-0.119888000	-3.069884000	-0.684306000
6	-3.860335000	-2.317439000	0.111190000
14	-3.710193000	0.338892000	0.757570000
6	-3.465935000	-3.553780000	-0.305338000
1	-1.825798000	-4.815171000	-0.917720000
1	-4.907256000	-2.135329000	0.325054000
6	-5.328035000	-0.023649000	1.647200000
6	-2.605906000	1.253455000	1.958890000

6	-4.045981000	1.324780000	-0.825353000
1	-4.212234000	-4.329580000	-0.413128000
1	-5.682550000	0.900154000	2.112300000
1	-6.126898000	-0.389283000	0.999384000
1	-5.176262000	-0.753460000	2.447329000
1	-2.086286000	0.558386000	2.622372000
1	-1.845893000	1.854025000	1.459867000
1	-3.227974000	1.910174000	2.574033000
6	-4.705657000	2.654186000	-0.423372000
6	-2.748925000	1.616654000	-1.592560000
6	-4.996242000	0.538430000	-1.740194000
1	-4.053533000	3.249255000	0.223507000
1	-4.915312000	3.251470000	-1.318177000
1	-5.655253000	2.498756000	0.098020000
1	-2.266691000	0.697465000	-1.935319000
1	-2.976660000	2.226148000	-2.475329000
1	-2.018839000	2.159840000	-0.986839000
1	-5.190688000	1.111402000	-2.654360000
1	-4.563716000	-0.421513000	-2.038589000
1	-5.962246000	0.346593000	-1.263727000

P_A_4

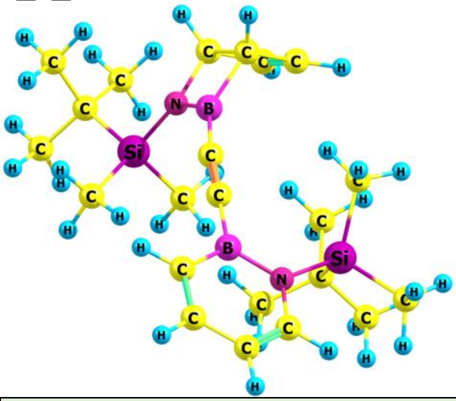

6	-2.681241000	-2.653661000	-0.974530000
6	-2.977219000	-1.319077000	-1.637059000
6	-1.660894000	-1.534581000	-2.457320000
6	-1.569589000	-2.843466000	-1.693320000
1	-3.157871000	-3.191017000	-0.163540000
1	-0.818020000	-3.624891000	-1.705875000
1	-3.933956000	-1.174654000	-2.139052000
1	-1.668630000	-1.557288000	-3.545395000
5	-1.207948000	-0.234848000	-1.630840000
7	-2.436011000	-0.112354000	-0.949745000
6	0.095888000	0.531577000	-1.455781000
6	1.200677000	0.989797000	-1.238309000
6	2.906679000	3.047754000	-1.092814000
6	4.131382000	3.500866000	-0.689275000
1	2.207794000	3.746004000	-1.540563000
6	4.779688000	1.302161000	0.080680000
6	5.075114000	2.621773000	-0.091907000
1	4.411752000	4.543660000	-0.812636000
1	5.513880000	0.645185000	0.530347000
1	6.042166000	2.984334000	0.230410000
5	2.575913000	1.586940000	-0.885730000
7	3.586034000	0.737712000	-0.292738000
6	-4.850834000	0.853122000	0.494673000
6	-2.415635000	-0.344806000	1.992838000
1	-1.326599000	-0.303017000	2.082084000
1	-2.846117000	0.011281000	2.933006000
1	-2.697355000	-1.393032000	1.861946000
6	-2.126612000	2.368414000	0.575365000
1	-1.044900000	2.257022000	0.465065000
1	-2.477430000	3.015173000	-0.232497000
1	-2.321063000	2.870241000	1.527016000
6	2.715977000	-1.858776000	-1.566995000
1	2.883401000	-2.937183000	-1.483085000
1	1.660797000	-1.685508000	-1.772651000
1	3.294023000	-1.498061000	-2.421961000
6	2.130516000	-1.287613000	1.493435000
6	5.008044000	-1.771947000	0.362426000

1	5.702510000	-1.554631000	-0.453748000
1	5.460520000	-1.441997000	1.299300000
1	4.904072000	-2.858968000	0.419949000
14	-2.961035000	0.696027000	0.528823000
14	3.314763000	-1.034003000	0.004272000
6	-5.300217000	1.769258000	1.643479000
1	-4.905588000	2.782812000	1.529000000
1	-6.394235000	1.839797000	1.660928000
1	-4.981066000	1.389136000	2.619026000
6	-5.305661000	1.464822000	-0.837659000
1	-5.024244000	0.840717000	-1.689677000
1	-6.396529000	1.576242000	-0.848249000
1	-4.871234000	2.456404000	-0.995165000
6	-5.501051000	-0.526872000	0.677870000
1	-6.593260000	-0.436048000	0.639887000
1	-5.203873000	-1.231598000	-0.104750000
1	-5.239720000	-0.967985000	1.644171000
6	2.855927000	-2.096731000	2.584088000
1	2.171222000	-2.274421000	3.420866000
1	3.192721000	-3.072147000	2.221366000
1	3.726407000	-1.564920000	2.977599000
6	1.685439000	0.049751000	2.105578000
1	1.080344000	0.633624000	1.407730000
1	1.079591000	-0.143322000	2.999174000
1	2.537565000	0.665885000	2.407963000
6	0.887163000	-2.078985000	1.055428000
1	0.248431000	-2.276859000	1.924455000
1	0.290509000	-1.527327000	0.324857000
1	1.150563000	-3.045598000	0.614254000

TS1_B_4

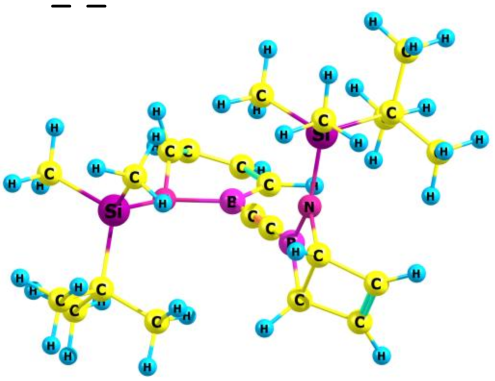

6	-3.915353000	-2.419416000	1.634691000
6	-3.065475000	-2.390106000	0.377458000
6	-1.831618000	-2.630663000	1.313866000
6	-2.862160000	-2.645827000	2.427001000
1	-4.967673000	-2.236831000	1.813252000
1	-2.749824000	-2.754660000	3.499423000
1	-3.298104000	-3.073638000	-0.439364000
1	-1.188658000	-3.500009000	1.183307000
5	-1.409564000	-1.185467000	0.751148000
7	-2.560031000	-1.056238000	-0.053133000
6	-0.226883000	-0.236251000	0.892853000
6	0.729813000	0.509951000	0.976572000
5	1.918758000	1.463331000	1.134183000
6	1.898376000	2.704860000	1.905923000
7	3.178325000	1.057551000	0.212784000
6	3.070439000	3.550796000	1.831222000
1	1.054781000	2.976964000	2.529335000
6	3.234565000	2.317068000	-0.167485000
6	3.837479000	3.323704000	0.755523000
1	3.355696000	4.236112000	2.623133000
1	2.967469000	2.609094000	-1.193726000
1	4.867307000	3.621424000	0.593770000
14	-3.267417000	0.161606000	-1.112248000
14	3.262613000	-0.322413000	-0.954180000
6	1.697608000	-0.573408000	-1.949959000
1	1.952901000	-1.069083000	-2.891539000
1	0.958996000	-1.175536000	-1.420478000
1	1.226753000	0.383942000	-2.189968000
6	3.791006000	-1.843299000	0.029580000
6	4.629514000	0.231467000	-2.117996000

1	4.941044000	-0.591753000	-2.766469000
1	4.288141000	1.041520000	-2.769339000
1	5.509104000	0.580155000	-1.571580000
6	-1.874166000	0.864114000	-2.148587000
1	-2.208392000	1.704688000	-2.762559000
1	-1.068298000	1.211464000	-1.495816000
1	-1.464914000	0.096917000	-2.810933000
6	-4.076525000	1.531558000	-0.080556000
6	-4.539174000	-0.730501000	-2.163924000
1	-4.989067000	-0.055767000	-2.896286000
1	-4.073799000	-1.553964000	-2.712099000
1	-5.345189000	-1.147574000	-1.553567000
6	3.811715000	-3.048133000	-0.925315000
1	4.492385000	-2.895511000	-1.769023000
1	4.153158000	-3.939767000	-0.387426000
1	2.816486000	-3.264740000	-1.324611000
6	5.200438000	-1.614372000	0.596019000
1	5.941345000	-1.481413000	-0.197668000
1	5.231728000	-0.734970000	1.245678000
1	5.505603000	-2.482528000	1.191180000
6	2.824611000	-2.133775000	1.185936000
1	3.158219000	-3.031036000	1.720664000
1	2.788604000	-1.308463000	1.901724000
1	1.804162000	-2.307824000	0.835692000
6	-2.986531000	2.339380000	0.639221000
1	-2.329739000	2.854913000	-0.067297000
1	-3.447213000	3.100210000	1.280735000
1	-2.359083000	1.704136000	1.271743000
6	-5.013625000	0.914012000	0.965640000
1	-5.797194000	0.303109000	0.504757000
1	-4.460715000	0.286309000	1.669642000
1	-5.509898000	1.705825000	1.539599000
6	-4.881680000	2.463451000	-0.998043000
1	-5.308125000	3.287573000	-0.413982000
1	-4.257719000	2.906910000	-1.780538000
1	-5.711270000	1.938936000	-1.481426000

TS2_B_4

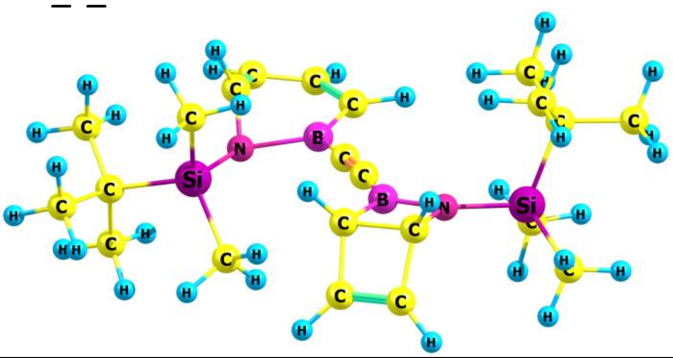

6	-1.655853000	3.298508000	-0.628198000
5	-1.706232000	1.852191000	-0.453974000
7	-3.123988000	1.065719000	-0.280933000
6	-3.486169000	2.033192000	0.530292000
6	-3.927948000	3.333440000	-0.056577000
6	-2.917627000	4.010212000	-0.621713000
1	-3.564183000	1.865293000	1.614362000
1	-0.722223000	3.830077000	-0.770332000
1	-3.057000000	4.968839000	-1.111188000
1	-4.989249000	3.541071000	-0.129284000
14	-3.233056000	-0.678143000	0.170305000
6	-2.290202000	-1.695655000	-1.079924000
1	-1.305751000	-1.972543000	-0.700711000
1	-2.842759000	-2.606487000	-1.325664000
1	-2.143420000	-1.127022000	-2.001234000
6	-2.631881000	-0.925398000	1.927590000
1	-3.272394000	-0.443391000	2.670735000
1	-2.607637000	-1.994087000	2.159032000
1	-1.617012000	-0.535214000	2.033526000
6	-5.097179000	-1.026228000	0.040744000
6	-5.538423000	-0.947389000	-1.428089000
1	-6.614832000	-1.139715000	-1.507610000
1	-5.339818000	0.040937000	-1.852369000
1	-5.024162000	-1.688863000	-2.045708000

6	-5.359439000	-2.441147000	0.582278000
1	-4.774683000	-3.199339000	0.052277000
1	-5.124619000	-2.516338000	1.648075000
1	-6.417356000	-2.698543000	0.457200000
6	-5.917664000	-0.023785000	0.862621000
1	-6.976193000	-0.308050000	0.847148000
1	-5.605643000	0.009110000	1.911560000
1	-5.856864000	0.987787000	0.448934000
6	-0.526499000	0.902624000	-0.240252000
6	0.383538000	0.112631000	-0.071486000
5	1.441452000	-0.956376000	0.147390000
6	1.423360000	-2.491585000	0.635138000
7	2.841087000	-0.918300000	-0.001229000
6	2.972696000	-2.329626000	0.453929000
6	1.552893000	-3.536713000	-0.457357000
1	0.994781000	-2.780385000	1.593873000
14	4.074275000	0.172492000	-0.624424000
6	2.870064000	-3.384280000	-0.631587000
1	3.657686000	-2.484811000	1.288148000
1	0.805905000	-4.147325000	-0.952076000
6	4.774882000	1.179966000	0.821362000
6	5.398002000	-0.911655000	-1.395151000
6	3.264045000	1.289576000	-1.885832000
1	3.590203000	-3.782831000	-1.335285000
6	6.022413000	1.954005000	0.373430000
6	3.702864000	2.167841000	1.303953000
6	5.147589000	0.239391000	1.975676000
1	4.969270000	-1.515664000	-2.199168000
1	6.204292000	-0.310582000	-1.822742000
1	5.840363000	-1.592803000	-0.662491000
1	2.959763000	0.715871000	-2.764611000
1	2.368280000	1.756318000	-1.468432000
1	3.943362000	2.080741000	-2.214441000
1	5.810489000	2.616688000	-0.471706000
1	6.393420000	2.579219000	1.194278000
1	6.832774000	1.279828000	0.080696000
1	2.772157000	1.656857000	1.571036000
1	4.056990000	2.705525000	2.191653000
1	3.466891000	2.911437000	0.537542000
1	5.575578000	0.813009000	2.806534000
1	4.268338000	-0.288965000	2.355253000
1	5.891624000	-0.505111000	1.673842000

P4

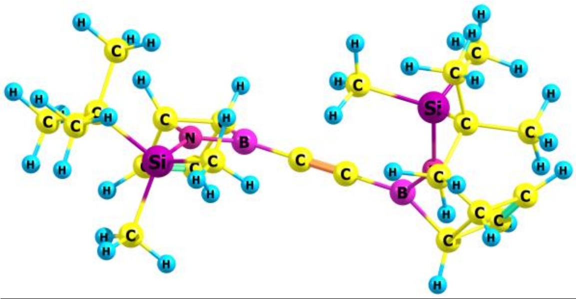

6	4.775645000	-2.584669000	0.303739000
6	4.056454000	-1.519953000	1.111704000
6	2.982956000	-2.612634000	1.434112000
6	3.867130000	-3.520530000	0.599021000
1	5.649077000	-2.555041000	-0.336149000
1	3.751462000	-4.559200000	0.312163000
1	4.588948000	-1.005636000	1.911536000
1	2.755784000	-2.896384000	2.460010000
5	2.045850000	-1.609095000	0.604792000
7	3.077686000	-0.678772000	0.365228000
6	0.584161000	-1.589120000	0.168221000
6	-0.584161000	-1.589120000	-0.168222000
6	4.595655000	1.864321000	0.068070000
6	4.013352000	0.019783000	-2.347550000
1	3.255909000	-0.595205000	-2.840442000
1	4.293506000	0.825173000	-3.032128000
1	4.893610000	-0.606924000	-2.183562000
6	1.685423000	1.514071000	-0.977844000
1	0.938018000	0.791364000	-1.314159000

1	1.320042000	1.957592000	-0.048014000
1	1.761151000	2.303965000	-1.730012000
14	3.341249000	0.681969000	-0.725257000
6	4.619996000	3.178126000	-0.728894000
1	3.655564000	3.692225000	-0.688492000
1	5.374426000	3.855736000	-0.311973000
1	4.873261000	3.014238000	-1.781166000
6	4.190925000	2.168719000	1.517115000
1	4.183643000	1.267996000	2.136062000
1	4.897249000	2.878305000	1.964385000
1	3.194060000	2.616121000	1.571837000
6	6.001704000	1.245213000	0.042990000
1	6.716378000	1.915412000	0.535531000
1	6.041693000	0.283551000	0.563806000
1	6.350353000	1.086078000	-0.981363000
5	-2.045850000	-1.609095000	-0.604793000
6	-2.982956000	-2.612634000	-1.434111000
7	-3.077685000	-0.678772000	-0.365229000
6	-4.056453000	-1.519953000	-1.111705000
6	-3.867130000	-3.520529000	-0.599019000
1	-2.755784000	-2.896385000	-2.460009000
14	-3.341248000	0.681969000	0.725256000
6	-4.775645000	-2.584669000	-0.303738000
1	-4.588948000	-1.005637000	-1.911536000
1	-3.751462000	-4.559199000	-0.312160000
6	-4.595657000	1.864320000	-0.068069000
6	-4.013347000	0.019783000	2.347550000
6	-1.685422000	1.514074000	0.977839000
1	-5.649076000	-2.555040000	0.336150000
6	-4.619998000	3.178125000	0.728895000
6	-4.190930000	2.168718000	-1.517115000
6	-6.001705000	1.245210000	-0.042987000
1	-3.255902000	-0.595203000	2.840441000
1	-4.293501000	0.825173000	3.032129000
1	-4.893604000	-0.606925000	2.183565000
1	-0.938016000	0.791368000	1.314153000
1	-1.320044000	1.957595000	0.048009000
1	-1.761149000	2.303967000	1.730008000
1	-3.655567000	3.692225000	0.688492000
1	-5.374429000	3.855734000	0.311975000
1	-4.873262000	3.014237000	1.781167000
1	-4.183647000	1.267996000	-2.136062000
1	-4.897256000	2.878304000	-1.964383000
1	-3.194065000	2.616122000	-1.571839000
1	-6.716381000	1.915408000	-0.535527000
1	-6.041693000	0.283548000	-0.563804000
1	-6.350352000	1.086074000	0.981366000

11. References

1. R. C. Richter, S. M. Biebl, R. Einholz, J. Walz, C. Maichle-Mössmer, M. Ströbele, H. F. Bettinger, I. Fleischer, *Angew. Chem. Int. Ed.*, 2024, **63**, e202405818.
2. A. J. V. Marwitz, A. N. Lamm, L. N. Zakharov, M. Vasiliu, D. A. Dixon, S.-Y. Liu, *Chem. Sci.*, 2012, **3**, 825.
3. Y. Zhao and D. Truhlar, *Theor. Chem. Acc.*, 2008, **120**, 215-241.
4. R. Krishnan, J. S. Binkley, R. Seeger and J. A. Pople, *J. Chem. Phys.*, 1980, **72**, 650-654.
5. T. Yanai, D. P. Tew, N. C. Handy, *Chem. Phys. Lett.* 2004, 393, 51-57.
6. M. J. Frisch, G. W. Trucks, H. B. Schlegel, G. E. Scuseria, M. A. Robb, J. R. Cheeseman, G. Scalmani, V. Barone, G. A. Petersson, H. Nakatsuji, X. Li, M. Caricato, A. V. Marenich, J. Bloino, B. G. Janesko, R. Gomperts, B. Mennucci, H. P. Hratchian, J. V. Ortiz, A. F. Izmaylov, J. L. Sonnenberg, Williams, F. Ding, F. Lipparini, F. Egidi, J. Goings, B. Peng, A. Petrone, T. Henderson, D. Ranasinghe, V. G. Zakrzewski, J. Gao, N. Rega, G. Zheng, W. Liang, M. Hada, M. Ehara, K. Toyota, R. Fukuda, J. Hasegawa, M. Ishida, T. Nakajima, Y. Honda, O. Kitao, H. Nakai, T. Vreven, K. Throssell, J. A. Montgomery Jr., J. E. Peralta, F. Ogliaro, M. J. Bearpark, J. J. Heyd, E. N. Brothers, K. N. Kudin, V. N. Staroverov, T. A. Keith, R. Kobayashi, J. Normand, K. Raghavachari, A. P. Rendell, J. C. Burant, S. S. Iyengar, J. Tomasi, M. Cossi, J. M. Millam, M. Klene, C. Adamo, R. Cammi, J. W. Ochterski, R. L. Martin, K. Morokuma, O. Farkas, J. B. Foresman and D. J. Fox, Gaussian 16 Rev. C.01, Wallingford, CT, 2016.
7. C. Riplinger and F. Neese, *J. Chem. Phys.*, 2013, **138**, 034106.
8. C. Riplinger, P. Pinski, U. Becker, E. F. Valeev and F. Neese, *J. Chem. Phys.*, 2016, **144**, 024109.
9. C. Riplinger, B. Sandhoefer, A. Hansen and F. Neese, *J. Chem. Phys.*, 2013, **139**, 134101.
10. F. Neese, *WIREs Comput. Mol. Sci.*, 2012, **2**, 73-78.
11. F. Neese, *WIREs Comput. Mol. Sci.*, 2018, **8**, e1327.
12. F. Neese, *WIREs Comput. Mol. Sci.*, 2022, **12**, e1606.
13. T. H. Dunning, *J. Chem. Phys.*, 1989, **90**, 1007.
14. D. E. Woon and T. H. Dunning, *J. Chem. Phys.*, 1993, **98**, 1358-1371.
15. F. Weigend, A. Köhn and C. Hättig, *J. Chem. Phys.*, 2002, **116**, 3175-3183.
16. H. F. Bettinger and O. Hauler, *Beilstein J. Org. Chem.*, 2013, **9**, 761-766.
17. K. Edel, X. Yang, J. S. A. Ishibashi, A. N. Lamm, C. Maichle-Mössmer, X. Giustra Zachary, S. Y. Liu and H. F. Bettinger, *Angew. Chem. Int. Ed.*, 2018, **57**, 5296-5300.
